# The investigation of molecular epidemiological characteristics and resistance mechanism of tigecycline resistant *Klebsiella pneumoniae* from a large teaching hospital in southwest China, Chongqing

**DOI:** 10.3389/fcimb.2025.1540967

**Published:** 2025-03-13

**Authors:** Yuqiong Li, Shiyu Tang, Qi Han, Peiwen Xia, Tingting Si, Yuanyuan Song, Yun Xia

**Affiliations:** Department of Laboratory Medicine, The First Affiliated Hospital of Chongqing Medical University, Chongqing, China

**Keywords:** resistance mechanism, *Klebsiella pneumoniae*, tigecycline-resistance, tet(A), RND efflux pump

## Abstract

**Background:**

*Klebsiella pneumoniae* is one of the main pathogens of nosocomial infection, among which carbapenems can be used for multidrug-resistant *Klebsiella pneumoniae*. However, in the past decade, the resistance rate of carbapenem-resistant *Klebsiella pneumoniae* has increased yearly. Tigecycline has good antibacterial activity in treating severe bacterial infections, but the reports of tigecycline resistance are increasing. This study aimed to investigate the mechanism of drug resistance and epidemiological characteristics of tigecycline-resistant *Klebsiella pneumoniae* (TRKP) in a large teaching hospital in southwest China, Chongqing.

**Methods:**

We isolated 30 TRKP strains from this hospital between August 2021 and December 2023. By PCR and sequencing, we examined the presence and mutation rates of genes associated with tigecycline resistance, including *acrR, oqxR, ramR, tmexC, tet(x), tet(A), tet(L)*, and *rpsj*, and performed efflux pump inhibition experiments to verify efflux pump activity. At the same time, real-time RT-PCR was used to detect the expression levels of efflux pump genes (*acrB* and *oqxB*) and *ramA.* To investigate the prevalence trend of TRKP in our hospital, we performed multi-site sequence typing (MLST) analysis.

**Results:**

The mutation rates of *ramR* (73.3%) and *tet(A)* (63.3%) were significant. In efflux pump inhibition experiments, PaβN could reverse the resistance of 29 TRKP strains (96.7%) to tigecycline. Real-time RT-PCR results showed that *acrB* and *ramA* genes were up-regulated in 22 strains, while *oqxB* genes were overexpressed in only 4 strains. MLST analysis showed that these strains could be divided into 25 different ST subtypes, indicating that no outbreak of TRKP occurred in our hospital. In addition, two tmexCD-torpj positive strains, ST661 and ST1561, were identified for the first time.

**Conclusion:**

The efflux pump *acrB* and *tet(A)* mutations are the primary mechanisms of resistance to *tigecycline-resistant Klebsiella pneumoniae* at our hospital. The *ramR* mutation can mediate efflux pump activity of *acrB* by up-regulating *ramA* overexpression.

## 1 Introduction

With the rise of *Klebsiella pneumoniae*, a widely distributed pathogen, critical diseases such as endophthalmitis and bloodstream infections have become common (Lee et al., 2006, 2015; Yang et al., 2021). Carbapenems are often relied on for treating multidrug-resistant (MDR) infections; however, their overuse has resulted in the development of carbapenem-resistant *Klebsiella pneumoniae* (CRKP). Treatments available are increasingly limited, with only last-resort antibiotics like colistin and tigecycline remaining (Sheu et al., 2019). As the first glycylcycline antibiotic, it is a rare effective treatment for difficult-to-treat conditions, particularly those caused by CRKP (Seifert et al., 2018). Like tetracyclines, tigecycline binds reversibly to the 30S ribosomal subunit, disrupting aminoacyl-tRNA function and inhibiting bacterial translation (Pournaras et al., 2016). Tigecycline-resistant strains of K. pneumoniae have emerged rapidly since the clinical use of tigecycline, a problem likely exacerbated by the overuse of antibiotics, which has the potential to complicate treatment and pose a significant public health risk.

The current resistance mechanism of tigecycline is mainly related to the overexpression of RND efflux pumps, including AcrAB, OqxAB, MexAB-OprM, and Tmexd-toprJ (Pournaras et al., 2016; Chen et al., 2017; Lv et al., 2020; Avakh et al., 2023). The AcrAB-TolC pump is driven by the global transcriptional activator *RamA* and local inhibitory factor AcrR (Villa et al., 2014). The presence of a mutant in *ramR*, a local inhibitor of *ramA*, leads to elevated *ramA* expression and dysregulation of AcrAB expression, which ultimately leads to tigecycline resistance (Xu et al., 2021). Similarly, inactivation of *OqxR* enhances *OqxAB* transcription (Wan Nur Ismah et al., 2018). Furthermore, it has been demonstrated that mutations such as V57L in the *rpsj* gene can contribute to resistance even in the absence of *ramR* mutations (Herrera et al., 2021). The tet protein also has several known tigecycline resistance mechanisms, including *tet(A), tet(L), tet(X)*, and *tet(M)* (Fiedler et al., 2016; Fan et al., 2024; Zou et al., 2024). Among them, mutations in the ribosome protection protein *tet(M)* can modify drug resistance by changing the binding site. Tetracycline mobile inactivating enzyme *tet(X)* and its variants can exist on a variety of mobile genetic elements, mediate the rapid spread of tigecycline resistance genes through horizontal transfer, and exist stably in drug-resistant strains at a very low adaptive cost, and significantly increase the level of resistance to tigecycline (Song et al., 2020; Hsieh et al., 2021).

The growing tigecycline resistance has significantly limited clinical treatment options for multidrug-resistant *K. pneumoniae*. Hence, it is crucial to examine TRKP isolates, particularly in regions like Southwest China, where data is lacking. This study aimed to evaluate the phenotypic characteristics, molecular prevalence and tigecycline resistance mechanism of TRKP isolates from southwest China. In brief, a drug susceptibility test was performed on clinically isolated *K. pneumoniae*, and the genetic relationship of TRKP isolates was studied using multi-site sequence typing (MLST) technique. At the same time, efflux pump inhibition assay was performed to verify efflux pump activity. Using PCR, DNA sequencing technology and reverse transcription PCR (RT-PCR), the determinants of drug resistance including tigecycline resistance genes, pump genes and their regulatory factors were studied.

## 2 Materials and methods

### 2.1 Identification of strains and drug susceptibility test

Between August 2021 and December 2023, we used the Vitek-2 system (Biomérieux, France) to isolate TRKP strains from southwest China. Through the MALDI-TOF mass spectrometry (Biomérieux, Craponne, France), all separate strains are recognized as K. pneumonia. The minimum inhibitory concentration (MIC) was determined with cation-regulated Mueller Hinton broth (CAMHB), and Escherichia coli ATCC 25922 was used as the control strain. Since CLSI has not yet determined the breakpoint of tigecycline, this study referred to FDA’s sensitivity guidelines for Enterobacteria (sensitivity ≤2mg/L, intermediate 4mg/L, resistance ≥ 8 mg/L) (Zheng et al., 2018). Following CLSI-2023 recommendations, the VITEK-2 system was used to carry out further susceptibility to antimicrobial investigation, and the outcomes were interpreted appropriately.

### 2.2 Identification of determinants of tigecycline resistance

As indicated in **Table 1**, PCR was performed using gene-specific primers to test TRKP clinical isolates for the tigecycline resistance determinants *acrR*, *ramR*, *rpsj*, *oqxR*, *tet(L)*, *tet(A)*, *tmexC* and *tet(X)*. In order to provide A reliable comparison basis after sequencing, so as to accurately identify and locate the mutation sites generated by our experiment, the sequences were compared to those of wild-type reference strains E. coli plasmid RP1 for *tet(A)* detection (GenBank accession number X00006)] and [*K. pneumoniae* MGH78578 (GenBank accession number CP000647) for other detection to show the mutations. A total of 35 cycles of PCR reaction conditions were as follows: predenaturation at 94°C for 5 minutes; denaturation at 94°C for 30 seconds, annealing at 56°C for 30 seconds, and extension at 72°C for 40 seconds; and finally, extension at 72°C for 5 minutes.

**Table 1:** Clinical Characteristics of TRKP Isolates (n = 30).

Characteristics	No. (%)
Type	
Sputum	11(36.7)
Urine	9(30)
Bronchoalveolar lavage	2(6.7)
bile	4(13.3)
Drainage fluid	1(3.3)
Blood	1(3.3)
Other	2(6.7)
Department distribution	
Intensive Care Unit	10(33.3)
Urinary Surgery	4(13.3)
Hepatobiliary Surgery	4(13.3)
Rehabilitation department	3(10)
Neurology Department	2(6.7)
Gastrointestinal Surgery	2(6.7)
kidney internal medicine	2(6.7)
Oncology Department	1(3.3)
Orthopedics	1(3.3)
geriatric department	1(3.3)
Sex	
Male	20(66.7)
Female	10(33.3)
**Age (mean ± SD)**	68.83 ± 17.73
**Use of tigecycline**	6(20.0)
**Death rate**	11(37.7)
**Day(s) of hospitalization**	69.07 ± 14.53

TRKP, Tigecycline-resistant K. pneumoniae.

### 2.3 Efflux inhibition assay

Utilizing the efflux pump inhibitor (EPI) Phe-Arg-β-naphthylamide (PAβN, MedChemExpress), the efflux pump activity in isolates of *K. pneumoniae* resistant to tigecycline was investigated. The MIC of tigecycline both with and without PAβN at a concentration of 50 mg/l was determined using the broth microdilution technique. When EPIs are present, a quadruple or higher decline in the MIC is deemed to be evidence of efflux pump efficiency.

### 2.4 Quantitative reverse transcription PCR

Using qRT-PCR, the expression levels of the transcriptional regulator genes *ramA* and the efflux pump genes *acrB* and *oqxB* were evaluated. As directed by the manufacturer, total RNA from bacteria has been extracted using the RNAprep Pure Cell/Bacteria Kit (Tiangen, Beijing, China). The PrimeScriptTM FAST RT Reagent Kit with gDNA Eraser (TaKaRa, Kyoto, Japan) was subsequently utilized to synthesize cDNA. Each sample was analyzed in triplicate. Normalization of the target gene’s mRNA expression was done using the housekeeping gene, *rpob*. The relative expression level of tigecycline-sensitive bacteria ATCC 25922 was measured as a negative control. Cycle threshold (Ct) values were measured by the qRT-PCR program, and analysis of results was carried out using the 2-ΔΔCt method.

### 2.5 Multilocus sequence typing

MLST has been used to analyze the genetic relationships of the strains. We download primers from PubMLST website (http://www.pasteur.fr/recherche/genopole/PF8 MLST/Kpneumoniae HTML) for seven housekeeping genes (*gapA, infB, mdh, pgi, phoE, rpoB*, and *tonB*), amplify and sequence the genes, and then analyze them using the MLST database. The analysis utilized the PubMLST site (http://www.pasteur.fr/recherche/genopole/PF8 MLST/Kpneumoniae HTML), 7 housekeeping gene synthesis listed on the website of the primer. The genes were amplified and sequenced, with subsequent analysis performed using the MLST database (Veleba et al., 2012). Sequence types (STs) were classified into clonal complexes (CCs) using the eBURST algorithm.

Statistical methods GraphPad Prism software V. 9.5.0 (GraphPad Software Inc, San Diego, CA) was used to statistically analyze the correlation between anti-tigecycline resistance and *acrB*, *oqxB*, and *ramA* expression levels. The calculation of gene expression differences between the groups was based on the Mann–Whitney U test and a P value less than 0.05 was considered statistically significant.

## 3 Results

### 3.1 Clinical characteristics of TRKP isolates

Analysis of specimen sources showed that most TRKP isolates were derived from urine (30.0%) and respiratory secretions (43.4%), of which sputum accounted for 36.7% and bronchoalveolar lavage fluid accounted for 6.7% (see **Table 1**). Of these 30 isolates, almost a third of the samples were taken from intensive care units (ICU). The TRKP strain affected 20 cases (66.7%) in males and 10 cases (33.3%) in women, with a median age of 68 years. (**Table 1**) All clinical specimens have been identified as *K. pneumoniae* by Matrix-Assisted Laser Desorption/Ionization Time-of-Flight Mass Spectrometry (bioMérieux, Marcy-l’Étoile, France).

### 3.2 Antibiotic resistance profile and Efflux pump inhibition assay in TRKP isolates

In this study, the tigecycline drug sensitivity and related characteristics of TRKP clinical isolates were detected (**Table 2**). Based on FDA guidelines, isolates with tigecycline MICs ≥8 μg/ml were classified as resistant. Among the 30 isolates, 50% had MICs of 8 μg/ml, 30% had MICs of 16 μg/ml, and 20% had MICs of 32 μg/ml. All TRKP isolates were resistant to minocycline. These isolates were also co-resistant ciprofloxacin (25/30, 83.4%), levofloxacin (21/30,70.0%), cefoperazone (19/30, 63.4%), cefepime (19/30, 63.4%), ceftriaxone (18/30, 60.0%), aztreonam (13/30, 43.3%), Ceftazidme (12/30, 40.0%), cefoxitin (11/30, 36.7%), gentamicin (5/30, 16.7%), and amikacin (4/30, 13.4%) were also co-resistant, suggesting that the majority of the strains were MDR. However, no colistin and meropenem resistant isolates were found. We conducted efflux pump inhibition experiments to investigate the mechanism of tigecycline resistance in *K. pneumoniae*. After exposure to efflux pump inhibitor (EPI) phenylalanine-arginine-β-naphthylamide (PAβN), 96.6% of 30 tigecycline-resistant strains (MIC≥8 mg/L) recovered their sensitivity. Among the 30 tigecycline-resistant isolates (MIC ≥8 mg/L), one isolate exhibited a 32-fold reduction, 11 isolates had a 16-fold reduction, 15 showed an eightfold reduction, two isolates showed a fourfold reduction, and one isolate’s tigecycline MIC remained unchanged With PaβN. The effects of PaβN on mic of tigecycline are shown in **Table 2**.

**Table 2:** TGC resistance determinants mutation(s) of TRKP isolates examined in the present study.

		TGC		Presence of TGC resistance determinants [mutation(s) occurring in nucleotide or protein sequence]^a^							
Isolate	TGC	+PAβN	ST	*ramR*	*acrR*	*oqxR*	*Tet(A)*	*tet(X)*	*tet(L)*	*tmexc*	*rpsJ*
TR1	8	1	6148	+	+	+(E24R)	–	–	–	–	+
TR2	8	1	15	+(A19V, K63M)	+	+	+ (type 1)	–	–	–	+
TR3	8	2	15	+(A19V, K63M)	+	+	+ (type 1)	–	–	–	+
TR4	8	1	65	–	+	+	–	–	–	–	+
TR5	8	0.5	1838	+	+	+	–	–	–	–	+
TR6	8	1	1308	–	+(S215P, Y114F,V165I)	–	–	–	–	–	+
TR7	8	2	4106	+(11nt Δ(365-375))	+	+	+ (type 1)	–	–	–	+
TR8	8	1	45	+	+	+	+ (type 1)	–	–	–	+
TR9	8	1	893	+(R121P)	+	+	+ (type 3)	–	–	–	+
TR10	8	1	307	+	+	–	+ (type 1)	–	–	–	+
TR11	8	1	307	+	+	–	+ (type 1)	–	–	–	+
TR12	8	1	307	+(S157L)	+	+	–	–	–	–	+
TR13	8	0.5	1561	+(A2G)	–	+	+ (type 1)	–	–	+	+
TR14	8	1	3096	+(stop194K)	+	+	+ (type 1)	–	–	–	+
TR15	8	0.5	875	+10nt Δ (332-341))	+	+	–	–	–	–	+
TR16	16	1	23	+	+	+	–	–	–	–	+
TR17	16	1	700	+(E182stop)	+	+	+ (type 1)	–	–	–	+
TR18	16	2	6092	+(A34V)	+(E200V)	+	+ (type 1)	–	–	–	+
TR19	16	0.5	101	+(1nt Δ(57bp))	+	+(C100Y)	–	–	–	–	+
TR20	16	2	661	+	+	–	–	–	–	+	+
TR21	16	1	307	–	+	–	+ (type 1)	–	–	–	+
TR22	16	1	3368	–	–	+	+ (type 1)	–	–	–	+
TR23	16	16	11	+(8nt Δ(128-135bp))	–	–	+ (type 1)	–	–	–	+
TR24	16	2	37	+(E41stop)	+	+	+ (type 1)	–	–	–	+
TR25	32	2	485	+	+	+	+ (type 1)	–	–	–	+
TR26	32	2	29	+(Y59H, I141T)	+	+	+ (type 1)	–	–	–	+
TR27	32	4	65	+(I141T)	+	+	+ (type 1)	–	–	–	+
TR28	32	2	25	+(C292ins-frameshift mutation, E182*)	+	+	–	–	–	–	+
TR29	32	2	96	–	–	+(2nt Δ (226-227))	–	–	–	–	+
TR30	32	4	5387	+(Y147C,G151D,A183T)	+	+	+ (type 3)	–	–	–	+

a Genetic determinants of resistance were detected by performing PCR with gene-specific primers. Mutations were identified by comparison with wild-type reference sequences [Klebsiella pneumoniae MGH78578 (CP000647) for ramR, acrR, oqxR and rpsJ, Escherichia coli for tet(A) (X00006)]. +, presence of target gene and no change in the nucleotide or amino acid sequence; −, absence of target gene; Δ, deletion; bp, base pair.

### 3.3 Identifying and sequencing determinants of tigecycline resistance in clinical isolates of TRKP.

To explore the mechanisms underlying tigecycline resistance in TRKP, we identified potential tigecycline resistance determinants through PCR and sequencing, specifically including *ramR*, *acrR*, *oqxR*, *tet(A)*, *tet(X)*, *tet(L), tmexC*, and *rpsj* (**Table 2**). Using published primers (**Supplementary Table S1**), *rpsj* gene was detected in all isolates. *oqxR* and *acrR* were detected in 26 isolates (86.6%), *ramR* was detected in 25 isolates (83.3%), and *tet(A)* was detected in 19 isolates (63.3%). No strains carrying *tet(X)* and *tet(L)* were detected(**Table 2**), Sequence alignment diagrams containing protein mutations are shown in **Supplementary Figures S1**-**S4**.

**Figure 1:**
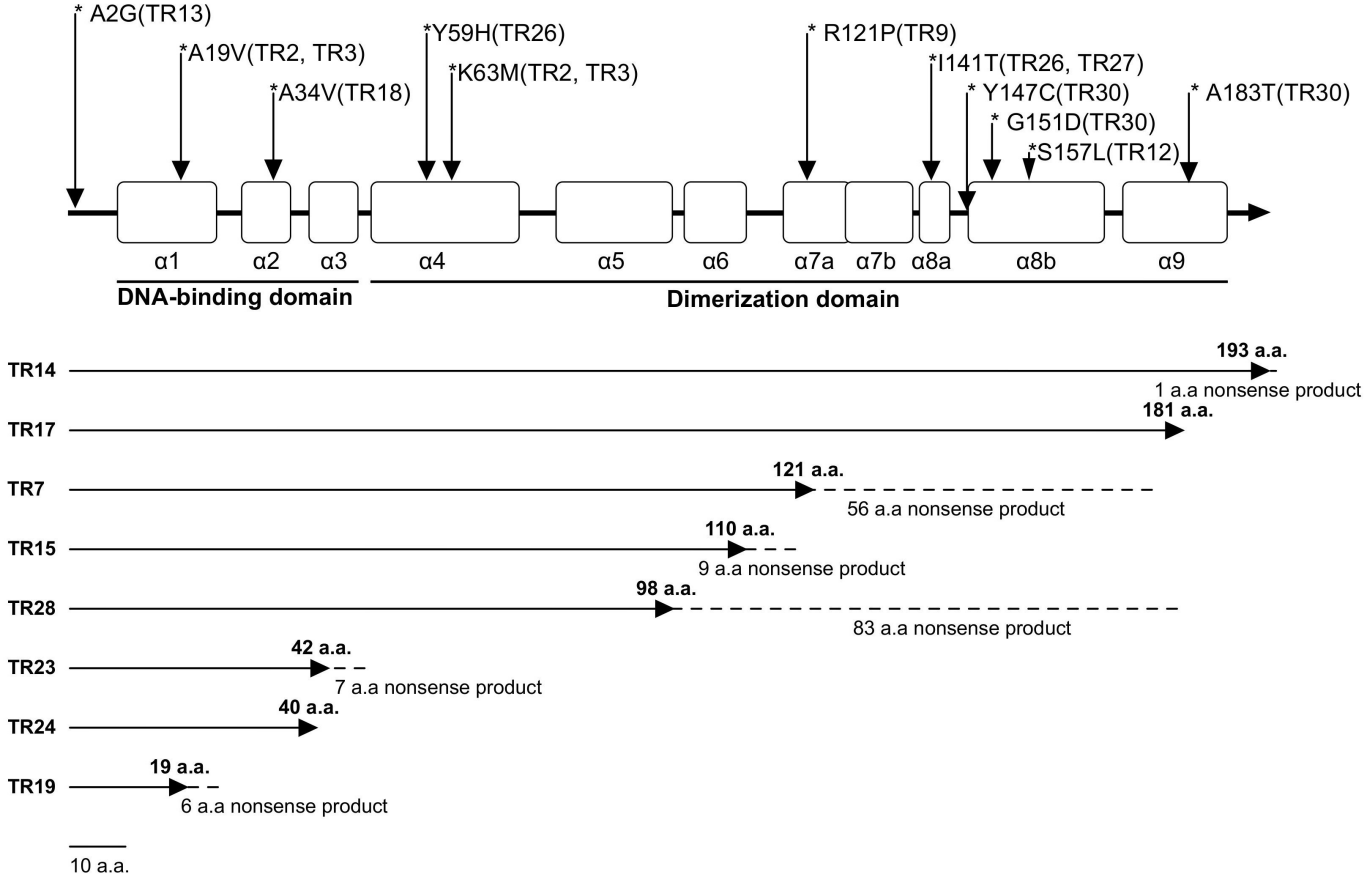
RamR protein mutations in TRKP clinical isolates. The open boxes indicate the nine α-helices of RamR (α1–α9). The reference sequence of K. pneumoniae MGH78578 (CP000647) was used to identify RamR mutations. Solid lines indicate efficiently transcribed RamR proteins and dashed lines indicate inefficiently transcribed RamR proteins.

In comparison with the standard WT strain MHG78578 (GenBank number CP000647), 22 isolates (22/30,73.3%) showed nucleotide changes in *ramR*. Five isolates (5/22, 22.7%) had a deletion of *ramR* gene, and 17 isolates (17/22, 77.3%) carried frameshift or substitution mutations in *ramR*, most of the substitutions exist in the DNA-binding domain. Among the more common *ramR* mutations are A19V substitution, K63M substitution, and I141T substitution (**Table 1**). Seven isolates produced truncated RamR proteins, including TR17 (181 amino acids [aa]), TR7 (121 aa), TR5 (110 aa), TR28 (98 aa), TR23 (42 aa), TR24 (40 aa) and TR19 (19 aa) (**Figure 1**).

The *tet(A)* variant is a major cause of tigecycline resistance: type 1 (n=17) and type 3 (n=2). Type 3 *tet(A)* showed a single amino acid difference from type 1 and a 28-bp nucleotide deletion.

The amino acid substitution involved in two *acrR* mutants (S215P, Y114F, V165I in TR6 and E200V in TR18) has not been previously reported. Of the three *oqxR* mutant isolates (E24R in TR1, C100Y in TR19, and 226-227 base deletion in TR29), E24R mutation in TR1 isolates did not lead to overexpression of *oqxB*. Recently, mutations in the *rpsj* gene encoding the ribosomal protein S10 have been reported to be associated with tigecycline resistance in Klebsiella pneumoniae. Although *rpsj* gene was present in all 30 TRKP isolates in our study, no *rpsj* gene mutation was found. *The tmexC* gene was detected in 2 TRKP isolates. The ST1561 TmexCD-ToprJ-positive TRKP strain did not use tigecycline during treatment, and eventually the patient recovered and was discharged successfully. Resistance gene screening revealed the presence of type 1 *tet(A)* mutants in this strain, along with A2G mutations in ramR, showing expression of *rpsj* and *oqxr* genes, but not *acrR.* In contrast, the ST661 strain was treated with tigecycline, but the patient sadly died. The strain expresses type 1 *tet(A)* mutant, *rpsj, ramR* and *acrR*, but does not express *oqxr*. Additionally, no mobilized tigecycline resistance genes, such as *tet(X)*, were detected.

### 3.4 Pump and regulator gene expression of TRKP isolates.

**Figure 2:**
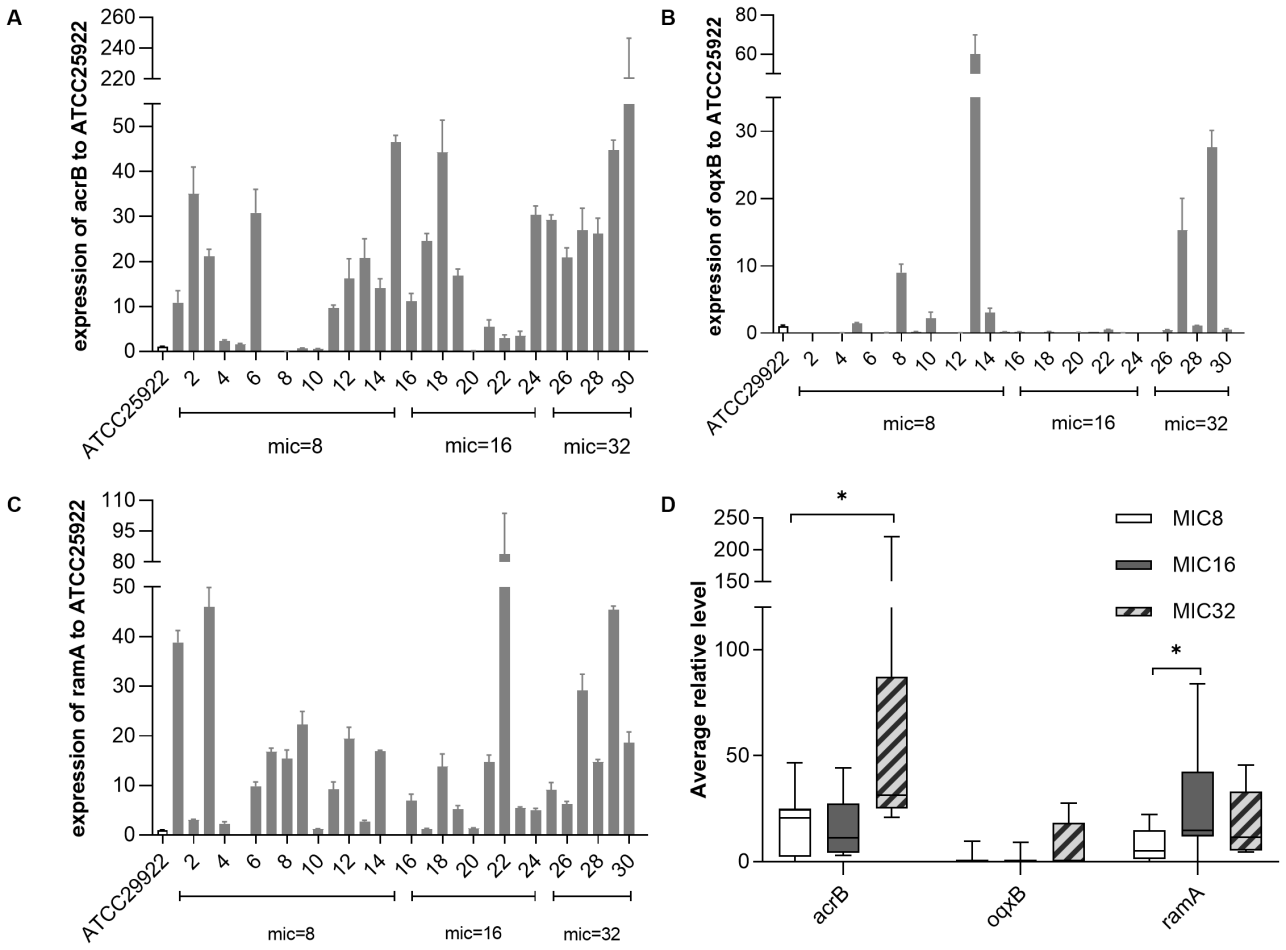
The expression levels of resistance-nodulation-cell division (RND) efflux pump gene and *ramA* gene in *Klebsiella pneumonia.* The expression levels of resistance-nodulation-cell division (RND) efflux pump gene and *ramA* gene in *K*. *pneumoniae* were determined by qPCR and the target gene expression levels were divided into three groups according to the MIC value of tigecycline (=8 mg/L, = 16mg/L, = 32mg/L) for comparison, with tigecycline-susceptible *Escherichia coli* ATCC 25922 as control (expression = 1). **(A)** Relative expression (RE) level of *acrB* in TRKP strains; **(B)** RE level of *oqxB* in TRKP strains; **(C)** RE level of *ramR* in TRKP strains; **(D)**Average RE of *acrB, oqxB*, and *ramA* in TRKP isolates treated with different tigecycline MIC values. The bars represent the average value and the error bars represent the standard error of the mean value. Data were analyzed by Mann–Whitney U test (*P <0.05; **P < 0.01; ***P < 0.001; ****P < 0.0001).

We evaluated expression levels of efflux pump AcrAB-TolC, OqxAB, and transcriptional regulatory gene *ramA*. The qRT-PCR analysis showed that 22 of the 30 Tigecycline-resistant strains had overexpression of *acrB* gene (5.49- to 48.49- fold) and *ramA* gene (5.20-83.93 fold). However, in the OqxAB efflux pump pathway, only 4 strains overexpressed the *oqxB* gene (9.02- to 60.07- fold) (**Figures 2A-C**). TRKP collected was divided into three groups according to MIC value (MIC=8ug/ml, MIC=16ug/ml, MIC=32ug/ml). Compared with the group with MIC of 8ug/ml, *acrB* was statistically significant when MIC was 32 groups(*p* 0.0302). There were significant differences in *ramA* when MIC was 16 (*p* 0.0248) (**Figure 2D**).

### 3.5 Molecular Epidemiology based on MLST

Twenty-five different STs were observed in 30 TRKP isolates. ST307 was the dominant strain, accounting for 4 strains (11.8%), followed by ST15 type, accounting for 2 strains (6.7%). No novel ST types were detected in this study.

## 4 Discussion

The majority of TRKP strains in our hospital come from older adults and those in the ICU with catheters. Severe pneumonia caused by TRKP often results in extended hospital stays (3-370 days) and poor outcomes, with a 36.7% mortality rate. *K. pneumoniae* comes from a variety of specimens and can cause infections of the lungs, urinary tract, tissues, bile, and ducts. Antimicrobial susceptibility tests show that TRKP isolates exhibit high resistance to most antibiotics. The increased MIC of tigecycline may be linked to the use of other antibiotics, which are also expelled via the AcrAB-TolC pump (Roy et al., 2013). In this study, 80% of patients had no prior exposure to tigecycline, confirming that resistance can occur without direct exposure. Efflux pump inhibitors (EPIs) down-regulate their expression by interfering with the combination of efflux pump-related proteins, blocking the energy supply effect, and inhibiting the substrate’s passage through efflux pump channels [16]. To detect the presence of overexpression of efflux pumps in TRKP strains, we used efflux pump inhibitor (EPI) PAβN to evaluate efflux pump activity. In our study, 96.6% of TRKP strains showed a fourfold or greater reduction when PAβN was present. The MIC of tigecycline remained unchanged when PAβN was present in TR23. We believe that the following factors may be involved: First, changes in membrane permeability, such as mutations of ompK35 and ompK36 genes, may lead to decreased permeability of the outer membrane to tigecycline; Second, in the presence of PAβN, other efflux pumps may still expel tigecycline. Finally, the metabolic status of bacteria may also affect their susceptibility to drugs. We plan to further explore these resistance mechanisms through whole genome sequencing. However, most of the existing efflux pump inhibitors have obvious toxicity, and how to develop clinical drugs with high specificity, low toxicity, and high safety remains to be further explored.

*Tet (A)* belongs to the MFS efflux pump family with mutations that allow tigecycline to accumulate within bacterial cells leading to resistance (Linkevicius et al., 2016). In 2017, Chiu et al. first discovered that type 1 *tet(A)* raised the MIC of tigecycline by a factor of 8. It was also demonstrated that in the case of *tet(A)* mutation, loss of *RamR* protein has a synergistic effect on tigecycline resistance in *K. pneumoniae* (Chiu et al., 2017). Five years later, Peng et al. demonstrated through cloning experiments that type 3 *tet (A)* could raise the MIC of tigecycline fourfold (Peng et al., 2022a). In our experiments, we found that almost all isolates carried *tet (A)* mutations or *ramR* mutations. No *ramR* was detected in five of the isolates, suggesting that their *ramR* gene may have been truncated or deleted, similar to the fully *ramR* deletion mutant of *Klebsiella pneumoniae* strain KPBj1 M3 Lev (Bialek-Davenet et al., 2013). Furthermore, the deletion and insertion of various fragments among the seven isolates contributed to the premature appearance of the stop codon. This may result in the loss of the α8-α9 region, disrupt dimerization, and ultimately lead to a loss of function (Yamasaki et al., 2013). Fortunately, A large study has shown that although *ramR* mutants can enhance bacterial resistance, they also induce an enhanced immune response by modulating the structure of lipid A, thereby reducing the pathogen’s ability to kill in organs and blood (Yu et al., 2024).

Point mutations in the local suppressor *oqxR* have been shown to cause the OqxAB efflux pump to become overactive, giving the isolates increased virulence and multidrug-resistant characteristics (Bialek-Davenet et al., 2015). Previous studies have shown that amino acid substitution mutations such as V102G and V130A confer resistance to tigecycline (Bialek-Davenet et al., 2015; Chiu et al., 2017). This paper identifies for the first time two additional amino acid substitutions (E24R and C100Y) and frameshift deletions involving amino acids 226 to 227, which may negatively impact function. Therefore, the effect of these mutations on drug resistance requires further investigation. The recent discovery of the novel tetracycline-inactivating enzyme *tet(X)* homologs and the efflux pump gene clusters Tmexd-toprj has been linked to high levels of tigecycline resistance. These resistance mechanisms can be horizontally transferred via mobile elements like plasmids, spreading to humans, animals, and the environment, and posing a significant public health threat (Guo et al., 2024; Pan et al., 2024). A report identified 237 bacterial strains worldwide carrying the *tmexCD-toprJ* gene, with 92.83% originating from China (Dong et al., 2022). These strains represent 50 unique sequence types. Our study marks the first identification of two novel tmexCD-toprJ-positive strains, ST661 and ST1561, offering fresh insights into microbial research and underscoring the significance of bacterial diversity alongside its vast research potential. Tmexcd-positive strains were previously found mainly on chromosomes, but the discovery of plasmids in the past five years suggests a possible transmission mechanism. Despite attempts to perform conjugation experiments to determine whether tmexC is located on the plasmid and its ability to transfer, they were unsuccessful, suggesting that they may indeed be located on chromosomes. To further test this hypothesis, whole genome sequencing is planned to confirm the specific location and function of tmexCD-toprJ. In addition, patients infected with ST1561 were observed to survive, while those infected with ST661 died, a difference that may reflect significant differences in the pathogenicity and virulence of the strains. *rpsj* mutations linked to tigecycline resistance are generally found in the amino acids located between positions 53 and 60 of the S10 ribosomal protein (Beabout et al., 2015). Fortunately, we did not find any *rpsj* mutations related to tigecycline in the strains, nor did we detect any cases of *tet(X)* carrying.

Both AcrAB and OqxAB efflux pumps are common resistance mechanisms in enterobacteria (Liu et al., 2018). Research shows that OqxAB overexpression can lead to resistance to several antimicrobials, including chloramphenicol, quinolones, furantoin, and tigecycline (Li et al., 2019). In our study, *acrB* expression increased with varying MIC levels, while *oqxB* was overexpressed in only 4 bacterial strains. This aligns with findings by Perez et al., showing that while OqxAB is widely distributed in *K. pneumoniae*, it is not always overexpressed (Perez et al., 2013). It is important to note that the *ramR* gene was absent from the highly resistant strain TR4 (MIC 8µg/ml), and efflux pump inhibition tests demonstrated that the resistance mechanism was linked to the efflux pump even though acrB expression was only 2.32 times higher. Furthermore, no known resistance determinants were discovered, indicating that resistance may emerge through other pathways such as KpgABC or MacAB-TolC efflux pumps. Mutation of *ramR*, as an inhibitor of *ramA*, leads to up-regulation of *ramA* expression, which in turn increases the expression of acrAB or oqxAB efflux pump. In this study, three isolates (TR10, TR16, TR25) showed elevated *ramA* expression without any *ramR* mutations. This may be linked to mutations in the RamR recognition sites (PI and PII promoters) or Lon protease mutations (Rosenblum et al., 2011; Ricci et al., 2014), requiring further investigation to clarify this finding. Although it has been established that efflux mechanisms generally only lead to low levels of resistance to tigecycline, we speculate that multiple mechanisms may be at work at the same time, with possible synergistic effects. Therefore, more in-depth research is urgently needed to explore the interrelationships and effects of these mechanisms.

Additionally, 30 K*. pneumoniae* isolates were classified into 25 distinct ST types, reflecting the significant genetic diversity of TRKP. Previous research has indicated that most TRKP strains are resistant to multiple antibiotics and carry virulence factors, heightening the risk of resistance and virulence gene transfer. Monitoring the spread of these clones is essential to prevent their emergence as clinical pathogens (Peng et al., 2022a).

This study’s limitation is its single-center scope, which may limit representativeness. Additionally, only certain resistance genes were screened, leaving other mechanisms unexplored. Future efforts will expand sample size through multi-center studies and broaden genetic screening to better understand resistance mechanisms and enhance the study’s clinical relevance. In conclusion, *acrB* overexpression and *tet(A)* mutations are key contributors to tigecycline resistance in *K. pneumoniae* in southwest China.

## Data Availability

The original contributions presented in the study are included in the article/**Supplementary Material**. Further inquiries can be directed to the corresponding author.
