# Supplementary material for: The investigation of molecular epidemiological characteristics and resistance mechanism of tigecycline resistant Klebsiella pneumoniae from a large teaching hospital in southwest China, Chongqing
Source: Front Cell Infect Microbiol. 2025 Mar 13;15:1540967. doi: 10.3389/fcimb.2025.1540967 (PMC11965929; doi:10.3389/fcimb.2025.1540967)
Supplement: Supplementary file 1 [file Table1.docx]

**Supplement Table S1**. The primers used to detect the resistance gene

| Purpose | Gene | Forward sequence (5′→3′) | Reverse sequence (5′→3′) | Reference |
| --- | --- | --- | --- | --- |
| Mutation detection | *ramR* | CACGGTTCATATCCTGACCA | CCRTCGACCTTAAACACGTC | (Chiu et al., 2017)  (Hirabayashi et al., 2021) |
|  | *acrR* | GCTAAGCTGCCTGAGAGCAT | ATGCAAATGCCGGAGAATAC |  |
|  | *rpsJ* | ACAGCCGGTTCGATATGA | AGTAACGCGGTTTGCTTC |  |
|  | *oqxR* | GTCACCAGAAAATGATTAATGCGC | GCCTTTGCCCGTGAAATCAG |  |
|  | *tet(A)* | GCCTTTCCTTTGGGTTCTCT | TGTCCGACAAGTTGCATGAT |  |
|  | *tet(X)* | CCCGAAAATCGWTTTGACAATCC TG | GTTTCTTCAACTTSCGTGTC GGTAAC |  |
|  | *tmexC* | TGGCGGGGATCGTGCTCAAGCGC AC | CAGCGTGCCCTTGCKCTCGATATCG |  |
|  | *tet(L)* | CATTTGGTCTTATTGGATCG | ATTACACTTCCGATTTCGG | (Du et al., 2018) |
| qRT-PCR | *ramA* | GATATCGCTCGCCATGC | CTGTGGTTCTCTTTGCGGTA G | (Wang et al., 2015)  (He et al., 2015) |
|  | *acrB* | AAACTTCGCCACTACGTCATA | AGCTTAACGCCTCGATCAT |  |
|  | *oqxB* | CGAAGAAAGACCTCCCTACCC | CGCCGCCAATGAGATACA |  |
|  | *rpoB* | CGCGTATGTCCGATCGAAA | GCGTCTCAAGGAAGCCATAT TC |  |
| MLST | rpoB | GGCGAAATGGCWGAGAACCA | GAGTCTTCGAAGTTGTAACC | Online |
|  | gapA | TGAAATATGACTCCACTCACGG | CTTCAGAAGCGGCTTTGATGGCTT |  |
|  | mdh | CCCAACTCGCTTCAGGTTCAG | CCGTTTTTCCCCAGCAGCAG |  |
|  | pgi | GAGAAAAACCTGCCTGTACTGCTGGC | CGCGCCACGCTTTATAGCGGTTAAT |  |
|  | phoE | ACCTACCGCAACACCGACTTCTTCGG | TGATCAGAACTGGTAGGTGAT |  |
|  | infB | CTCGCTGCTGGACTATATTCG | CGCTTTCAGCTCAAGAACTTC |  |
|  | tonB | CTTTATACCTCGGTACATCAGGTT | ATTCGCCGGCTGRGCRGAGAG |  |

qRT-PCR, quantitative real-time PCR

**Supplement Table 2.** Antibacterial profiles of 13 kinds of antibacterial agents by TRKP.

| Isolate | use tigecycline |  |  |  |  |  |  | MIC^a^(mg/L) |  |  |  |  |  |  |
| --- | --- | --- | --- | --- | --- | --- | --- | --- | --- | --- | --- | --- | --- | --- |
|  |  | IMP | CRO | CFZ | FEP | ATM | CAZ | AMK | LEV | CIP | GEN | CST | MH | TGC |
| TR1 | N | ≤0.25, S | ≥64, R | ≥64, R | 32, R | ≥32, R | ≥64, R | ≥64, R | ≥8, R | ≥4, R | 16, R | ≤16, S | 16, R | 8, R |
| TR2 | N | ≤0.25, S | ≥64, R | ≥64, R | 32, R | ≥32, R | ≥64, R | ≥64, R | ≥8, R | ≥4, R | 16, R | ≤16, S | 16, R | 8, R |
| TR3 | N | ≤0.25, S | ≥64, R | 1, S | 2, S | 4, S | 32, R | ≤16, S | ≥8, R | ≥4, R | 4, S | ≥64, R | 16, R | 8, R |
| TR4 | N | ≤0.25, S | ≥64, R | ≤0.25, S | ≤1, S | 2, S | 0.5, S | ≤16, S | 1, S | 1, S | 2, S | 32, I | 16, R | 8, R |
| TR5 | N | 1, S | ≥64, R | ≤0.25, S | ≤1, S | 1, S | 0.5, S | ≤16, S | ≥8, R | ≥4, R | 2, S | ≤16, S | 16, R | 8, R |
| TR6 | Y | 1, S | ≤0.25, S | ≤0.25, S | ≤1, S | 1, S | 0.5, S | ≤16, S | ≥8, R | ≥4, R | 2, S | ≤16, S | 16, R | 8, R |
| TR7 | Y | ≤0.25, S | ≥64, R | ≥64, R | 16, R | ≥32, R | ≥64, R | ≤16, S | 1, S | ≥4, R | 4, S | ≤16, S | 16, R | 8, R |
| TR8 | N | ≤0.25, S | ≥64, R | 1, S | 16, R | 2, S | 0.5, S | ≤16, S | ≥8, R | ≥4, R | 2, S | ≤16, S | 16, R | 8, R |
| TR9 | N | 0.5, S | ≥64, R | ≥64, R | 16, R | 16, I | 4, S | ≤16, S | 1, S | ≥4, R | 4, S | ≥64, R | 16, R | 8, R |
| TR10 | N | ≤0.25, S | ≥64, R | ≥64, R | 16, R | ≥32, R | ≥64, R | ≤16, S | ≥8, R | ≥4, R | 4, S | ≤16, S | 16, R | 8, R |
| TR11 | N | ≤0.25, S | ≥64, R | ≥64, R | 16, R | 8, S | 32, R | ≤16, S | ≥8, R | ≥4, R | 16, R | ≤16, S | 16, R | 8, R |
| TR12 | N | ≤0.25, S | ≥64, R | ≥64, R | 32, R | ≥32, R | ≥64, R | ≤16, S | ≥8, R | ≥4, R | 4, S | ≤16, S | 16, R | 8, R |
| TR13 | N | 1, S | ≤0.25, S | ≥64, R | ≥64, R | ≥32, R | ≥64, R | ≤16, S | ≥8, R | ≥4, R | 32, R | ≥64, R | 16, R | 8, R |
| TR14 | N | ≤0.25, S | ≥64, R | ≥64, R | 32, R | 8, S | 32, R | ≤16, S | 4, I | 2, I | 32, R | ≤16, S | 16, R | 8, R |
| TR15 | N | 0.5, S | ≥64, R | ≤0.25, S | 2, S | 2, S | 8, I | ≤16, S | 1, S | 1, S | 4, S | ≥64, R | 16, R | 8, R |
| TR16 | Y | ≤0.25, S | ≤0.25, S | ≥64, R | 32, R | ≥32, R | 8, I | ≤16, S | ≥8, R | ≥4, R | 4, S | ≥64, R | 32, R | 16, R |
| TR17 | N | ≤0.25, S | ≥64, R | ≤0.25, S | ≤1, S | 2, S | 0.5, S | ≤16, S | ≥8, R | ≥4, R | 2, S | 32 | 32, R | 16, R |
| TR18 | N | ≤0.25, S | ≤0.25, S | ≥64, R | 32, R | ≥32, R | 8, I | ≤16, S | ≥8, R | ≥4, R | 4, S | ≥64, R | 32, R | 16, R |
| TR19 | N | ≤0.25, S | ≥64, R | ≤0.25, S | ≤1, S | 2, S | 0.5, S | ≤16, S | ≥8, R | ≥4, R | 2, S | 32 | 32, R | 16, R |
| TR20 | N | ≤0.25, S | ≥64, R | ≥64, R | 16, R | 16, I | 8, I | ≥64, R | 4, I | ≥4, R | 4, S | ≥64, R | 8, R | 16, R |
| TR21 | Y | ≤0.25, S | ≥64, R | ≥64, R | ≥64, R | ≥32, R | ≥64, R | ≤16, S | ≥8, R | ≥4, R | 4, S | ≥64, R | 32, R | 16, R |
| TR22 | Y | ≤0.25, S | ≥64, R | ≥64, R | 32, R | ≥32, R | ≥64, R | ≤16, S | ≥8, R | ≥4, R | 4, S | ≤16, S | 16, R | 16, R |
| TR23 | N | ≥16, S | ≤0.25, S | ≥64, R | ≥64, R | ≥32, R | ≥64, R | ≥64, R | ≥8, R | ≥4, R | 4, S | ≥64, R | 16, R | 16, R |
| TR24 | N | ≤0.25, S | ≤0.25, S | ≥64, R | 16, R | 8, S | 2, S | ≤16, S | ≥8, R | ≥4, R | 4, S | ≥64, R | 16, R | 16, R |
| TR25 | N | 0.5, S | ≤0.25, S | ≥64, R | ≥64, R | ≥32, R | 0.5, S | ≤16, S | ≥8, R | ≥4, R | 4, S | ≤16, S | 64, R | 32, R |
| TR26 | N | 0.5, S | ≤0.25, S | ≥64, R | 2, S | ≥32, R | 8, I | ≤16, S | ≥8, R | ≥4, R | 4, S | ≤16, S | 64, R | 32, R |
| TR27 | Y | ≤0.25, S | ≤0.25, S | ≥64, R | 16, R | 8, S | 4, S | ≤16, S | ≥8, R | ≥4, R | 4, S | ≤16, S | 16, R | 32, R |
| TR28 | N | ≤0.25, S | ≤0.25, S | ≤0.25, S | 2, S | 2, S | 0.5, S | ≤16, S | 1, S | ≥4, R | 4, S | ≥64, R | 16, R | 32, R |
| TR29 | N | 0.5, S | ≤0.25, S | ≤0.25, S | 2, S | 2, S | 2, S | ≤16, S | 1, S | 1, S | 4, S | ≤16, S | 64, R | 32, R |
| TR30 | N | ≤0.25, S | ≤0.25, S | ≤0.25, S | 2, S | 2, S | 2, S | ≤16, S | 1, S | 1, S | 4, S | ≤16, S | 16, R | 32, R |

^a^ IMP, imipenem; CRO, ceftriaxone; CFZ, cefoperazone; FEP, cefepime; ATM, amtronam; CAZ, ceftazidime; AMK, Amikacin; LEV, Levofloxacin; CIP, Ciprofloxacin; GEN, gentamicin; CST, cefoxitin; MH, Minocycline; TGC, Tigecycline. Y, yes; N, no.

**Supplement Figure 1.** Amino acid difference between mutant Tet (A) and wild Tet (A)

As reported by Chiu et al, wild type tet(A) gene sequence (GenBank entry number: X00006) and Type1 tet(A) gene sequence (GenbanX61367) were obtained(Chiu et al., 2017). As reported by Peng et al., Type3 tet(A) gene sequence (GenbanJAKZNN000000000.1)(Peng et al., 2022).

**Supplement Figure 2.** Amino acid difference between mutant AcrR and wild AcrR.

The reference sequence of *K. pneumoniae* MGH78578 (CP000647) was used to identify AcrR mutations(Chiu et al., 2017).

**Supplement Figure 3.** Amino acid difference between mutant OqxR and wild OqxR.

The reference sequence of *K. pneumoniae* MGH78578 (CP000647) was used to identify OqxR mutations(Chiu et al., 2017).

**Supplement Figure 4.** Amino acid difference between mutant RamR and wild RamR.

The reference sequence of *K. pneumoniae* MGH78578 (CP000647) was used to identify RamR mutations(Chiu et al., 2017).

Chiu, S.K., Huang, L.Y., Chen, H., Tsai, Y.K., Liou, C.H., Lin, J.C., et al. (2017). Roles of ramR and tet(A) Mutations in Conferring Tigecycline Resistance in Carbapenem-Resistant Klebsiella pneumoniae Clinical Isolates. *Antimicrob Agents Chemother* 61(8). doi: 10.1128/aac.00391-17.

Du, X., He, F., Shi, Q., Zhao, F., Xu, J., Fu, Y., et al. (2018). The Rapid Emergence of Tigecycline Resistance in bla(KPC-2) Harboring Klebsiella pneumoniae, as Mediated in Vivo by Mutation in tetA During Tigecycline Treatment. *Front Microbiol* 9**,** 648. doi: 10.3389/fmicb.2018.00648.

He, F., Fu, Y., Chen, Q., Ruan, Z., Hua, X., Zhou, H., et al. (2015). Tigecycline susceptibility and the role of efflux pumps in tigecycline resistance in KPC-producing Klebsiella pneumoniae. *PLoS One* 10(3)**,** e0119064. doi: 10.1371/journal.pone.0119064.

Hirabayashi, A., Dao, T.D., Takemura, T., Hasebe, F., Trang, L.T., Thanh, N.H., et al. (2021). A Transferable IncC-IncX3 Hybrid Plasmid Cocarrying bla(NDM-4), tet(X), and tmexCD3-toprJ3 Confers Resistance to Carbapenem and Tigecycline. *mSphere* 6(4)**,** e0059221. doi: 10.1128/mSphere.00592-21.

Peng, K., Wang, Q., Li, Y., Wang, M., Kurekci, C., Li, R., et al. (2022). Molecular mechanisms and genomic basis of tigecycline-resistant Enterobacterales from swine slaughterhouses. *Microbiol Res* 264**,** 127151. doi: 10.1016/j.micres.2022.127151.

Wang, X., Chen, H., Zhang, Y., Wang, Q., Zhao, C., Li, H., et al. (2015). Genetic characterisation of clinical Klebsiella pneumoniae isolates with reduced susceptibility to tigecycline: Role of the global regulator RamA and its local repressor RamR. *Int J Antimicrob Agents* 45(6)**,** 635-640. doi: 10.1016/j.ijantimicag.2014.12.022.

Raw data:

>acrR-1

Ttaagctgacaagctctccgggccgcgtagcgtcgggcagaattgatacatctccagcagaatagcgacgtagtcccgcgcttccgcatgcaggtcgaacgaatcgggggcaaacagccagttttccatcagcccggaaaggtagctgcgcattaacacggccgcccgccgggtgagtaaattggcgggcagcagcttcgccgcgatgcactctttcaaggtctgctcgatacgctcataactcgccagggagagctgccgctgggcctgctgcaccacggtcatttcaccgacgaactcacacttatgatagataatctccatcattaatcgtcgacgttcttctgtcactgtcgcttcaagaacatagactagaatctccctgataactgagagtggatcgttggggaattttgcccgatactcaatttcgagatcgctaatactggcgtctgacagctcccaaatttcgttgaataaatctgatttattcttgaaatgccagtagatagcccccctcgttacacccgcagcttttgcaattgttgccaacgaggtagatgatacgccttgctgcgaaaacagacgcagagcaacatccagaatcagttgccgggtttcacgtgcctgttgtttggtttttcgtgccat

>acrR-2

GGCGCCGCGCGCAGATAGCACTTCTCAGCGCTGGCGAATAGCGGCGGACGGCGCTAAAAATTCACGCCCATCCGCGCGCTACCTTTAAGCTGACAAGCTCTCCGGGCCGCGTAGCGTCGGGCAGAATTGATACATCTCCAGCAGAATAGCGACGTAGTCCCGCGCTTCCGCATGCAGGTCGAACGAATCGGGGGCAAACAGCCAGTTTTCCATCAGCCCGGAAAGGTAGCTGCGCATTAACACGGCCGCCCGCCGGGTGAGTAAATTGGCGGGCAGCAGCTTCGCCGCGATGCACTCTTTCAAGGTCTGCTCGATACGCTCATAACTCGCCAGGGAGAGCTGCCGCTGGGCCTGCTGCACCACGGTCATTTCACCGACGAACTCACACTTATGATAGATAATCTCCATCATTAATCGTCGACGTTCTTCTGTCACTGTCGCTTCAAGAACATAGACTAGAATCTCCCTGATAACTGAGAGTGGATCGTTGGGGAATTTTGCCCGATACTCAATTTCGAGATCGCTAATACTGGCGTCTGACAGCTCCCAAATTTCGTTGAATAAATCTGATTTATTCTTGAAATGCCAGTAGATAGCCCCCCTCGTTACACCCGCAGCTTTTGCAATTGTTGCCAACGAGGTAGATGATACGCCTTGCTGCGAAAACAGACGCAGAGCAACATCCAGAATCAGTTGCCGGGTTTCACGTGCCTGTTGTTTGGTTTTTCGTGCCATAGGTTAATGACTTTACAGAGGTTACGTTTACATACATTTGTGAATGTATGTACCATAGCATGACCATAATAGAAAGACTGTAGTGGGTTTGTGGTTGTTTGAGCCACTGAACATTTTGAAATTGGACACTCGAGGTTTACATATGAACAAAAACAGAGGGTTAACGCCTCTGGCGTCGTCTGTGTCTCCCCCGG

>acrR-3 GGCTGCGCGCGCAGATAGCACTTCTCAGCGCTGGCGAATAGCGGCGGACGGCGCTAAAAATTCACGCCCATCCGCGCGCTACCTTTAAGCTGACAAGCTCTCCGGGCCGCGTAGCGTCGGGCAGAATTGATACATCTCCAGCAGAATAGCGACGTAGTCCCGCGCTTCCGCATGCAGGTCGAACGAATCGGGGGCAAACAGCCAGTTTTCCATCAGCCCGGAAAGGTAGCTGCGCATTAACACGGCCGCCCGCCGGGTGAGTAAATTGGCGGGCAGCAGCTTCGCCGCGATGCATTCTTTCAAGGTCTGCTCGATACGCTCATAACTCGCCAGGGAGAGCTGCCGCTGGGCCTGCTGCACCACGGTCATTTCACCGACGAACTCACACTTATGATAGATAATCTCCATCATTAATCGTCGACGTTCTTCTGTCACTGTCGCTTCAAGAACATAGACTAGAATCTCCCTGATAACTGAGAGTGGATCGTTGGGGAATTTTGCCCGATACTCAATTTCGAGATCGCTAATACTGGCGTCTGACAGCTCCCAAATTTCGTTGAATAAATCTGATTTATTCTTGAAATGCCAGTAGATAGCCCCCCTCGTTACACCCGCAGCTTTTGCAATTGTTGCCAACGAGGTAGATGATACGCCTTGCTGCGAAAACAGACGCAGAGCAACATCCAGAATCAGTTGCCGGGTTTCACGTGCCTGTTGTTTGGTTTTTCGTGCCATAGGTTAATGACTTTACAGAGGTTACGTTTACATACATTTGTGAATGTATGTACCATAGCATGACCATAATAGAAAGACTGTAGTGGGTTTGTGGTTGTTTGAGCCACTGAACATTTTGAAATTGGACACTCGAGGTTTACATATGAACAAAAACAGAGGGTTAACGCCTCTGGCGGTCGTCTGAGTTCTCTCGGG

>acrR-4 GGGCTGCGCGCGCAGATAGCACTTCTCAGCGCTGGCGAATAGCGGCGGACGGCGCTAAAAATTCACGCCCATCCGCGCGCTACCTTTAAGCTGACAAGCTCTCCGGGCCGCGTAGCGTCGGGCAGAATTGATACATCTCCAGCAGAATAGCGACGTAGTCCCGCGCTTCCGCATGCAGGTCGAACGAATCGGGGGCAAACAGCCAGTTTTCCATCAGCCCGGAAAGGTAGCTGCGCATTAACACGGCCGCCCGCCGGGTGAGTAAATTGGCGGGCAGCAGCTTCGCCGCGATGCACTCTTTCAAGGTCTGCTCGATACGCTCATAACTCGCCAGGGAGAGCTGCCGCTGGGCCTGCTGCACCACGGTCATTTCACCGACGAACTCACACTTATGATAGATAATCTCCATCATTAATCGTCGACGTTCTTCTGTCACTGTCGCTTCAAGAACATAGACTAGAATCTCCCTGATAACTGAGAGTGGATCGTTGGGGAATTTTGCCCGATACTCAATTTCGAGATCGCTAATACTGGCGTCTGACAGCTCCCAAATTTCGTTGAATAAATCTGATTTATTCTTGAAATGCCAGTAGATAGCCCCCCTCGTTACACCCGCAGCTTTTGCAATTGTTGCCAACGAGGTAGATGATACGCCTTGCTGCGAAAACAGACGCAGAGCAACATCCAGAATCAGTTGCCGGGTTTCACGTGCCTGTTGTTTGGTTTTTCGTGCCATAGGTTAATGACTTTACAGAGGTTACGTTTACATACATTTGTGAATGTATGTACCATAGCATGACCATAATAGAAAGACTGTAGTGGGTTTGTGGTTGTTTGAGCCACTGAACATTTTGAAATTGGACACTCGAGGTTTACATATGAACAAAAACAGAGGGTTAACGCCTCTGGCGGTCGTCTGAGGTTCTTCGG

>acrR-5 GGGGCTGCGCGCGCAGATAGCACTTCTCAGCGCTGGCGAATAGCGGCGGACGGCGCTAAAAATTCACGCCCATCCGCGCGCTACCTTTAAGCTGACAAGCTCTCCGGGCCGCGTAGCGTCGGGCAGAATTGATACATCTCCAGCAGAATAGCGACGTAGTCCCGCGCTTCCGCATGCAGGTCGAACGAATCGGGGGCAAACAGCCAGTTTTCCATCAGCCCGGAAAGGTAGCTGCGCATTAACACGGCCGCCCGCCGGGTGAGTAAATTGGCGGGCAGCAGCTTCGCCGCGATGCACTCTTTCAAGGTCTGCTCGATACGCTCATAACTCGCCAGGGAGAGCTGCCGCTGGGCCTGCTGCACCACGGTCATTTCACCGACGAACTCACACTTATGATAGATAATCTCCATCATTAATCGTCGACGTTCTTCTGTCACTGTCGCTTCAAGAACATAGACTAGAATCTCCCTGATAACTGAGAGTGGATCGTTGGGGAATTTTGCCCGATACTCAATTTCGAGATCGCTAATACTGGCGTCTGACAGCTCCCAAATTTCGTTGAATAAATCTGATTTATTCTTGAAATGCCAGTAGATAGCCCCCCTCGTTACACCCGCAGCTTTTGCAATTGTTGCCAACGAGGTAGATGATACGCCTTGCTGCGAAAACAGACGCAGAGCAACATCCAGAATCAGTTGCCGGGTTTCACGTGCCTGTTGTTTGGTTTTTCGTGCCATAGGTTAATGACTTTACAGAGGTTACGTTTACATACATTTGTGAATGTATGTACCATAGCATGACCATAATAGAAAGACTGTAGTGGGTTTGTGGTTGTTTGAGCCACTGAACATTTTGAAATTGGACACTCGAGGTTTACTTATGAACAAAACCGAGGGTTAACGCCTCTGGCGTCGTCTGTGTCTTCC

>acrR6

GGCGTGTAGCGCGCAGATAGCACTTCTCATCGCTGGCGAATAGCGGCGGACCGCGCTAAAAATTCACGCTCATCCGCACGCTTCGTTAAGCTGGCAGGCTCTCCGGGCCGCGAAGCGTGGGGCAGAATTGATACATCTCCAGCAGGATGGCGACGTAGTCACGCGCTTCCGCATGCAGGTCGAACGAGTCGGGAGCAAACAGCCAGTTCTCCATCAGGCCTGAAAGGTAGCTGCGCATTAAAATCGCCGCTCGCCGGGTGAGTAAATTGGCAGGCAACAGCTTCGCCGCGATACACTCTTTTAAGGTTTGCTCGATACGCTCGTAGCTCGCCAGGGAGAGCTGCCGCTGGGCCTGCTGCACTACGGTCATTTCTCCGACGAATTCGCACTTATGGAAGATAATCTCCATCATCAACCGACGACGCTCTTCTGTCACTGTCGCTTCAAGAACATAGACTAATATCTCCCTGATAACTGAGAGTGGATCGTTGGGGAATTTTGCCCGATACTCAATTTCGAGATCGCTAATACTGGCGTCTGACAGCTCCCAAATTTCGTTGAATAAATCTGATTTATTCTTGAAATGCCAGTAGATAGCCCCCCTCGTTACACCCGCAGCTTTTGCAATTGTTGCCAACGAGGTAGATGATACGCCTTGCTGCGAAAACAGACGCAGAGCAACATCCAGAATCAGTTGCCGGGTTTCACGTGCCTGTTGTTTGGTTTTTCGTGCCATAGGTTAATGACTTTACAGAGGTTACGTTTACATACATTTGTGAATGTATGTACCATAGCACGACCATAATAGAAAGACTGTAGTGGGTTTGTGGTTGTTTGAGCCACTGAACATTTTGAAATTGGACACTCGAGGTTTACATATGAACAAAAACAGAGGGTTAACGCCTCTGGGCGTCGTCTGAGTTCTCTCC

>acrR7

CGTTCCAGCGCGCGATAGCACTTCTCAGCGCTGGCGAATAGCGGCGGACGGCGCTAAAAATTCACGCCCATCCGCGCGCTACCTTTAAGCTGACAAGCTCTCCGGGCCGCGTAGCGTCGGGCAGAATTGATACATCTCCAGCAGAATAGCGACGTAGTCCCGCGCTTCCGCATGCAGGTCGAACGAATCGGGGGCAAACAGCCAGTTTTCCATCAGCCCGGAAAGGTAGCTGCGCATTAACACGGCCGCCCGCCGGGTGAGTAAATTGGCGGGCAGCAGCTTCGCCGCGATGCACTCTTTCAAGGTCTGCTCGATACGCTCATAACTCGCCAGGGAGAGCTGCCGCTGGGCCTGCTGCACCACGGTCATTTCACCGACGAACTCACACTTATGATAGATAATCTCCATCATTAATCGTCGACGTTCTTCTGTCACTGTCGCTTCAAGAACATAGACTAGAATCTCCCTGATAACTGAGAGTGGATCGTTGGGGAATTTTGCCCGATACTCAATTTCGAGATCGCTAATACTGGCGTCTGACAGCTCCCAAATTTCGTTGAATAAATCTGATTTATTCTTGAAATGCCAGTAGATAGCCCCCCTCGTTACACCCGCAGCTTTTGCAATTGTTGCCAACGAGGTAGATGATACGCCTTGCTGCGAAAACAGACGCAGAGCAACATCCAGAATCAGTTGCCGGGTTTCACGTGCCTGTTGTTTGGTTTTTCGTGCCATAGGTTAATGACTTTACAGAGGTTACGTTTACATACATTTGTGAATGTATGTACCATAGCATGACCATAATAGAAAGACTGTAGTGGGTTTGTGGTTGTTTGAGCCACTGAACATTTTGAAATTGGACACTCGAGGTTTACATATGAACAAAAACAGAGGGTTAACGCCTCTGGCGGTCGTTCTGATGTTCCTCAGGCAACTTAA

>acrR-8

GGGCTGCGCGCGCGATAGCACTTCTCAGCGCTGGCGAATAGCGGCGGACGGCGCTAAAAATTCACGCCCATCCGCGCGCTACCTTTAAGCTGACAAGCTCTCCGGGCCGCGTAGCGTCGGGCAGAATTGATACATCTCCAGCAGAATAGCGACGTAGTCCCGCGCTTCCGCATGCAGGTCGAACGAATCGGGGGCAAACAGCCAGTTTTCCATCAGCCCGGAAAGGTAGCTGCGCATTAACACGGCCGCCCGCCGGGTGAGTAAATTGGCGGGCAGCAGCTTCGCCGCGATGCACTCTTTCAAGGTCTGCTCGATACGCTCATAACTCGCCAGGGAGAGCTGCCGCTGGGCCTaGCTGCACCACGGTCATTTCACCGACGAACTCACACTTATGATAGATAATCTCCATCATTAATCGTCGACGTTCTTCTGTCACTGTCGCTTCAAGAACATAGACTAGAATCTCCCTGATAACTGAGAGTGGATCGTTGGGGAATTTTGCCCGATACTCAATTTCGAGATCGCTAATACTGGCGTCTGACAGCTCCCAAATTTCGTTGAATAAATCTGATTTATTCTTGAAATGCCAGTAGATAGCCCCCCTCGTTACACCCGCAGCTTTTGCAATTGTTGCCAACGAGGTAGATGATACGCCTTGCTGCGAAAACAGACGCAGAGCAACATCCAGAATCAGTTGCCGGGTTTCACGTGCCTGTTGTTTGGTTTTTCGTGCCATAGGTTAATGACTTTACAGAGGTTACGTTTACATACATTTGTGAATGTATGTACCATAGCATGACCATAATAGAAAGACTGTAGTGGGTTTGTGGTTGTTTGAGCCACTGAACATTTTGAAATTGGACACTCGAGGTTTACATATGAACAAAAACAGAGGGTTAACGCCTCTGGGGTCGTCTGTGTCTCTCCCAGAGGTATATACACAAAGGTTGCC

>acrR-9 CCCGCGCGCGCAGATAGCACTTCTCAGCGCTGGCGAATAGCGGCGGACGGCGCTAAAAATTCACGCCCATCCGCGCGCTACCTTTAAGCTGACAAGCTCTCCGGGCCGCGTAGCGTCGGGCAGAATTGATACATCTCCAGCAGAATAGCGACGTAGTCCCGCGCTTCCGCATGCAGGTCGAACGAATCGGGGGCAAACAGCCAGTTTTCCATCAGCCCGGAAAGGTAGCTGCGCATTAACACGGCCGCCCGCCGGGTGAGTAAATTGGCGGGCAGCAGCTTCGCCGCGATGCACTCTTTCAAGGTCTGCTCGATACGCTCATAACTCGCCAGGGAGAGCTGCCGCTGGGCCTGCTGCACCACGGTCATTTCACCGACGAACTCACACTTATGATAGATAATCTCCATCATTAATCGTCGACGTTCTTCTGTCACTGTCGCTTCAAGAACATAGACTAGAATCTCCCTGATAACTGAGAGTGGATCGTTGGGGAATTTTGCCCGATACTCAATTTCGAGATCGCTAATACTGGCGTCTGACAGCTCCCAAATTTCGTTGAATAAATCTGATTTATTCTTGAAATGCCAGTAGATAGCCCCCCTCGTTACACCCGCAGCTTTTGCAATTGTTGCCAACGAGGTAGATGATACGCCTTGCTGCGAAAACAGACGCAGAGCAACATCCAGAATCAGTTGCCGGGTTTCACGTGCCTGTTGTTTGGTTTTTCGTGCCATAGGTTAATGACTTTACAGAGGTTACGTTTACATACATTTGTGAATGTATGTACCATAGCATGACCATAATAGAAAGACTGTAGTGGGTTTGTGGTTGTTTGAGCCACTGAACATTTTGAAATTGGACACTCGAGGTTTACATATGAACAAAAACAGAGGGTTAACGCCTCTGGCGGTCGTCTGAGTTCTCTCCG

>acrR-10 GCCTGAGCGCGCAGATAGCACTTCTCAGCGCTGGCGAATAGCGGCGGACGGCGCTAAAAATTCACGCCCATCCGCGCGCTACCTTTAAGCTGACAAGCTCTCCGGGCCGCGTAGCGTCGGGCAGAATTGATACATCTCCAGCAGAATAGCGACGTAGTCCCGCGCTTCCGCATGCAGGTCGAACGAATCGGGGGCAAACAGCCAGTTTTCCATCAGCCCGGAAAGGTAGCTGCGCATTAACACGGCCGCCCGCCGGGTGAGTAAATTGGCGGGCAGCAGCTTCGCCGCGATGCACTCTTTCAAGGTCTGCTCGATACGCTCATAACTCGCCAGGGAGAGCTGCCGCTGGGCCTGCTGCACCACGGTCATTTCACCGACGAACTCACACTTATGATAGATAATCTCCATCATTAATCGTCGACGTTCTTCTGTCACTGTCGCTTCAAGAACATAGACTAGAATCTCCCTGATAACTGAGAGTGGATCGTTGGGGAATTTTGCCCGATACTCAATTTCGAGATCGCTAATACTGGCGTCTGACAGCTCCCAAATTTCGTTGAATAAATCTGATTTATTCTTGAAATGCCAGTAGATAGCCCCCCTCGTTACACCCGCAGCTTTTGCAATTGTTGCCAACGAGGTAGATGATACGCCTTGCTGCGAAAACAGACGCAGAGCAACATCCAGAATCAGTTGCCGGGTTTCACGTGCCTGTTGTTTGGTTTTTCGTGCCATAGGTTAATGACTTTACAGAGGTTACGTTTACATACATTTGTGAATGTATGTACCATAGCATGACCATAATAGAAAGACTGTAGTGGGTTTGTGGTTGTTTGAGCCACTGAACATTTTGAAATTGGACACTCGAGGTTTACATATGAACAAAAACAGAGGGTTAACGCCTCTGGCGGTCGTCTGATGTTCTCTGGG

>acrR-11 CGCTGCGGCGCGCAGATAGCACTTCTCAGCGCTGGCGAATAGCGGCGGACGGCGCTAAAAATTCACGCCCATCCGCGCGCTACCTTTAAGCTGACAAGCTCTCCGGGCCGCGTAGCGTCGGGCAGAATTGATACATCTCCAGCAGAATAGCGACGTAGTCCCGCGCTTCCGCATGCAGGTCGAACGAATCGGGGGCAAACAGCCAGTTTTCCATCAGCCCGGAAAGGTAGCTGCGCATTAACACGGCCGCCCGCCGGGTGAGTAAATTGGCGGGCAGCAGCTTCGCCGCGATGCACTCTTTCAAGGTCTGCTCGATACGCTCATAACTCGCCAGGGAGAGCTGCCGCTGGGCCTGCTGCACCACGGTCATTTCACCGACGAACTCACACTTATGATAGATAATCTCCATCATTAATCGTCGACGTTCTTCTGTCACTGTCGCTTCAAGAACATAGACTAGAATCTCCCTGATAACTGAGAGTGGATCGTTGGGGAATTTTGCCCGATACTCAATTTCGAGATCGCTAATACTGGCGTCTGACAGCTCCCAAATTTCGTTGAATAAATCTGATTTATTCTTGAAATGCCAGTAGATAGCCCCCCTCGTTACACCCGCAGCTTTTGCAATTGTTGCCAACGAGGTAGATGATACGCCTTGCTGCGAAAACAGACGCAGAGCAACATCCAGAATCAGTTGCCGGGTTTCACGTGCCTGTTGTTTGGTTTTTCGTGCCATAGGTTAATGACTTTACAGAGGTTACGTTTACATACATTTGTGAATGTATGTACCATAGCATGACCATAATAGAAAGACTGTAGTGGGTTTGTGGTTGTTTGAGCCACTGAACATTTTGAAATTGGACACTCGAGGTTTACATATGAACAAAAACAGAGGGTTAACGCCTCTGGCGGTCGTCTGAGGTCCTCCGGGG

>acrR-12 GGTTCGAGCGCGCGATAGCACTTCTCAGCGCTGGCGAATAGCGGCGGACGGCGCTAAAAATTCACGCCCATCCGCGCGCTACCTTTAAGCTGACAAGCTCTCCGGGCCGCGTAGCGTCGGGCAGAATTGATACATCTCCAGCAGAATAGCGACGTAGTCCCGCGCTTCCGCATGCAGGTCGAACGAATCGGGGGCAAACAGCCAGTTTTCCATCAGCCCGGAAAGGTAGCTGCGCATTAACACGGCCGCCCGCCGGGTGAGTAAATTGGCGGGCAGCAGCTTCGCCGCGATGCACTCTTTCAAGGTCTGCTCGATACGCTCATAACTCGCCAGGGAGAGCTGCCGCTGGGCCTGCTGCACCACGGTCATTTCACCGACGAACTCACACTTATGATAGATAATCTCCATCATTAATCGTCGACGTTCTTCTGTCACTGTCGCTTCAAGAACATAGACTAGAATCTCCCTGATAACTGAGAGTGGATCGTTGGGGAATTTTGCCCGATACTCAATTTCGAGATCGCTAATACTGGCGTCTGACAGCTCCCAAATTTCGTTGAATAAATCTGATTTATTCTTGAAATGCCAGTAGATAGCCCCCCTCGTTACACCCGCAGCTTTTGCAATTGTTGCCAACGAGGTAGATGATACGCCTTGCTGCGAAAACAGACGCAGAGCAACATCCAGAATCAGTTGCCGGGTTTCACGTGCCTGTTGTTTGGTTTTTCGTGCCATAGGTTAATGACTTTACAGAGGTTACGTTTACATACATTTGTGAATGTATGTACCATAGCATGACCATAATAGAAAGACTGTAGTGGGTTTGTGGTTGTTTGAGCCACTGAACATTTTGAAATTGGACACTCGAGGTTTACATATGAACAAAAACAGAGGGTTAACGCCTCTGGCGGTCGTCTGAGTTCTCCTCGGG

>acrR-14

GGTCTCGCGCGCGCAGATAGCACTTCTCAGCGCTGGCGAATAGCGGCGGACGGCGCTAAAAATTCACGCCCATCCGCGCGCTACCTTTAAGCTGACAAGCTCTCCGGGCCGCGTAGCGTCGGGCAGAATTGATACATCTCCAGCAGAATAGCGACGTAGTCCCGCGCTTCCGCATGCAGGTCGAACGAATCGGGAGCAAACAGCCAGTTTTCCATCAGCCCGGAAAGGTAGCTGCGCATTAACACGGCCGCCCGCCGGGTGAGTAAATTGGCGGGCAGCAGCTTCGCCGCGATGCACTCTTTCAAGGTCTGCTCGATACGCTCATAACTCGCCAGGGAGAGCTGCCGCTGGGCCTGCTGCACCACGGTCATTTCACCGACGAACTCACACTTATGATAGATAATCTCCATCATTAATCGTCGACGTTCTTCTGTCACTGTCGCTTCAAGAACATAGACTAGAATCTCCCTGATAACTGAGAGTGGATCGTTGGGGAATTTTGCCCGATACTCAATTTCGAGATCGCTAATACTGGCGTCTGACAGCTCCCAAATTTCGTTGAATAAATCTGATTTATTCTTGAAATGCCAGTAGATAGCCCCCCTCGTTACACCCGCAGCTTTTGCAATTGTTGCCAACGAGGTAGATGATACGCCTTGCTGCGAAAACAGACGCAGAGCAACATCCAGAATCAGTTGCCGGGTTTCACGTGCCTGTTGTTTGGTTTTTCGTGCCATAGGTTAATGACTTTACAGAGGTTACGTTTACATACATTTGTGAATGTATGTACCATAGCATGACCATAATAGAAAGACTGTAGTGGGTTTGTGGTTGTTTGAGCCACTGAACATTTTGAAATTGGACACTCGAGGTTTACATATGAACAAAAACCGAGGGTTAACGCCTCTGGCGGCGTCTGTGTC

>acrR-15 CTTCAGCGCGCGAATAGCACTTCTCAGCGCTGGCGAATAGCGGCGGACGGCGCTAAAAATTCACGCCCATCCGCGCGCTACCTTTAAGCTGACAAGCTCTCCGGGCCGCGTAGCGTCGGGCAGAATTGATACATCTCCAGCAGAATAGCGACGTAGTCCCGCGCTTCCGCATGCAGGTCGAACGAATCGGGGGCAAACAGCCAGTTTTCCATCAGCCCGGAAAGGTAGCTGCGCATTAACACGGCCGCCCGCCGGGTGAGTAAATTGGCGGGCAGCAGCTTCGCCGCGATGCACTCTTTCAAGGTCTGCTCGATACGCTCATAACTCGCCAGGGAGAGCTGCCGCTGGGCCTGCTGCACCACGGTCATTTCACCGACGAACTCACACTTATGATAGATAATCTCCATCATTAATCGTCGACGTTCTTCTGTCACTGTCGCTTCAAGAACATAGACTAGAATCTCCCTGATAACTGAGAGTGGATCGTTGGGGAATTTTGCCCGATACTCAATTTCGAGATCGCTAATACTGGCGTCTGACAGCTCCCAAATTTCGTTGAATAAATCTGATTTATTCTTGAAATGCCAGTAGATAGCCCCCCTCGTTACACCCGCAGCTTTTGCAATTGTTGCCAACGAGGTAGATGATACGCCTTGCTGCGAAAACAGACGCAGAGCAACATCCAGAATCAGTTGCCGGGTTTCACGTGCCTGTTGTTTGGTTTTTCGTGCCATAGGTTAATGACTTTACAGAGGTTACGTTTACATACATTTGTGAATGTATGTACCATAGCATGACCATAATAGAAAGACTGTAGTGGGTTTGTGGTTGTTTGAGCCACTGAACATTTTGAAATTGGACACTCGAGGTTTACATATGAACAAAAACAGAGGGTTAACGCCTCTGGCGGACGTTCTGATGC

>acrR-16 GTTTGGCGCGCGCGATAGCACTTCTCAGCGCTGGCGAATAGCGGCGGACGGCGCTAAAAATTCACGCCCATCCGCGCGCTACCTTTAAGCTGACAAGCTCTCCGGGCCGCGTAGCGTCGGGCAGAATTGATACATCTCCAGCAGAATAGCGACGTAGTCCCGCGCTTCCGCATGCAGGTCGAACGAATCGGGGGCAAACAGCCAGTTTTCCATCAGCCCGGAAAGGTAGCTGCGCATTAACACGGCCGCCCGCCGGGTGAGTAAATTGGCGGGCAGCAGCTTCGCCGCGATGCACTCTTTCAAGGTCTGCTCGATACGCTCATAACTCGCCAGGGAGAGCTGCCGCTGGGCCTGCTGCACCACGGTCATTTCACCGACGAACTCACACTTATGATAGATAATCTCCATCATTAATCGTCGACGTTCTTCTGTCACTGTCGCTTCAAGAACATAGACTAGAATCTCCCTGATAACTGAGAGTGGATCGTTGGGGAATTTTGCCCGATACTCAATTTCGAGATCGCTAATACTGGCGTCTGACAGCTCCCAAATTTCGTTGAATAAATCTGATTTATTCTTGAAATGCCAGTAGATAGCCCCCCTCGTTACACCCGCAGCTTTTGCAATTGTTGCCAACGAGGTAGATGATACGCCTTGCTGCGAAAACAGACGCAGAGCAACATCCAGAATCAGTTGCCGGGTTTCACGTGCCTGTTGTTTGGTTTTTCGTGCCATAGGTTAATGACTTTACAGAGGTTACGTTTACATACATTTGTGAATGTATGTACCATAGCATGACCATAATAGAAAGACTGTAGTGGGTTTGTGGTTGTTTGAGCCACTGAACATTTTGAAATTGGACACTCGAGGTTTACATATGAACAAAAACAGAGGGTTAACGCCTCTGGCGGTCGTTTGAGGTTCT

>acrR-17 CCCCAAGCGCGCGATAGCACTTCTCAGCGCTGGCGAATAGCGGCGGACGGCGCTAAAAATTCACGCCCATCCGCGCGCTACCTTTAAGCTGACAAGCTCTCCGGGCCGCGTAGCGTCGGGCAGAATTGATACATCTCCAGCAGAATAGCGACGTAGTCCCGCGCTTCCGCATGCAGGTCGAACGAATCGGGGGCAAACAGCCAGTTTTCCATCAGCCCGGAAAGGTAGCTGCGCATTAACACGGCCGCCCGCCGGGTGAGTAAATTGGCGGGCAGCAGCTTCGCCGCGATGCACTCTTTCAAGGTCTGCTCGATACGCTCATAACTCGCCAGGGAGAGCTGCCGCTGGGCCTGCTGCACCACGGTCATTTCACCGACGAACTCACACTTATGATAGATAATCTCCATCATTAATCGTCGACGTTCTTCTGTCACTGTCGCTTCAAGAACATAGACTAGAATCTCCCTGATAACTGAGAGTGGATCGTTGGGGAATTTTGCCCGATACTCAATTTCGAGATCGCTAATACTGGCGTCTGACAGCTCCCAAATTTCGTTGAATAAATCTGATTTATTCTTGAAATGCCAGTAGATAGCCCCCCTCGTTACACCCGCAGCTTTTGCAATTGTTGCCAACGAGGTAGATGATACGCCTTGCTGCGAAAACAGACGCAGAGCAACATCCAGAATCAGTTGCCGGGTTTCACGTGCCTGTTGTTTGGTTTTTCGTGCCATAGGTTAATGACTTTACAGAGGTTACGTTTACATACATTTGTGAATGTATGTACCATAGCATGACCATAATAGAAAGACTGTAGTGGGTTTGTGGTTGTTTGAGCCACTGAACATTTTGAAATTGGACACTCGAGGTTTACATATGAACAAAAACAGAGGGTTAACGCCTCTGGCGGTCGTTCTGAGGTCTCCCGGCAGCTTAACAA

>acrR-18 TGCAGGCGCGCAGATAGCACTTCTCAGCGCTGGCGAATAGCGGCGGACGGCGCTAAAAATTCACGCCCATCCGCGCGCTACCTTTAAGCTGACAAGCTCTCCGGGCCGCGTAGCGTCGGGCAGAATTGATACATCACCAGCAGAATAGCGACGTAGTCCCGCGCTTCCGCATGCAGGTCGAACGAATCGGGGGCAAACAGCCAGTTTTCCATCAGCCCGGAAAGGTAGCTGCGCATTAACACGGCCGCCCGCCGGGTGAGTAAATTGGCGGGCAGCAGCTTCGCCGCGATGCACTCTTTCAAGGTCTGCTCGATACGCTCATAACTCGCCAGGGAGAGCTGCCGCTGGGCCTGCTGCACCACGGTCATTTCACCGACGAACTCACACTTATGATAGATAATCTCCATCATTAATCGTCGACGTTCTTCTGTCACTGTCGCTTCAAGAACATAGACTAGAATCTCCCTGATAACTGAGAGTGGATCGTTGGGGAATTTTGCCCGATACTCAATTTCGAGATCGCTAATACTGGCGTCTGACAGCTCCCAAATTTCGTTGAATAAATCTGATTTATTCTTGAAATGCCAGTAGATAGCCCCCCTCGTTACACCCGCAGCTTTTGCAATTGTTGCCAACGAGGTAGATGATACGCCTTGCTGCGAAAACAGACGCAGAGCAACATCCAGAATCAGTTGCCGGGTTTCACGTGCCTGTTGTTTGGTTTTTCGTGCCATAGGTTAATGACTTTACAGAGGTTACGTTTACATACATTTGTGAATGTATGTACCATAGCATGACCATAATAGAAAGACTGTAGTGGGTTTGTGGTTGTTTGAGCCACTGAACATTTTGAAATTGGACACTCGAGGTTTACATATGAACAAAAACAGAGGGTTAACGCCTCTGGCGGTCGTCTGAGGTCCCCCGGGAG

>acrR-19 GGGTCTGCGCGCGCAGATAGCACTTCTCAGCGCTGGCGAATAGCGGCGGACGGCGCTAAAAATTCACGCCCATCCGCGCGCTACCTTTAAGCTGACAAGCTCTCCGGGCCGCGTAGCGTCGGGCAGAATTGATACATCTCCAGCAGAATAGCGACGTAGTCCCGCGCTTCCGCATGCAGGTCGAACGAATCGGGGGCAAACAGCCAGTTTTCCATCAGCCCGGAAAGGTAGCTGCGCATTAACACGGCCGCCCGCCGGGTGAGTAAATTGGCGGGCAGCAGCTTCGCCGCGATGCACTCTTTCAAGGTCTGCTCGATACGCTCATAACTCGCCAGGGAGAGCTGCCGCTGGGCCTGCTGCACCACGGTCATTTCACCGACGAACTCACACTTATGATAGATAATCTCCATCATTAATCGTCGACGTTCTTCTGTCACTGTCGCTTCAAGAACATAGACTAGAATCTCCCTGATAACTGAGAGTGGATCGTTGGGGAATTTTGCCCGATACTCAATTTCGAGATCGCTAATACTGGCGTCTGACAGCTCCCAAATTTCGTTGAATAAATCTGATTTATTCTTGAAATGCCAGTAGATAGCCCCCCTCGTTACACCCGCAGCTTTTGCAATTGTTGCCAACGAGGTAGATGATACGCCTTGCTGCGAAAACAGACGCAGAGCAACATCCAGAATCAGTTGCCGGGTTTCACGTGCCTGTTGTTTGGTTTTTCGTGCCATAGGTTAATGACTTTACAGAGGTTACGTTTACATACATTTGTGAATGTATGTACCATAGCATGACCATAATAGAAAGACTGTAGTGGGTTTGTGGTTGTTTGAGCCACTGAACATTTTGAAATTGGACACTCGAGGTTTACATATGAACAAAAACAGAGGGTTAACGCCTCTGGCGTCGTCTGTGTCC

>acrR-20 GGCTGCAGCGCGCAGATAGCACTTCTCAGCGCTGGCGAATAGCGGCGGACGGCGCTAAAAATTCACGCCCATCCGCGCGCTACCTTTAAGCTGACAAGCTCTCCGGGCCGCGTAGCGTCGGGCAGAATTGATACATCTCCAGCAGAATAGCGACGTAGTCCCGCGCTTCCGCATGCAGGTCGAACGAATCGGGGGCAAACAGCCAGTTTTCCATCAGTCCGGAAAGGTAGCTGCGCATTAACACGGCCGCCCGCCGGGTGAGTAAATTGGCGGGCAGCAGCTTAGCCGCGATGCACTCTTTCAAGGTCTGCTCGATACGCTCATAACTCGCCAGGGAGAGCTGCCGCTGGGCCTGCTGCACCACGGTCATTTCACCGACGAACTCACACTTATGATAGATAATCTCCATCATTAATCGTCGACGTTCTTCTGTCACTGTCGCTTCAAGAACATAGACTAGAATCTCCCTGATAACTGAGAGTGGATCGTTGGGGAATTTTGCCCGATACTCAATTTCGAGATCGCTAATACTGGCGTCTGACAGCTCCCAAATTTCGTTGAATAAATCTGATTTATTCTTGAAATGCCAGTAGATAGCCCCCCTCGTTACACCCGCAGCTTTTGCAATTGTTGCCAACGAGGTAGATGATACGCCTTGCTGCGAAAACAGACGCAGAGCAACATCCAGAATCAGTTGCCGGGTTTCACGTGCCTGTTGTTTGGTTTTTCGTGCCATAGGTTAATGACTTTACAGAGGTTACGTTTACATACATTTGTGAATGTATGTACCATAGCATGACCATAATAGAAAGACTGTAGTGGGTTTGTGGTTGTTTGAGCCACTGAACATTTTGAAATTGGACACTCGAGGTTTACATATGAACAAAAACAGAGGGTTAACGCCTCTGGGGTCGTCTGTGTCTC

>acrR-21 CGCCGCCGCGCAGAATAGCACTTCTCAGCGCTGGCGAATAGCGGCGGACGGCGCTAAAAATTCACGCCCATCCGCGCGCTACCTTTAAGCTGACAAGCTCTCCGGGCCGCGTAGCGTCGGGCAGAATTGATACATCTCCAGCAGAATAGCGACGTAGTCCCGCGCTTCCGCATGCAGGTCGAACGAATCGGGGGCAAACAGCCAGTTTTCCATCAGCCCGGAAAGGTAGCTGCGCATTAACACGGCCGCCCGCCGGGTGAGTAAATTGGCGGGCAGCAGCTTCGCCGCGATGCACTCTTTCAAGGTCTGCTCGATACGCTCATAACTCGCCAGGGAGAGCTGCCGCTGGGCCTGCTGCACCACGGTCATTTCACCGACGAACTCACACTTATGATAGATAATCTCCATCATTAATCGTCGACGTTCTTCTGTCACTGTCGCTTCAAGAACATAGACTAGAATCTCCCTGATAACTGAGAGTGGATCGTTGGGGAATTTTGCCCGATACTCAATTTCGAGATCGCTAATACTGGCGTCTGACAGCTCCCAAATTTCGTTGAATAAATCTGATTTATTCTTGAAATGCCAGTAGATAGCCCCCCTCGTTACACCCGCAGCTTTTGCAATTGTTGCCAACGAGGTAGATGATACGCCTTGCTGCGAAAACAGACGCAGAGCAACATCCAGAATCAGTTGCCGGGTTTCACGTGCCTGTTGTTTGGTTTTTCGTGCCATAGGTTAATGACTTTACAGAGGTTACGTTTACATACATTTGTGAATGTATGTACCATAGCATGACCATAATAGAAAGACTGTAGTGGGTTTGTGGTTGTTTGAGCCACTGAACATTTTGAAATTGGACACTCGAGGTTTACCTATGACCAAAAACCGAGGGTTAACGCCTCTGGCGTCGTCTGTGTTCTCTC

>acrR-24 CTCCAGGCGCGCGATAGCACTTCTCAGCGCTGGCGAATAGCGGCGGACGGCGCTAAAAATTCACGCCCATCCGCGCGCTACCTTTAAGCTGACAAGCTCTCCGGGCCGCGTAGCGTCGGGCAGAATTGATACATCTCCAGCAGAATAGCGACGTAGTCCCGCGCTTCCGCATGCAGGTCGAACGAATCGGGGGCAAACAGCCAGTTTTCCATCAGCCCGGAAAGGTAGCTGCGCATTAACACGGCCGCCCGCCGGGTGAGTAAATTGGCGGGCAGCAGCTTCGCCGCGATGCACTCTTTCAAGGTCTGCTCGATACGCTCATAACTCGCCAGGGAGAGCTGCCGCTGGGCCTGCTGCACCACGGTCATTTCACCGACGAACTCACACTTATGATAGATAATCTCCATCATTAATCGTCGACGTTCTTCTGTCACTGTCGCTTCAAGAACATAGACTAGAATCTCCCTGATAACTGAGAGTGGATCGTTGGGGAATTTTGCCCGATACTCAATTTCGAGATCGCTAATACTGGCGTCTGACAGCTCCCAAATTTCGTTGAATAAATCTGATTTATTCTTGAAATGCCAGTAGATAGCCCCCCTCGTTACACCCGCAGCTTTTGCAATTGTTGCCAACGAGGTAGATGATACGCCTTGCTGCGAAAACAGACGCAGAGCAACATCCAGAATCAGTTGCCGGGTTTCACGTGCCTGTTGTTTGGTTTTTCGTGCCATAGGTTAATGACTTTACAGAGGTTACGTTTACATACATTTGTGAATGTATGTACCATAGCATGACCATAATAGAAAGACTGTAGTGGGTTTGTGGTTGTTTGAGCCACTGAACATTTTGAAATTGGACACTCGAGGTTTACATATGAACAAAAACAGAGGGTTAACGCCTCTGGCGGTCGTCTGAGGTCTTCGGGGGAGTTTAGAAAA

>acrR-25 GGACTGCGCGCGCGATAGCACTTCTCAGCGCTGGCGAATAGCGGCGGACGGCGCTAAAAATTCACGCCCATCCGCGCGCTACCTTTAAGCTGACAAGCTCTCCGGGCCGCGTAGCGTCGGGCAGAATTGATACATCTCCAGCAGAATAGCGACGTAGTCCCGCGCTTCCGCATGCAGGTCGAACGAATCGGGGGCAAACAGCCAGTTTTCCATCAGCCCGGAAAGGTAGCTGCGCATTAACACGGCCGCCCGCCGGGTGAGTAAATTGGCGGGCAGCAGCTTCGCCGCGATGCACTCTTTCAAGGTCTGCTCGATACGCTCATAACTCGCCAGGGAGAGCTGCCGCTGGGCCTGCTGCACCACGGTCATTTCACCGACGAACTCACACTTATGATAGATAATCTCCATCATTAATCGTCGACGTTCTTCTGTCACTGTCGCTTCAAGAACATAGACTAGAATCTCCCTGATAACTGAGAGTGGATCGTTGGGGAATTTTGCCCGATACTCAATTTCGAGATCGCTAATACTGGCGTCTGACAGCTCCCAAATTTCGTTGAATAAATCTGATTTATTCTTGAAATGCCAGTAGATAGCCCCCCTCGTTACACCCGCAGCTTTTGCAATTGTTGCCAACGAGGTAGATGATACGCCTTGCTGCGAAAACAGACGCAGAGCAACATCCAGAATCAGTTGCCGGGTTTCACGTGCCTGTTGTTTGGTTTTTCGTGCCATAGGTTAATGACTTTACAGAGGTTACGTTTACATACATTTGTGAATGTATGTACCATAGCATGACCATAATAGAAAGACTGTAGTGGGTTTGTGGTTGTTTGAGCCACTGAACATTTTGAAATTGGACACTCGAGGTTTACATATGAACAAAAACAGAGGGTTAACGCCTCTGGCGTCGTTTGAGT

>acrR-26 TCCCAGCGCGCGAATAGCACTTCTCAGCGCTGGCGAATAGCGGCGGACGGCGCTAAAAATTCATGCCCATCCGCGCGCTACCTTTAAGCTGACAAGCTCTCCGGGCCGCGTAGCGTCGGGCAGAATTGATACATCTCCAGCAGAATAGCGACGTAGTCCCGCGCTTCCGCATGCAGGTCGAACGAATCGGGGGCAAACAGCCAGTTTTCCATCAGCCCGGAAAGGTAGCTGCGCATTAACACGGCCGCCCGCCGGGTGAGTAAATTGGCGGGCAGCAGCTTCGCCGCGATGCACTCTTTCAAGGTCTGCTCGATACGCTCATAACTCGCCAGGGAGAGCTGCCGCTGGGCCTGCTGCACCACGGTCATTTCACCGACGAACTCACACTTATGATAGATAATCTCCATCATTAATCGTCGACGTTCTTCTGTCACTGTCGCTTCAAGAACATAGACTAGAATCTCCCTGATAACTGAGAGTGGATCGTTGGGGAATTTTGCCCGATACTCAATTTCGAGATCGCTAATACTGGCGTCTGACAGCTCCCAAATTTCGTTGAATAAATCTGATTTATTCTTGAAATGCCAGTAGATAGCCCCCCTCGTTACACCCGCAGCTTTTGCAATTGTTGCCAACGAGGTAGATGATACGCCTTGCTGCGAAAACAGACGCAGAGCAACATCCAGAATCAGTTGCCGGGTTTCACGTGCCTGTTGTTTGGTTTTTCGTGCCATAGGTTAATGACTTTACAGAGGTTACGTTTACATACATTTGTGAATGTATGTACCATAGCATGACCATAATAGAAAGACTGTAGTGGGTTTGTGGTTGTTTGAGCCACTGAACATTTTGAAATTGGACACTCGAGGTTTACATATGAACAAAAACAGAGGGTTAACGCCTCTGGCGGTCGTTCTGATGTTCTCCAGGGCAGCTTA

>acrR-27 GGGCTCAGCGCGCGATAGCACTTCTCAGCGCTGGCGAATAGCGGCGGACGGCGCTAAAAATTCACGCCCATCCGCGCGCTACCTTTAAGCTGACAAGCTCTCCGGGCCGCGTAGCGTCGGGCAGAATTGATACATCTCCAGCAGAATAGCGACGTAGTCCCGCGCTTCCGCATGCAGGTCGAACGAATCGGGGGCAAACAGCCAGTTTTCCATCAGTCCGGAAAGGTAGCTGCGCATTAACACGGCCGCCCGCCGGGTGAGTAAATTGGCGGGCAGCAGCTTCGCCGCGATGCACTCTTTCAAGGTCTGCTCGATACGCTCATAACTCGCCAGGGAGAGCTGCCGCTGGGCCTGTTGCACCACGGTCATTTCACCGACGAACTCACACTTATGATAGATAATCTCCATCATTAATCGTCGACGTTCTTCTGTCACTGTCGCTTCAAGAACATAGACTAGAATCTCCCTGATAACTGAGAGTGGATCGTTGGGGAATTTTGCCCGATACTCAATTTCGAGATCGCTAATACTGGCGTCTGACAGCTCCCAAATTTCGTTGA

>acrR-28 GGGCTGCGCGCGCGATAGCACTTCTCAGCGCTGGCGAATAGCGGCGGACGGCGCTAAAAATTCACGCCCATCCGCGCGCTACCTTTAAGCTGACAAGCTCTCCGGGCCGCGTAGCGTCGGGCAGAATTGATACATCTCCAGCAGAATAGCGACGTAGTCCCGCGCTTCCGCATGCAGGTCGAACGAATCGGGGGCAAACAGCCAGTTTTCCATCAGCCCGGAAAGGTAGCTGCGCATTAACACGGCCGCCCGCCGGGTGAGTAAATTGGCGGGCAGCAGCTTCGCCGCGATGCACTCTTTCAAGGTCTGCTCGATACGCTCATAACTCGCCAGGGAGAGCTGCCGCTGGGCCTGCTGCACCACGGTCATTTCACCGACGAACTCACACTTATGATAGATAATCTCCATCATTAATCGTCGACGTTCTTCTGTCACTGTCGCTTCAAGAACATAGACTAGAATCTCCCTGATAACTGAGAGTGGATCGTTGGGGAATTTTGCCCGATACTCAATTTCGAGATCGCTAATACTGGCGTCTGACAGCTCCCAAATTTCGTTGAATAAATCTGATTTATTCTTGAAATGCCAGTAGATAGCCCCCCTCGTTACACCCGCAGCTTTTGCAATTGTTGCCAACGAGGTAGATGATACGCCTTGCTGCGAAAACAGACGCAGAGCAACATCCAGAATCAGTTGCCGGGTTTCACGTGCCTGTTGTTTGGTTTTTCGTGCCATAGGTTAATGACTTTACAGAGGTTACGTTTACATACATTTGTGAATGTATGTACCATAGCATGACCATAATAGAAAGACTGTAGTGGGTTTGTGGTTGTTTGAGCCACTGAACATTTTGAAATTGGACACTCGAGGTTTACATATGAACAAAAACAGAGGGTTAACGCCTCTGGGGGCGTCTGTGTGTTCCC

>acrR-30

ATGGCACGAAAAACCAAACAACAGGCACGTGAAACCCGGCAACTGATTCTGGATGTTGCTCTGCGTCTGTTTTCGCAGCAAGGCGTATCATCTACCTCGTTGGCAACAATTGCAAAAGCTGCGGGTGTAACGAGGGGGGCTATCTACTGGCATTTCAAGAATAAATCAGATTTATTCAACGAAATTTGGGAGCTGTCAGACGCCAGTATTAGCGATCTCGAAATTGAGTATCGGGCAAAATTCCCCAACGATCCACTCTCAGTTATCAGGGAGATTCTAGTCTATGTTCTTGAAGCGACAGTGACAGAAGAACGTCGACGATTAATGATGGAGATTATCTATCATAAGTGTGAGTTCGTCGGTGAAATGACCGTGGTGCAGCAGGCCCAGCGGCAGCTCTCCCTGGCGAGTTATGAGCGTATCGAGCAGACCTTGAAAGAGTGCATCGCGGCTAAGCTGCTGCCCGCCAATTTACTCACCCGGCGGGCGGCCGTGTTAATGCGCAGCTACCTTTCCGGACTGATGGAAAACTGGCTGTTTGCCCCCGATTCGTTCGACCTGCATGCGGAAGCGCGGGACTACGTCGCTATTCTGCTGGAGATGTATCAATTCTGCCCGACGCTACGCGGCCCGGAGAGCTTGTCAGCTTAA

>oqxR-1 AAAATGTTAGATTACCGCTTCCCGACAGCTTTGCAGATGGTTCTCAGCGTAGCAATGGCGGAGCAGATGGGTGGACGTTCGACGAGTGCGATTCTGGCCTACGGCCTGGAAGCGAACCCGAGCTTTATCCGTAAACTAATGGTTCCGCTAACTCGTGACGGCATAATCGTCTCCACGCTTGGCCGCAACGGCTCAATTCATCTTGGCCGTCCGGCGGACAAGATCACCCTGCGTGATATCTATCTTTCGGTTATCGAAGATAAAAAACTGTGGGCGTCGCGTCCTGACGTCCCGGCCCGCTGCGTGGTCAGCGCCAACGCCTGCTGGTACTTCAAATCGGTTGCCGATGAAGCAGAGCAGGCTTCGTTAAACGTCCTCGCTCGCCATACCGTGGCCAGCGCGCTGGAGGCGGTCAAAAACGCCGATACCAGCGGCTGCGACCCGGTGCCGGAAATGATCGCCCGCTTTAAAAAAGCGCATTAAAAa

>oqxR-2 TTCAGGCTGCGCAGATTGCTTCAAAAAATTCCCGTATTTGTTATATTGTTGTTCATTGATTATTCACCTCTGCGGTGCCAAAAAGAACAAGATTCACCGCAACCCAGGACACTGCCATGTTAGATTACCGCTTCCCGACAGCTTTGCAGATGGTTCTCAGCGTAGCAATGGCGGAGCAGATGGGTGAACGTTCGACGAGTGCGATTCTGGCCTACGGCCTGGAAGCGAACCCGAGCTTTATCCGTAAACTAATGGTTCCGCTAACTCGTGACGGCATTATCGTCTCCACGCTTGGCCGCAACGGCTCAATTCATCTTGGCCGTCCGGCGGACAAGATCACCCTGCGTGATATCTATCTTTCGGTTATCGAAGATAAAAAACTGTGGGCGTCGCGTCCTGACGTCCCGGCCCGCTGCGTGGTCAGCGCCAACGCCTGCTGGTACTTCAAATCGGTTGCCGATGAAGCAGAGCAGGCTTCGTTAAACGTCCTCGCTCGCCATACCGTGGCCAGCGCGCTGGAGGCGGTCAAAAACGCCGATACCAGCGGCTGCGACCCGGTGCCGGAAATGATCGCCCGTTTTAAAAAAGCGCATTATTTTTTTTGGGGGGGAAAAAAAAAGGCCGGC

>oqxR-3 CTTTCGAGGCTACCGCAGATTGCTTCAAAAAATTCCCGTATTTGTTATATTGTTGTTCATTGATTATTCACCTCTGCGGTGCCAAAAAGAACAAGATTCACCGCAACCCAGGACACTGCCATGTTAGATTACCGCTTCCCGACAGCTTTGCAGATGGTTCTCAGCGTAGCAATGGCGGAGCAGATGGGTGAACGTTCGACGAGTGCGATTCTGGCCTACGGCCTGGAAGCGAACCCGAGCTTTATCCGTAAACTAATGGTTCCGCTAACTCGTGACGGCATTATCGTCTCCACGCTTGGCCGCAACGGCTCAATTCATCTTGGCCGTCCGGCGGACAAGATCACCCTGCGTGATATCTATCTTTCGGTTATCGAAGATAAAAAACTGTGGGCGTCGCGTCCTGACGTCCCGGCCCGCTGCGTGGTCAGCGCCAACGCCTGCTGGTACTTCAAATCGGTTGCCGATGAAGCAGAGCAGGCTTCGTTAAACGTCCTCGCTCGCCATACCGTGGCCAGCGCGCTGGAGGCGGTCAAAAACGCCGATACCAGCGGCTGCGACCCGGTGCCGGAAATGATCGCCCGCTTTAAAAAAGCGCATTAATCTTTTTGGGGGGGAAAAAAGGC

>oqxR-4 CTTCGCGGCTACCGCAGATTGCTTCAAAAAATTCCCGTATTTGTTATATTGTTGTTCATTGATTATTCACCTCTGCGGTGCCAAAAAGAACAAGATTCACCGCAACCCAGGACACTGCCATGTTAGATTACCGCTTCCCGACAGCTTTGCAGATGGTTCTCAGCGTAGCAATGGCGGAGCAGATGGGTGAACGTTCGACGAGTGCGATTCTGGCCTACGGCCTGGAAGCGAACCCGAGCTTTATCCGTAAACTAATGGTTCCGCTAACTCGTGACGGCATTATCGTCTCCACGCTTGGCCGCAACGGCTCAATTCATCTTGGCCGTCCGGCGGACAAGATCACCCTGCGTGATATCTATCTTTCGGTTATCGAAGATAAAAAACTGTGGGCGTCGCGTCCTGACGTCCCGGCCCGCTGCGTGGTCAGCGCCAACGCCTGCTGGTACTTCAAATCGGTTGCCGACGAAGCAGAGCAGGCTTCGTTAAACGTCCTCGCTCGCCATACCGTGGCCAGCGCGCTGGAGGCGGTCAAAAACGCCGATACCAGCGGCTGCGACCCGGTGCCGGAAATGATCGCCCGCTTTAAAAAAGCGCATTAATCATTTTTTGGGTGAAAAA

>oqxR-5 TTTTGAGGCTACCGCAGATTGCTTCAAAAAATTCCCGTATTTGTTATATTGTTGTTCATTGATTATTCACCTCTGCGGTGCCAAAAAGAACAAGATTCACCGCAACCCAGGACACTGCCATGTTAGATTACCGCTTCCCGACAGCTTTGCAGATGGTTCTCAGCGTAGCAATGGCGGAGCAGATGGGTGAACGTTCGACGAGTGCGATTCTGGCCTACGGCCTGGAAGCGAACCCGAGCTTTATCCGTAAACTAATGGTTCCGCTAACTCGTGACGGCATTATCGTCTCCACGCTTGGCCGCAACGGCTCAATTCATCTTGGCCGTCCGGCGGACAAGATCACCCTGCGTGATATCTATCTTTCGGTTATCGAAGATAAAAAACTGTGGGCGTCGCGTCCTGACGTCCCGGCCCGCTGCGTGGTCAGCGCCAACGCCTGCTGGTACTTCAAATCGGTTGCCGACGAAGCAGAGCAGGCTTCGTTAAACGTCCTCGCTCGCCATACCGTGGCCAGCGCGCTGGAGGCGGTCAAAAACGCCGATACCAGCGGCTGCGACCCGGTGCCGGAAATGATCGCCCGCTTTAAAAAAGCGCATTAATCTTTTTTGGGGGGAAAAAAACT

>oqxR-7 TATTCGCGGCTACCGCAGATTGCTTCAAAAAATTCCCGTATTTGTTATATTGTTGTTCATTGATTATTCACCTCTGCGGTGCCAAAAAGAACAAGATTCACCGCAACCCAGGACACTGCCATGTTAGATTACCGCTTCCCGACAGCTTTGCAGATGGTTCTCAGCGTAGCAATGGCGGAGCAGATGGGTGAACGTTCGACGAGTGCGATTCTGGCCTACGGCCTGGAAGCGAACCCGAGCTTTATCCGTAAACTAATGGTTCCGCTAACTCGTGACGGCATTATCGTCTCCACGCTTGGCCGCAACGGCTCAATTCATCTTGGCCGTCCGGCGGACAAGATCACCCTGCGTGATATCTATCTTTCGGTTATCGAAGATAAAAAACTGTGGGCGTCGCGTCCTGACGTCCCGGCCCGCTGCGTGGTCAGCGCCAACGCCTGCTGGTACTTCAAATCGGTTGCCGATGAAGCAGAGCAGGCTTCGTTAAACGTCCTCGCTCGCCATACCGTGGCCAGCGCGCTGGAGGCGGTCAAAAACGCCGATACCAGCGGCTGCGACCCGGTGCCGGAAATGATCGCCCGCTTTAAAAAAGCGCATTAATTTTTTTTGGGGGGAAAAAAAATAAGCG

>oqxR-8 TATTCGAGGCTACCGCAGATTGCTTCAAAAAATTCCCGTATTTGTTATATTGTTGTTCATTGATTATTCACCTCTGCGGTGCCAAAAAGAACAAGATTCACCGCAACCCAGGACACTGCCATGTTAGATTACCGCTTCCCGACAGCTTTGCAGATGGTTCTCAGCGTAGCAATGGCGGAGCAGATGGGTGAACGTTCGACGAGTGCGATTCTGGCCTACGGCCTGGAAGCGAACCCGAGCTTTATCCGTAAACTAATGGTTCCGCTAACTCGTGACGGCATTATCGTCTCCACGCTTGGCCGCAACGGCTCAATTCATCTTGGCCGTCCGGCGGACAAGATCACCCTGCGTGATATCTATCTTTCGGTTATCGAAGATAAAAAACTGTGGGCGTCGCGTCCTGACGTCCCGGCCCGCTGCGTGGTCAGCGCCAACGCCTGCTGGTACTTCAAATCGGTTGCCGATGAAGCAGAGCAGGCTTCGTTAAACGTCCTCGCTCGCCATACCGTGGCCAGCGCGCTGGAGGCGGTCAAAAACGCCGATACCAGCGGCTGCGACCCGGTGCCGGAAATGATCGCCCGCTTTAAAAAAGCGCATTAATCTTTTTTGTGGGGGGACAAAAA

>oqxR-9 TTTTAGAGGCTACCGCAGATTGCTTCAAAAAATTCCCGTATTTGTTATATTGTTGTTCATTGATTATTCACCTCTGCGGTGCCAAAAAGAACAAGATTCACCGCAACCCAGGACACTGCCATGTTAGATTACCGCTTCCCGACAGCTTTGCAGATGGTTCTCAGCGTAGCAATGGCGGAGCAGATGGGTGAACGTTCGACGAGTGCGATTCTGGCCTACGGCCTGGAAGCGAACCCGAGCTTTATCCGTAAACTAATGGTTCCGCTAACTCGTGACGGCATTATCGTCTCCACGCTTGGCCGCAACGGCTCAATTCATCTTGGCCGTCCGGCGGACAAGATCACCCTGCGTGATATCTATCTTTCGGTTATCGAAGATAAAAAACTGTGGGCGTCGCGTCCTGACGTCCCGGCCCGCTGCGTGGTCAGCGCCAACGCCTGCTGGTACTTCAAATCGGTTGCCGATGAAGCAGAGCAGGCTTCGTTAAACGTCCTCGCTCGCCATACCGTGGCCAGCGCGCTGGAGGCGGTCAAAAACGCCGATACCAGCGGCTGCGACCCGGTGCCGGAAATGATCGCCCGCTTTAAAAAAGCGCATTAATCTTTTTTGGGGGGGAAAAAAGTT

>oqxR-12 TATGCTAGCCGCAGATTGCTTCAAAAAATTCCCGTATTTGTTATATTGTTGTTCATTGATTATTCACCTCTGCGGTGCCAAAAAGAACAAGATTCACCGCAACCCAGGACACTGCCATGTTAGATTACCGCTTCCCGACAGCTTTGCAGATGGTTCTCAGCGTAGCAATGGCGGAGCAGATGGGTGAACGTTCGACGAGTGCGATTCTGGCCTACGGCCTGGAAGCGAACCCGAGCTTTATCCGTAAACTAATGGTTCCGCTAACTCGTGACGGCATTATCGTCTCCACGCTTGGCCGCAACGGCTCAATTCATCTTGGCCGTCCGGCGGACAAGATCACCCTGCGTGATATCTATCTTTCGGTTATCGAAGATAAAAAACTGTGGGCGTCGCGTCCTGACGTCCCGGCCCGCTGCGTGGTCAGCGCCAACGCCTGCTGGTACTTCAAATCGGTTGCCGATGAAGCAGAGCAGGCTTCGTTAAACGTCCTCGCTCGCCATACCGTGGCCAGCGCGCTGGAGGCGGTCAAAAACGCCGATACCAGCGGCTGCGACCCGGTGCCGGAAATGATCGCCCGCTTTAAAAAAGCGCATTAATCTTTTTTTGGGGGGAAAAAAAA

>oqxR-13 GCTGCCGCAGATTGCTTCGAAAAATTCCCGTATTTGTTATATTGTTGTTCATTGATTATTCACCTCTGCGGTGCCAAAAAGAACAAGATTCACCGCAACCCAGGACACTGCCATGTTAGATTACCGCTTCCCGACAGCTTTGCAGATGGTTCTCAGCGTAGCAATGGCGGAGCAGATGGGTGAACGTTCGACGAGTGCGATTCTGGCCTACGGCCTGGAAGCGAACCCGAGCTTTATCCGTAAACTAATGGTTCCGCTAACTCGTGACGGCATTATCGTCTCCACGCTTGGCCGCAACGGCTCAATTCATCTTGGCCGTCCGGCGGACAAGATCACCCTGCGTGATATCTATCTTTCGGTTATCGAAGATAAAAAACTGTGGGCGTCGCGTCCTGACGTCCCGGCCCGCTGCGTGGTCAGCGCCAACGCCTGCTGGTACTTCAAATCGGTTGCCGATGAAGCAGAGCAGGCTTCGTTAAACGTCCTCGCTCGCCATACCGTGGCCAGCGCGCTGGAGGCGGTCAAAAACGCCGATACCAGCGGCTGCGACCCGGTGCCGGAAATGATCGCCCGCTTTAAAAAAGCGCATTAATCATTTCCTGGGTGACAAAACCA

>oqxR-14 CCCCACAGATTGCTTCAAAAAATTCCCGTATTTGTTATATTGTTGTTCATTGATTATTCACCTCTGCGGTGCCAAAAAGAACAAGATTCACCGCAACCCAGGACACTGCCATGTTAGATTACCGCTTCCCGACAGCTTTGCAGATGGTTCTCAGCGTAGCAATGGCGGAGCAGATGGGTGAACGTTCGACGAGTGCGATTCTGGCCTACGGCCTGGAAGCGAACCCGAGCTTTATCCGTAAACTAATGGTTCCGCTAACTCGTGACGGCATTATCGTCTCCACGCTTGGCCGCAACGGCTCAATTCATCTTGGCCGTCCGGCGGACAAGATCACCCTGCGTGATATCTATCTTTCGGTTATCGAAGATAAAAAACTGTGGGCGTCGCGTCCTGACGTCCCGGCCCGCTGCGTGGTCAGCGCCAACGCCTGCTGGTACTTCAAATCGGTTGCCGATGAAGCAGAGCAGGCTTCGTTAAACGTCCTCGCTCGCCATACCGTGGCCAGCGCGCTGGAGGCGGTCAAAAACGCCGATACCAGCGGCTGCGACCCGGTGCCGGAAATGATCGCCCGTTTTAAAAAAGCGCATTAATTTTTTTGGGGGGGAAAAAAAACG

>oqxR-15 CTTCGAGGCTACCGCAGATTGCTTCAAAAAATTCCCGTATTTGTTATATTGTTGTTCATTGATTATTCACCTCTGCGGTGCCAAAAAGAACAAGATTCACCGCAACCCAGGACACTGCCATGTTAGATTACCGCTTCCCGACAGCTTTGCAGATGGTTCTCAGCGTAGCAATGGCGGAGCAGATGGGTGAACGTTCGACGAGTGCGATTCTGGCCTACGGCCTGGAAGCGAACCCGAGCTTTATCCGTAAACTAATGGTTCCGCTAACTCGTGACGGCATTATCGTCTCCACGCTTGGCCGCAACGGCTCAATTCATCTTGGCCGTCCGGCGGACAAGATCACCCTGCGTGATATCTATCTTTCGGTTATCGAAGATAAAAAACTGTGGGCGTCGCGTCCTGACGTCCCGGCCCGCTGCGTGGTCAGCGCCAACGCCTGCTGGTACTTCAAATCGGTTGCCGATGAAGCAGAGCAGGCTTCGTTAAACGTCCTCGCTCGCCATACCGTGGCCAGCGCGCTGGAGGCGGTCAAAAACGCCGATACCAGCGGCTGCGACCCGGTGCCGGAAATGATCGCCCGCTTTAAAAAAGCGCATTAATCTTTTTTGGTGGGGGAAAAAAAGATGGTC

>oqxR-16 TTTAGAGGCTACCGCAGATTGCTTCAAAAAATTCCCGTATTTGTTATATTGTTGTTCATTGATTATTCACCTCTGCGGTGCCAAAAAGAACAAGATTCACCGCAACCCAGGACACTGCCATGTTAGATTACCGCTTCCCGACAGCTTTGCAGATGGTTCTCAGCGTAGCAATGGCGGAGCAGATGGGTGAACGTTCGACGAGTGCGATTCTGGCCTACGGCCTGGAAGCGAACCCGAGCTTTATCCGTAAACTAATGGTTCCGCTAACTCGTGACGGCATTATCGTCTCCACGCTTGGCCGCAACGGCTCAATTCATCTTGGCCGTCCGGCGGACAAGATCACCCTGCGTGATATCTATCTTTCGGTTATCGAAGATAAAAAACTGTGGGCGTCGCGTCCTGACGTCCCGGCCCGCTGCGTGGTCAGCGCCAACGCCTGCTGGTACTTCAAATCGGTTGCCGATGAAGCAGAGCAGGCTTCGTTAAACGTCCTCGCTCGCCATACCGTGGCCAGCGCGCTGGAGGCGGTCAAAAACGCCGATACCAGCGGCTGCGACCCGGTGCCGGAAATGATCGCCCGCTTTAAAAAAGCGCATTAATCTTTTTTGGGGGGAAAAAAGGT

>oqxR-17 ATTTGAGGGCTACCGCAGATTGCTTCAAAAAATTCCCGTATTTGTTATATTGTTGTTCATTGATTATTCACCTCTGCGGTGCCAAAAAGAACAAGATTCACCGCAACCCAGGACACTGCCATGTTAGATTACCGCTTCCCGACAGCTTTGCAGATGGTTCTCAGCGTAGCAATGGCGGAGCAGATGGGTGAACGTTCGACGAGTGCGATTCTGGCCTACGGCCTGGAAGCGAACCCGAGCTTTATCCGTAAACTAATGGTTCCGCTAACTCGTGACGGCATTATCGTCTCCACGCTTGGCCGCAACGGCTCAATTCATCTTGGCCGTCCGGCGGACAAGATCACCCTGCGTGATATCTATCTTTCGGTTATCGAAGATAAAAAACTGTGGGCGTCGCGTCCTGACGTCCCGGCCCGCTGCGTGGTCAGCGCCAACGCCTGCTGGTACTTCAAATCGGTTGCCGATGAAGCAGAGCAGGCTTCGTTAAACGTCCTCGCTCGCCATACCGTGGCCAGCGCGCTGGAGGCGGTCAAAAACGCCGATACCAGCGGCTGCGACCCGGTGCCGGAAATGATCGCCCGCTTTAAAAAAGCGCATTAATCTTTTTTGGGGGGAAAAAAG

>oqxR-18 TATCGAGGGCTACCGCAGATTGCTTCAAAAAATTCCCGTATTTGTTATATTGTTGTTCATTGATTATTCACCTCTGCGGTGCCAAAAAGAACAAGATTCACCGCAACCCAGGACACTGCCATGTTAGATTACCGCTTCCCGACAGCTTTGCAGATGGTTCTCAGCGTAGCAATGGCGGAGCAGATGGGTGAACGTTCGACGAGTGCGATTCTGGCCTACGGCCTGGAAGCGAACCCGAGCTTTATCCGTAAACTAATGGTTCCGCTAACTCGTGACGGCATTATCGTCTCCACGCTTGGCCGCAACGGCTCAATTCATCTTGGCCGTCCGGCGGACAAGATCACCCTGCGTGATATCTATCTTTCGGTTATCGAAGATAAAAAACTGTGGGCGTCGCGTCCTGACGTCCCGGCCCGCTGCGTGGTCAGCGCCAACGCCTGCTGGTACTTCAAATCGGTTGCCGATGAAGCAGAGCAGGCTTCGTTAAACGTCCTCGCTCGCCATACCGTGGCCAGCGCGCTGGAGGCGGTCAAAAACGCCGATACCAGCGGCTGCGACCCGGTGCCGGAAATGATCGCCCGCTTTAAAAAAGCGCATTAATCTTTTTTGGGGGGGAAAAAAG

>oqxR-22 TTTCGCGGCTACCGCAGATTGCTTCAAAAAATTCCCGTATTTGTTATATTGTTGTTCATTGATTATTCACCTCTGCGGTGCCAAAAAGAACAAGATTCACCGCAACCCAGGACACTGCCATGTTAGATTACCGCTTCCCGACAGCTTTGCAGATGGTTCTCAGCGTAGCAATGGCGGAGCAGATGGGTGAACGTTCGACGAGTGCGATTCTGGCCTACGGCCTGGAAGCGAACCCGAGCTTTATCCGTAAACTAATGGTTCCGCTAACTCGTGACGGCATTATCGTCTCCACGCTTGGCCGCAACGGCTCAATTCATCTTGGCCGTCCGGCGGACAAGATCACCCTGCGTGATATCTATCTTTCGGTTATCGAAGATAAAAAACTGTGGGCGTCGCGTCCTGACGTCCCGGCCCGCTGCGTGGTCAGCGCCAACGCCTGCTGGTACTTCAAATCGGTTGCCGATGAAGCAGAGCAGGCTTCGTTAAACGTCCTCGCTCGCCATACCGTGGCCAGCGCGCTGGAGGCGGTCAAAAACGCCGATACCAGCGGCTGCGACCCGGTGCCGGAAATGATCGCCCGCTTTAAAAAAGCGCATTAATCTTTTCTTGGGTGACAAAAA

>oqxR-19 TTTTTGAAGGCTACCGCAGATTGCTTCAAAAAATTCCCGTATTTGTTATATTGTTGTTCATTGATTATTCACCTCTGCGGTGCCAAAAAGAACAAGATTCACCGCAACCCAGGACACTGCCATGTTAGATTACCGCTTCCCGACAGCTTTGCAGATGGTTCTCAGCGTAGCAATGGCGGAGCAGATGGGTGAACGTTCGACGAGTGCGATTCTGGCCTACGGCCTGGAAGCGAACCCGAGCTTTATCCGTAAACTAATGGTTCCGCTAACTCGTGACGGCATTATCGTCTCCACGCTTGGCCGCAACGGCTCAATTCATCTTGGCCGTCCGGCGGACAAGATCACCCTGCGTGATATCTATCTTTCGGTTATCGAAGATAAAAAACTGTGGGCGTCGCGTCCTGACGTCCCGGCCCGCTACGTGGTCAGCGCCAACGCCTGCTGGTACTTCAAATCGGTTGCCGATGAAGCAGAGCAGGCTTCGTTAAACGTCCTCGCTCGCCATACCGTGGCCAGCGCGCTGGAGGCGGTCAAAAACGCCGATACCAGCGGCTGCGACCCGGTACCGGAAATGATCGCCCGCTTTAAAAAAGCGCATTAATCTTTTTGGGGGGGAAAAAA

>oqxR-24 ATAGACGGCTACCGCAGATTGCTTCAAAAAATTCCCGTATTTGTTATATTGTTGTTCATTGATTATTCACCTCTGCGGTGCCAAAAAGAACAAGATTCACCGCAACCCAGGACACTGCCATGTTAGATTACCGCTTCCCGACAGCTTTGCAGATGGTTCTCAGCGTAGCAATGGCGGAGCAGATGGGTGAACGTTCGACGAGTGCGATTCTGGCCTACGGCCTGGAAGCGAACCCGAGCTTTATCCGTAAACTAATGGTTCCGCTAACTCGTGACGGCATTATCGTCTCCACGCTTGGCCGCAACGGCTCAATTCATCTTGGCCGTCCGGCGGACAAGATCACCCTGCGTGATATCTATCTTTCGGTTATCGAAGATAAAAAACTGTGGGCGTCGCGTCCTGACGTCCCGGCCCGCTGCGTGGTCAGCGCCAACGCCTGCTGGTACTTCAAATCGGTTGCCGATGAAGCAGAGCAGGCTTCGTTAAACGTCCTCGCTCGCCATACCGTGGCCAGCGCGCTGGAGGCGGTCAAAAACGCCGATACCAGCGGCTGCGACCCGGTGCCGGAAATGATCGCCCGCTTTAAAAAAGCGCATTAATCTTTTTTGGGGGGAAAAAAG

>oqxR-25 CTTCGAGGCTACCGCAGATTGCTTCAAAAAATTCCCGTATTTGTTATATTGTTGTTCATTGATTATTCACCTCTGCGGTGCCAAAAAGAACAAGATTCACCGCAACCCAGGACACTGCCATGTTAGATTACCGCTTCCCGACAGCTTTGCAGATGGTTCTCAGCGTAGCAATGGCGGAGCAGATGGGTGAACGTTCGACGAGTGCGATTCTGGCCTACGGCCTGGAAGCGAACCCGAGCTTTATCCGTAAACTAATGGTTCCGCTAACTCGTGACGGCATTATCGTCTCCACGCTTGGCCGCAACGGCTCAATTCATCTTGGCCGTCCGGCGGACAAGATCACCCTGCGTGATATCTATCTTTCGGTTATCGAAGATAAAAAACTGTGGGCGTCGCGTCCTGACGTCCCGGCCCGCTGCGTGGTCAGCGCCAACGCCTGCTGGTACTTCAAATCGGTTGCCGATGAAGCAGAGCAGGCTTCGTTAAACGTCCTCGCTCGCCATACCGTGGCCAGCGCGCTGGAGGCGGTCAAAAACGCCGATACCAGCGGCTGCGACCCGGTGCCGGAAATGATCGCCCGCTTTAAAAAAGCGCATTAATCTTTTTGGGGGGGAAAAAAGTTG

>oqxR-26 GGGGGGAAACGCAGATTGCTTCAAAAAATTCCCGTATTTGTTATATTGTTGTTCATTGATTATTCACCTCTGCGGTGCCAAAAAGAACAAGATTCACCGCAACCCAGGACACTGCCATGTTAGATTACCGCTTCCCGACAGCTTTGCAGATGGTTCTCAGCGTAGCAATGGCGGAGCAGATGGGTGAACGTTCGACGAGTGCGATTCTGGCCTACGGCCTGGAAGCGAACCCGAGCTTTATCCGTAAACTAATGGTTCCGCTAACTCGTGACGGCATTATCGTCTCCACGCTTGGTCGCAACGGCTCAATTCATCTTGGCCGTCCGGCGGACAAGATCACCCTGCGTGATATCTATCTTTCGGTTATCGAAGATAAAAAACTGTGGGCGTCGCGTCCTGACGTCCCGGCCCGCTGCGTGGTCAGCGCCAACGCCTGCTGGTACTTCAAATCGGTTGCCGATGAAGCAGAGCAGGCTTCGTTAAACGTCCTCGCTCGCCATACCGTGGCCAGCGCGCTGGAGGCGGTCAAAAACGCCGATACCAGCGGCTGCGATCCGGTGCCGGAAATGATCGCCCGCTTTAAAAAAGCGCATTAATCTTTTTTGGGGGAAAAAA

>oqxR-27 CTTTCGAGGCTACCGCAGATTGCTTCAAAAAATTCCCGTATTTGTTATATTGTTGTTCATTGATTATTCACCTCTGCGGTGCCAAAAAGAACAAGATTCACCGCAACCCAGGACACTGCCATGTTAGATTACCGCTTCCCGACAGCTTTGCAGATGGTTCTCAGCGTAGCAATGGCGGAGCAGATGGGTGAACGTTCGACGAGTGCGATTCTGGCCTACGGCCTGGAAGCGAACCCGAGCTTTATCCGTAAACTAATGGTTCCGCTAACTCGTGACGGCATTATCGTCTCCACGCTTGGCCGCAACGGCTCAATTCATCTTGGCCGTCCGGCGGACAAGATCACCCTGCGTGATATCTATCTTTCGGTTATCGAAGATAAAAAACTGTGGGCGTCGCGTCCTGACGTCCCGGCCCGCTGCGTGGTCAGCGCCAACGCCTGCTGGTACTTCAAATCGGTTGCCGATGAAGCAGAGCAGGCTTCGTTAAACGTCCTCGCTCGCCATACCGTGGCCAGCGCGCTGGAGGCGGTCAAAAACGCCGATACCAGCGGCTGCGACCCGGTGCCGGAAATGATCGCCCGCTTTAAAAAAGCGCATTAATCTTTTTGGGGGGGAAAAAAGGC

>oqxR-28 CTTTCCAGGCTACCGCAGATTGCTTCAAAAAATTCCCGTATTTGTTATATTGTTGTTCATTGATTATTCACCTCTGCGGTGCCAAAAAGAACAAGATTCACCGCAACCCAGGACACTGCCATGTTAGATTACCGCTTCCCGACAGCTTTGCAGATGGTTCTCAGCGTAGCAATGGCGGAGCAGATGGGTGAACGTTCGACGAGTGCGATTCTGGCCTACGGCCTGGAAGCGAACCCGAGCTTTATCCGTAAACTAATGGTTCCGCTAACTCGTGACGGCATTATCGTCTCCACGCTTGGCCGCAACGGCTCAATTCATCTTGGCCGTCCGGCGGACAAGATCACCCTGCGTGATATCTATCTTTCGGTTATCGAAGATAAAAAACTGTGGGCGTCGCGTCCTGACGTCCCGGCCCGCTGCGTGGTCAGCGCCAACGCCTGCTGGTACTTCAAATCGGTTGCCGATGAAGCAGAGCAGGCTTCGTTAAACGTCCTCGCTCGCCATACCGTGGCCAGCGCGCTGGAGGCGGTCAAAAACGCCGATACCAGCGGCTGCGACCCGGTGCCGGAAATGATCGCCCGCTTTAAAAAAGCGCATTAATCTTTTTTGGGGGGGAAAAAATT

>oqxR-29 TTTCGAAGGCTACCGCAGATTGCTTCAAAAAATTCCCGTATTTGTTATATTGTTGTTCATTGATTATTCACCTCTGCGGTGCCAAAAAGAACAAGATTCACCGCAACCCAGGACACTGCCATGTTAGATTACCGCTTCCCGACAGCTTTGCAGATGGTTCTCAGCGTAGCAATGGCGGAGCAGATGGGTGAACGTTCGACGAGTGCGATTCTGGCCTACGGCCTGGAAGCGAACCCGAGCTTTATCCGTAAACTAATGGTTCCGCTAACTCGTGACGGCATTATCGTCTCCACGCTTGGCCGCAACGGCTCAATTCATCTTGGCCGTCCGGCGGACAAGATCACCGCGTGATATCTATCTTTCGGTTATCGAAGATAAAAAACTGTGGGCGTCGCGTCCTGACGTCCCGGCCCGCTGCGTGGTCAGCGCCAACGCCTGCTGGTACTTCAAATCGGTTGCCGATGAAGCAGAGCAGGCTTCGTTAAACGTCCTCGCTCGCCATACCGTGGCCAGCGCGCTGGAGGCGGTCAAAAACGCCGATACCAGCGGCTGCGACCCGGTGCCGGAAATGATCGCCCGCTTTAAAAAAGCGCATTAATCATTTTTTGGGGGGGAAAAAAGCAG

>oqxR-30 CCCGTAGATTGCTTCAAAAAATTCCCGTATTTGTTATATTGTTGTTCATTGATTATTCACCTCTGCGGTGCCAAAAAGAACAAGATTCACCGCAACCCAGGACACTGCCATGTTAGATTACCGCTTCCCGACAGCTTTGCAGATGGTTCTCAGCGTAGCAATGGCGGAGCAGATGGGTGAACGTTCGACGAGTGCGATTCTGGCCTACGGCCTGGAAGCGAACCCGAGCTTTATCCGTAAACTAATGGTTCCGCTAACTCGTGACGGCATTATCGTCTCCACGCTTGGCCGCAACGGCTCAATTCATCTTGGCCGTCCGGCGGACAAGATCACCCTGCGTGATATCTATCTTTCGGTTATCGAAGATAAAAAACTGTGGGCGTCGCGTCCTGACGTCCCGGCCCGCTGCGTGGTCAGCGCCAACGCCTGCTGGTACTTCAAATCGGTTGCCGATGAAGCAGAGCAGGCTTCGTTAAACGTCCTCGCTCGCCATACCGTGGCCAGCGCGCTGGAGGCGGTCAAAAACGCCGATACCAGCGGCTGCGACCCGGTGCCGGAAATGATCGCCCGCTTTAAAAAAGCGCATTAATCTTTTTTGGGGGGGAAAAAAAATCGTT

>ramR-1 CTTCCTGGGCCGCAGTGTTCGGTAACGGGTAGGTCAGGGCGATACGGTGAGCGCAGGGATGCAGTGTTTCCGGCGTCATTAGGCGTCCGCCTCATGCAGGGCGTGCCACATGGCTTCAAAGCCGAGGGCGATAATCTCCCGGGCGCGCTGCGGATCGTGGCTGGCGAATTCGATGGTGGTTTCCGCCAGCGACAGAAACAGGGCGTCGCCAAAGGCGCGGTACGCCTCGCTGAGGAATATCTCTTTCACCGACAGCTGGCACATTTCGTTGAGCTCCGGAAAGCTCTCTTTTACCTGGCGGCGGGTTTCGTCGGTGATGCGCTCGCTGAGCGCCATCCGGCGGATCGCTTTGTGCTCCATCGGGTTGCGCACGCCCCAGTCGATATAGCTGTTCCAGATATTGCGCGCGTTCTCTTTCGGGCGCTTCTCGTCCGGATCCAGCCCGGCGATCATTGTGCGCACCAGGCGCAGCTTAATCGCGAGGTACAGCTCGTTGAGCAACTCATCCTTGGTGGCGAAATAGCGAAACAGCGTTCCCTCGGCCACACCGGCGCTGCGGGCGATGGCCGACGTCGAGGCGGCTATGCCGGACTGGGCGAAAGCCGCGGTGGCAGCTTCCAGTAACGCTTGCTTTTTATCTTCACTCTTTGGACGAGCCACTACTTTTTTCCTCACGCAGGTTTAAACAAACCGCCGATCTTGGCACGTCTGACCAGGTCGCTGCAACGGCGGAACGCAAAAATTGAAAATCGTCTTGACGACTTTCATCGCTTTCCTAATAATGAGTGCGTCTCACTCATAATCAAGTTATATCATAAAGCCTCCGGATGGGGCTTTGCTGAAAAGAAAAAAAAACAAGAGCGCGCGGGGGAGGCCGGAA

>ramR-2 TCAAATGGCGCAGTGTTCGGTAACGGGTAGGTCAGGGCGATACGGTGAGCGCAGGGATGCAGTGTTTCCGGCGTCATTATGCGTCCGCCTCATGCAGGGCGTGCCACATGGCTTCAAAGCCGAGGGCGATAATCTCCCGGGCGCGCTGCGGATCGTGGCTGGCGAATTCGATGGTGGTTTCCGCCAGCGACAGAAACAGGGCGTCGCCAAAGGCGCGGTACGCCTCGCTGAGGAATATCTCTTTCACCGACAGCTGGCACATTTCGTTGAGCTCCGGAAAGCTCTCTTTTACCTGGCGGCGGGTTTCGTCGGTGATGCGCTCGCTGAGCGCCATCCGGCGGATCGCTTTGTGCTCCATCGGGTTGCGCACGCCCCAGTCGATATAGCTGTTCCAGATATTGCGCGCGTTCTCTTTCGGGCGCTTCTCGTCCGGATCCAGCCCGGCGATCATTGTGCGCACCAGGCGCAGCATAATCGCGAGGTACAGCTCGTTGAGCAACTCATCCTTGGTGGCGAAATAGCGAAACAGCGTTCCCTCGGCCACACCGGCGCTGCGGGCGATGGCCGACGTCGAGGCGGCTATGCCGGACTGGGCGAAAGCCACGGTGGCAGCTTCCAGTAACGCTTGCTTTTTATCTTCACTCTTTGGACGAGCCACTACTTTTTTCCTCACGCAGGTTTAAACAAACCGCCGATCTTGGCACGTCTGACCAGGTCGCTGCAACGGCGGAACGCAAAAATTGAAAATCGTCTTGACGACTTTCATCGCTTTCCTAATAATGAGTGCGTACTCACTCATAATCAAGTTATATCATAAAGCCTCCGGGATGGGGCTTTGCTGGGGAAAAAAAAAAAACCCGCGCGCGGGGG

>ramR-3 GCGTCAGTTGCAGTGTTCGGTAACGGGTAGGTCAGGGCGATACGGTGAGCGCAGGGATGCAGTGTTTCCGGCGTCATTATGCGTCCGCCTCATGCAGGGCGTGCCACATGGCTTCAAAGCCGAGGGCGATAATCTCCCGGGCGCGCTGCGGATCGTGGCTGGCGAATTCGATGGTGGTTTCCGCCAGCGACAGAAACAGGGCGTCGCCAAAGGCGCGGTACGCCTCGCTGAGGAATATCTCTTTCACCGACAGCTGGCACATTTCGTTGAGCTCCGGAAAGCTCTCTTTTACCTGGCGGCGGGTTTCGTCGGTGATGCGCTCGCTGAGCGCCATCCGGCGGATCGCTTTGTGCTCCATCGGGTTGCGCACGCCCCAGTCGATATAGCTGTTCCAGATATTGCGCGCGTTCTCTTTCGGGCGCTTCTCGTCCGGATCCAGCCCGGCGATCATTGTGCGCACCAGGCGCAGCATAATCGCGAGGTACAGCTCGTTGAGCAACTCATCCTTGGTGGCGAAATAGCGAAACAGCGTTCCCTCGGCCACACCGGCGCTGCGGGCGATGGCCGACGTCGAGGCGGCTATGCCGGACTGGGCGAAAGCCACGGTGGCAGCTTCCAGTAACGCTTGCTTTTTATCTTCACTCTTTGGACGAGCCACTACTTTTTTCCTCACGCAGGTTTAAACAAACCGCCGATCTTGGCACGTCTGACCAGGTCGCTGCAACGGCGGAACGCAAAAATTGAAAATCGTCTTGACGACTTTCATCGCTTTCCTAATAATGAGTGCGTACTCACTCATAATCAAGTTATATCATAAAGCCTCCGGATGGGGCTTTGTGGAAAAAAAAAAAAAGAGAGAGGACAAGGGGG

>ramR-5 TCGAGCTGGCTAGCAGTGTTCGGTAACGGGTAGGTCAGGGCGATACGGTGAGCGCAGGGATGCAGTGTTTCCGGCGTCATTATGCGTCCGCCTCATGCAGGGCGTGCCACATGGCTTCAAAGCCGAGGGCGATAATCTCCCGGGCGCGCTGCGGATCGTGGCTGGCGAATTCGATGGTGGTTTCCGCCAGCGACAGAAACAGGGCGTCGCCAAAGGCGCGGTACGCCTCGCTGAGGAATATCTCTTTCACCGACAGCTGGCACATTTCGTTGAGCTCCGGAAAGCTCTCTTTTACCTGGCGGCGGGTTTCGTCGGTGATGCGCTCGCTGAGCGCCATCCGGCGGATCGCTTTGTGCTCCATCGGGTTGCGCACGCCCCAGTCGATATAGCTGTTCCAGATATTGCGCGCGTTCTCTTTCGGGCGCTTCTCGTCCGGATCCAGCCCGGCGATCATTGTGCGCACCAGGCGCAGCTTAATCGCGAGGTACAGCTCGTTGAGCAACTCATCCTTGGTGGCGAAATAGCGAAACAGCGTTCCCTCGGCCACACCGGCGCTGCGGGCGATGGCCGACGTCGAGGCGGCTATGCCGGACTGGGCGAAAGCCGCGGTGGCAGCTTCCAGTAACGCTTGCTTTTTATCTTCACTCTTTGGACGAGCCACTACTTTTTTCCTCACGCAGGTTTAAACAAACCGCCGATCTTGGCACGTCTGACCAGGTCGCTGCAACGGCGGAACGCAAAAATTGAAAATCGTCTTGACGACTTTCATCGCTTTCCTAATAATGAGTGCGTACTCACTCATAATCAAGTTATATCATAAAGCCTCCGGGATGGGGGCTTTTGTCTGGCAGAAAATATAAACCGTGGACTGC

>ramR-7 CCGTTCTGGCGCAGTGTTCGGTAACGGGTAGGTCAGGGCGATACGGTGAGCGCAGGGATGCAGTGTTTCCGGCGTCATTATGCGTCCGCCTCATGCAGGGCGTGCCACATGGCTTCAAAGCCGAGGGCGATAATCTCCCGGGCGCGCTGCGGATCGTGGCTGGCGAATTCGATGGTGGTTTCCGCCAGCGACAGAAACAGGGCGTCGCCAAAGGCGCGGTACGCCTCGCTGAGGAATATCTCTTTCACCGACAGCTGGCACATTTCGTTGAGCTCCGGAAAGCTGGCGGCGGGTTTCGTCGGTGATGCGCTCGCTGAGCGCCATCCGGCGGATCGCTTTGTGCTCCATCGGGTTGCGCACGCCCCAGTCGATATAGCTGTTCCAGATATTGCGCGCGTTCTCTTTCGGGCGCTTCTCGTCCGGATCCAGCCCGGCGATCATTGTGCGCACCAGGCGCAGCTTAATCGCGAGGTACAGCTCGTTGAGCAACTCATCCTTGGTGGCGAAATAGCGAAACAGCGTTCCCTCGGCCACACCGGCGCTGCGGGCGATGGCCGACGTCGAGGCGGCTATGCCGGACTGGGCGAAAGCCGCGGTGGCAGCTTCCAGTAACGCTTGCTTTTTATCTTCACTCTTTGGACGAGCCACTACTTTTTTCCTCACGCAGGTTTAAACAAACCGCCGATCTTGGCACGTCTGACCAGGTCGCTGCAACGGCGGAACGCAAAAATTGAAAATCGTCTTGACGACTTTCATCGCTTTCCTAATAATGAGTGCGTACTCACTCATAATCAAGTTATATCATAAAGCCTCCGGGATGGGGGCTTTGCTGCGAAAAATATAA

>ramR-8 TCCGTCAGGTAGCAGTGTTCGGTAACGGGTAGGTCAGGGCGATACGGTGAGCGCAGGGATGCAGTGTTTCCGGCGTCATTATGCGTCCGCCTCATGCAGGGCGTGCCACATGGCTTCAAAGCCGAGGGCGATAATCTCCCGGGCGCGCTGCGGATCGTGGCTGGCGAATTCGATGGTGGTTTCCGCCAGCGACAGAAACAGGGCGTCGCCAAAGGCGCGGTACGCCTCGCTGAGGAATATCTCTTTCACCGACAGCTGGCACATTTCGTTGAGCTCCGGAAAGCTCTCTTTTACCTGGCGGCGGGTTTCGTCGGTGATGCGCTCGCTGAGCGCCATCCGGCGGATCGCTTTGTGCTCCATCGGGTTGCGCACGCCCCAGTCGATATAGCTGTTCCAGATATTGCGCGCGTTCTCTTTCGGGCGCTTCTCGTCCGGATCCAGCCCGGCGATCATTGTGCGCACCAGGCGCAGCTTAATCGCGAGGTACAGCTCGTTGAGCAACTCATCCTTGGTGGCGAAATAGCGAAACAGCGTTCCCTCGGCCACACCGGCGCTGCGGGCGATGGCCGACGTCGAGGCGGCTATGCCGGACTGGGCGAAAGCCGCGGTGGCAGCTTCCAGTAACGCTTGCTTTTTATCTTCACTCTTTGGACGAGCCACTACTTTTTTCCTCACGCAGGTTTAAACAAACCGCCGATCTTGGCACGTCTGACCAGGTCGCTGCAACGGCGGAACGCAAAAATTGAAAATCGTCTTGACGACTTTCATCGCTTTCCTAATAATGAGTGCGTACTCACTCATAATCAAGTTATATCATAAAGCCTCCGGGATGGGGGTTTTGCTGCGAAAAATAAA

>ramR-9 GTGAAACCCAACAGACCCCTGATCGTAATTCTGAGCACTGTCGCGCTCGACGCTGTCGGCATCGGCCTGATTATGCCGGTGCTGCCGGGCCTCCTGCGCGATCTGGTTCACTCGAACGACGTCgCCGCCCACTATGGCATTCTGCTGGCGCTGTATGCGTTGATGCAATTTGCCTGCGCACCTGTGCTGGGCGCGCTGTCGGATCGTTTCGGGCGGCGGCCGGTCTTGCTCGTCTCGCTGGCCGGCGCTGCTGTCGACTACGCCATCATGGCGACGGCGCCTTTCCTTTGGGTTCTCTATATCGGGCGGATCGTGGCCGGCATCACCGGGGCGACTGGGGCGGTAGCCGGCGCTTATATTGCCGATATCACTGATGGCGATGAGCGCGCGCGGCACTTCGGCTTCATGAGCGCCTGTTTCGGGTTCGGGATGGTCGCGGGACCTGTGCTCGGTGGGCTGATGGGCGGTTTCTCCCCCCACGCTCCGTTCTTCGCCGCGGCAGCCTTGAACGGCCTCAATTTCCTGACGGGCTGTTTCCTTTTGCCGGAGTCGCACAAAGGCGAACGCCGGCCGTTACGCCGGGAGGCTCTCAACCCGCTCGCTTCGTTCCGGTGGGCCCGGGGCATGACCGTCGTCGCCGCCCTGATGGCGGTCTTCTTCATCATGCAACTTGTCGGACAGGTGCCGGCCGCGCTTTGGGTCATTTTCGGCGAGGATCGCTTTCACTGGGACGCGACCACGATCGGCATTTCGCTTGCCGCATTTGGCATTCTGCATTCACTCGCCCAGGCAATGATCACCGGCCCTGTAGCCGCCCGGCTCGGCGAAAGGCGGGCACTCATGCTCGGAATGATTGCCGACGGCACAGGCTACATCCTGCTTGCCTTCGCGACACGGGGATGGATGGCGTTCCCGATCATGGTCCTGCTTGCTTCGGGTGGCATCGGAATGCCGGCGCTGCAAGCAATGTTGTCCAGGCAGGTGGATGAGGAACGTCAGGGGCAGCTGCAAGGCTCACTGGCGGCGCTCACCAGCCTGACCTCGATCGTCGGACCCCTCCTCTTCACGGCGATCTATGCGGCTTCTATAACAACGTGGAACGGGTGGGCATGGATTGCAGGCGCTGCCCTCTACTTGCTCTGCCTGCCGGCGCTGCGTCGCGGGCTTTGGAGCGGCGCAGGGCAACGAGCCGATCGCTGA

>ramR-10 TCGTTCTGCTAGCAGTGTTCGGTAACGGGTAGGTCAGGGCGATACGGTGAGCGCAGGGATGCAGTGTTTCCGGCGTCATTAGGCGTCCGCCTCATGCAGGGCGTGCCACATGGCTTCAAAGCCGAGGGCGATAATCTCCCGGGCGCGCTGCGGATCGTGGCTGGCGAATTCGATGGTGGTTTCCGCCAGCGACAGAAACAGGGCGTCGCCAAAGGCGCGGTACGCCTCGCTGAGGAATATCTCTTTCACCGACAGCTGGCACATTTCGTTGAGCTCCGGAAAGCTCTCTTTTACCTGGCGGCGGGTTTCGTCGGTGATGCGCTCGCTGAGCGCCATCCGGCGGATCGCTTTGTGCTCCATCGGGTTGCGCACGCCCCAGTCGATATAGCTGTTCCAGATATTGCGCGCGTTCTCTTTCGGGCGCTTCTCGTCCGGATCCAGCCCGGCGATCATTGTGCGCACCAGGCGCAGCTTAATCGCGAGGTACAGCTCGTTGAGCAACTCATCCTTGGTGGCGAAATAGCGAAACAGCGTTCCCTCGGCCACACCGGCGCTGCGGGCGATGGCCGACGTCGAGGCGGCTATGCCGGACTGGGCGAAAGCCGCGGTGGCAGCTTCCAGTAACGCTTGCTTTTTATCTTCACTCTTTGGACGAGCCACTACTTTTTTCCTCACGCAGGTTTAAACAAACCGCCGATCTTGGCACGTCTGACCAGGTCGCTGCAACGGCGGAACGCAAAAATTGAAAATCGTCTTGACGACTTTCATCGCTTTCCTAATAATGAGTGCGTACTCACTCATAATCAAGTTATATCATAAAGCCTCCGGGATGGGGGCTTTGCTGCAGAAAAATAAAACACCGCCGGGGGATTTTAAA

>ramR-11 CGGTAAGGGTAGCAGTGTTCGGTAACGGGTAGGTCAGGGCGATACGGTGAGCGCAGGGATGCAGTGTTTCCGGCGTCATTATGCGTCCGCCTCATGCAGGGCGTGCCACATGGCTTCAAAGCCGAGGGCGATAATCTCCCGGGCGCGCTGCGGATCGTGGCTGGCGAATTCGATGGTGGTTTCCGCCAGCGACAGAAACAGGGCGTCGCCAAAGGCGCGGTACGCCTCGCTGAGGAATATCTCTTTCACCGACAGCTGGCACATTTCGTTGAGCTCCGGAAAGCTCTCTTTTACCTGGCGGCGGGTTTCGTCGGTGATGCGCTCGCTGAGCGCCATCCGGCGGATCGCTTTGTGCTCCATCGGGTTGCGCACGCCCCAGTCGATATAGCTGTTCCAGATATTGCGCGCGTTCTCTTTCGGGCGCTTCTCGTCCGGATCCAGCCCGGCGATCATTGTGCGCACCAGGCGCAGCTTAATCGCGAGGTACAGCTCGTTGAGCAACTCATCCTTGGTGGCGAAATAGCGAAACAGCGTTCCCTCGGCCACACCGGCGCTGCGGGCGATGGCCGACGTCGAGGCGGCTATGCCGGACTGGGCGAAAGCCGCGGTGGCAGCTTCCAGTAACGCTTGCTTTTTATCTTCACTCTTTGGACGAGCCACTACTTTTTTCCTCACGCAGGTTTAAACAAACCGCCGATCTTGGCACGTCTGACCAGGTCGCTGCAACGGCGGAACGCAAAAATTGAAAATCGTCTTGACGACTTTCATCGCTTTCCTAATAATGAGTGCGTACTCACTCATAATCAAGTTATATCATAAAGCCTCCGGGATGGGGGTTTTGTCGGCGAAAAAAAAAAAAACGCGCGGGAAAGA

>ramR-12 GCTGTCTGGTTAGCAGTGTTCGGTAACGGGTAGGTCAGGGCGATACGGTGAGCGCAGGGATGCAGTGTTTCCGGCGTCATTAGGCGTCCGCCTCATGCAGGGCGTGCCACATGGCTTCAAAGCCGAGGGCGATAATCTCCCGGGCGCGCTGCGGATCGTGGCTGGCGAATTCGATGGTGGTTTCCGCCAGCAACAGAAACAGGGCGTCGCCAAAGGCGCGGTACGCCTCGCTGAGGAATATCTCTTTCACCGACAGCTGGCACATTTCGTTGAGCTCCGGAAAGCTCTCTTTTACCTGGCGGCGGGTTTCGTCGGTGATGCGCTCGCTGAGCGCCATCCGGCGGATCGCTTTGTGCTCCATCGGGTTGCGCACGCCCCAGTCGATATAGCTGTTCCAGATATTGCGCGCGTTCTCTTTCGGGCGCTTCTCGTCCGGATCCAGCCCGGCGATCATTGTGCGCACCAGGCGCAGCTTAATCGCGAGGTACAGCTCGTTGAGCAACTCATCCTTGGTGGCGAAATAGCGAAACAGCGTTCCCTCGGCCACACCGGCGCTGCGGGCGATGGCCGACGTCGAGGCGGCTATGCCGGACTGGGCGAAAGCCGCGGTGGCAGCTTCCAGTAACGCTTGCTTTTTATCTTCACTCTTTGGACGAGCCACTACTTTTTTCCTCACGCAGGTTTAAACAAACCGCCGATCTTGGCACGTCTGACCAGGTCGCTGCAACGGCGGAACGCAAAAATTGAAAATCGTCTTGACGACTTTCATCGCTTTCCTAATAATGAGTGCGTACTCACTCATAATCAAGTTATATCATAAAGCCTCCGGGATGGGGGTTTTGCTGCGAAAAAAAAAAAAACACCGGGGGAACC

>ramR-13 TCTTATAATGCTGCAGTGTTCGGTAACGGGTAGGTCAGGGCGATACGGTGAGCGCAGGGATGCAGTGTTTCCGGCGTCATTAGGCGTCCGCCTCATGCAGGGCGTGCCACATGGCTTCAAAGCCGAGGGCGATAATCTCCCGGGCGCGCTGCGGATCGTGGCTGGCGAATTCGATGGTGGTTTCCGCCAGCGACAGAAACAGGGCGTCGCCAAAGGCGCGGTACGCCTCGCTGAGGAATATCTCTTTCACCGACAGCTGGCACATTTCGTTGAGCTCCGGAAAGCTCTCTTTTACCTGGCGGCGGGTTTCGTCGGTGATGCGCTCGCTGAGCGCCATCCGGCGGATCGCTTTGTGCTCCATCGGGTTGCGCACGCCCCAGTCGATATAGCTGTTCCAGATATTGCGCGCGTTCTCTTTCGGGCGCTTCTCGTCCGGATCCAGCCCGGCGATCATTGTGCGCACCAGGCGCAGCTTAATCGCGAGGTACAGCTCGTTGAGCAACTCATCCTTGGTGGCGAAATAGCGAAACAGCGTTCCCTCGGCCACACCGGCGCTGCGGGCGATGGCCGACGTCGAGGCGGCTATGCCGGACTGGGCGAAAGCCGCGGTGGCAGCTTCCAGTAACGCTTGCTTTTTATCTTCACTCTTTGGACGACCCACTACTTTTTTCCTCACGCAGGTTTAAACAAACCGCCGATCTTGGCACGTCTGACCAGGTCGCTGCAACGGCGGAACGCAAAAATTGAAAATCGTCTTGACGACTTTCATCGCTTTCCTAATAATGAGTGCGTACTCACTCATAATCAAGTTATATCATAAAGCCTCCGGGATGGGGCTTTTGCTGCGAAAAAAAGAAAACGCGCGGGGGATTTCAAAA

>ramR-14 CGTATATGGGCGCAGTGTTCGGTAACGGGTAGGTCAGGGCGATACGGTGAGCGCAGGGATGCAGCATCTCAGGGGTCATTTGGCGTCCGCCTCATGCAGGGCGTGCCACATGGCTTCAAAGCCGAGGGCGATAATCTCCCGGGCGCGCTGCGGATCGTGGCTGGCGAATTCGATGGTGGTTTCCGCCAGCGACAGAAACAGGGCGTCGCCAAAGGCGCGGTACGCCTCGCTGAGGAATATCTCTTTCACCGACAGCTGGCACATTTCGTTGAGCTCCGGAAAGCTCTCTTTTACCTGGCGGCGGGTTTCGTCGGTGATGCGCTCGCTGAGCGCCATCCGGCGGATCGCTTTGTGCTCCATCGGGTTGCGCACGCCCCAGTCGATATAGCTGTTCCAGATATTGCGCGCGTTCTCTTTCGGGCGCTTCTCGTCCGGATCCAGCCCGGCGATCATTGTGCGCACCAGGCGCAGCTTAATCGCGAGGTACAGCTCGTTGAGCAACTCATCCTTGGTGGCGAAATAGCGAAACAGCGTTCCCTCGGCCACACCGGCGCTGCGGGCGATGGCCGACGTCGAGGCGGCTATGCCGGACTGGGCGAAAGCCGCGGTGGCAGCTTCCAGTAACGCTTGCTTTTTATCTTCACTCTTTGGACGAGCCACTACTTTTTTCCTCACGCAGGTTTAAACAAACCGCCGATCTTGGCACGTCTGACCAGGTCGCTGCAACGGCGGAACGCAAAAATTGAAAATCGTCTTGACGACTTTCATCGCTTTCCTAATAATGAGTGCGTACTCACTCATAATCAAGTTATATCATAAAGCCTCCGGGATGGGGGCTTTTGCTGGAGAAAAAAAAAAAACACGCGGGGGAAGTG

>ramR-15 TCCTCTGGGCGCAGTGTTCGGTAACGGGTAGGTCAGGGCGATACGGTGAGCGCAGGGATGCAGTGTTTCCGGCGTCATTATGCGTCCGCCTCATGCAGGGCGTGCCACATGGCTTCAAAGCCGAGGGCGATAATCTCCCGGGCGCGCTGCGGATCGTGGCTGGCGAATTCGATGGTGGTTTCCGCCAGCGACAGAAACAGGGCGTCGCCAAAGGCGCGGTACGCCTCGCTGAGGAATATCTCTTTCACCGACAGCTGGCACATTTCGTTGAGCTCCGGAAAGCTCTCTTTTACCTGGCGGCGGGTTTCGTCGGTGATGGCGCCATCCGGCGGATCGCTTTGTGCTCCATCGGGTTGCGCACGCCCCAGTCGATATAGCTGTTCCAGATATTGCGCGCGTTCTCTTTCGGGCGCTTCTCGTCCGGATCCAGCCCGGCGATCATTGTGCGCACCAGGCGCAGCTTAATCGCGAGGTACAGCTCGTTGAGCAACTCATCCTTGGTGGCGAAATAGCGAAACAGCGTTCCCTCGGCCACACCGGCGCTGCGGGCGATGGCCGACGTCGAGGCGGCTATGCCGGACTGGGCGAAAGCCGCGGTGGCAGCTTCCAGTAACGCTTGCTTTTTATCTTCACTCTTTGGACGAGCCACTACTTTTTTCCTCACGCAGGTTTAAACAAACCGCCGATCTTGGCACGTCTGACCAGGTCGCTGCAACGGCGGAACGCAAAAATTGAAAATCGTCTTGACGACTTTCATCGCTTTCCTAATAATGAGTGCGTACTCACTCATAATCAAGTTATATCATAAAGCCTCCGGGATGGGGGCTTTGCTGCGAAAAAAAAAAACCCCGCGCGGGGGAACCCAA

>ramR-16 CTCCTTCTCTGTTCGGCAACGGGTAGGTCAGGGCGATACGGTGAGCGCAGGGATGCAGTGTTTCCGGCGTCATTATGCGTCCGCCTCATGCAGGGCGTGCCACATGGCTTCAAAGCCGAGGGCGATAATCTCCCGGGCGCGCTGCGGATCGTGGCTGGCGAATTCGATGGTGGTTTCCGCCAGCGACAGAAACAGGGCGTCGCCAAAGGCGCGGTACGCCTCGCTGAGGAATATCTCTTTCACCGACAGCTGGCACATTTCGTTGAGCTCCGGAAAGCTCTCTTTTACCTGGCGGCGGGTTTCGTCGGTGATGCGCTCGCTGAGCGCCATCCGGCGGATCGCTTTGTGCTCCATCGGGTTGCGCACGCCCCAGTCGATATAGCTGTTCCAGATATTGCGCGCGTTCTCTTTCGGGCGCTTCTCGTCCGGATCCAGCCCGGCGATCATTGTGCGCACCAGGCGCAGCTTAATCGCGAGGTACAGCTCGTTGAGCAACTCATCCTTGGTGGCGAAATAGCGAAACAGCGTTCCCTCGGCCACACCGGCGCTGCGGGCGATGGCCGACGTCGAGGCGGCTATGCCGGACTGGGCGAAAGCCGCGGTGGCAGCTTCCAGTAACGCTTGCTTTTTATCTTCACTCTTTGGACGAGCCACTACTTTTTTCCTCACGCAGGTTTAAACAAACCGCCGATCTTGGCACGTCTGACCAGGTCGCTGCAACGGCGGAACGCAAAAATTGAAAATCGTCTTGACGACTTTCATCGCTTTCCTAATAATGAGTGCGTACTCACTCATAATCAAGTTATATCATAAAGCCTCCGGGATGGGGGCTTTTGTCTGGTCAGAATTATGAACCG

>ramR-17 GCGTCAGCCGCAGTGTTCGGTAACGGGTAGGTCAGGGCGATACGGTGAGCGCAGGGATGCAGTGTTTCCGGCGTCATTATGCGTCCGCCTCATGCAGGGCGTGCCACATGGCTTAAAAGCCGAGGGCGATAATCTCCCGGGCGCGCTGCGGATCGTGGCTGGCGAATTCGATGGTGGTTTCCGCCAGCGACAGAAACAGGGCGTCGCCAAAGGCGCGGTACGCCTCGCTGAGGAATATCTCTTTCACCGACAGCTGGCACATTTCGTTGAGCTCCGGAAAGCTCTCTTTTACCTGGCGGCGGGTTTCGTCGGTGATGCGCTCGCTGAGCGCCATCCGGCGGATCGCTTTGTGCTCCATCGGGTTGCGCACGCCCCAGTCGATATAGCTGTTCCAGATATTGCGCGCGTTCTCTTTCGGGCGCTTCTCGTCCGGATCCAGCCCGGCGATCATTGTGCGCACCAGGCGCAGCTTAATCGCGAGGTACAGCTCGTTGAGCAACTCATCCTTGGTGGCGAAATAGCGAAACAGCGTTCCCTCGGCCACACCGGCGCTGCGGGCGATGGCCGACGTCGAGGCGGCTATGCCGGACTGGGCGAAAGCCGCGGTGGCAGCTTCCAGTAACGCTTGCTTTTTATCTTCACTCTTTGGACGAGCCACTACTTTTTTCCTCACGCAGGTTTAAACAAACCGCCGATCTTGGCACGTCTGACCAGGTCGCTGCAACGGCGGAACGCAAAAATTGAAAATCGTCTTGACGACTTTCATCGCTTTCCTAATAATGAGTGCGTACTCACTCATAATCAAGTTATATCATAAAGCCTTCGGGATGGGGGCTTTGCTGCGAAAAAATATAA

>ramR-18 GCGTTCTGGTGCAGTGTTCGGTAACGGGTAGGTCAGGGCGATACGGTGAGCGCAGGGATGCAGTGTTTCCGGCGTCATTAGGCGTCCGCCTCATGCAGGGCGTGCCACATGGCTTCAAAGCCGAGGGCGATAATCTCCCGGGCGCGCTGCGGATCGTGGCTGGCGAATTCGATGGTGGTTTCCGCCAGCGACAGAAACAGGGCGTCGCCAAAGGCGCGGTACGCCTCGCTGAGGAATATCTCTTTCACCGACAGCTGGCACATTTCGTTGAGCTCCGGAAAGCTCTCTTTTACCTGGCGGCGGGTTTCGTCGGTGATGCGCTCGCTGAGCGCCATCCGGCGGATCGCTTTGTGCTCCATCGGGTTGCGCACGCCCCAGTCGATATAGCTGTTCCAGATATTGCGCGCGTTCTCTTTCGGGCGCTTCTCGTCCGGATCCAGCCCGGCGATCATTGTGCGCACCAGGCGCAGCTTAATCGCGAGGTACAGCTCGTTGAGCAACTCATCCTTGGTGGCGAAATAGCGAAACAGCGTTCCCTCGGCCACACCGGCGCTGCGGACGATGGCCGACGTCGAGGCGGCTATGCCGGACTGGGCGAAAGCCGCGGTGGCAGCTTCCAGTAACGCTTGCTTTTTATCTTCACTCTTTGGACGAGCCACTACTTTTTTCCTCACGCAGGTTTAAACAAACCGCCGATCTTGGCACGTCTGACCAGGTCGCTGCAACGGCGGAACGCAAAAATTGAAAATCGTCTTGACGACTTTCATCGCTTTCCTAATAATGAGTGCGTACTCACTCATAATCAAGTTATATCATAAAGCCTCCGGGATGGGGCTTTGCTGCGAAAAAAAAAAACACACCCGCGCGGGG

>ramR-19 CCCTATCAGGCTAGCAGTGTTCGGTAACGGGTAGGTCAGGGCGATACGGTGAGCGCAGGGATGCAGTGTTTCCGGCGTCATTATGCGTCCGCCTCATGCAGGGCGTGCCACATGGCTTCAAAGCCGAGGGCGATAATCTCCCGGGCGCGCTGCGGATCGTGGCTGGCGAATTCGATGGTGGTTTCCGCCAGCGACAGAAACAGGGCGTCGCCAAAGGCGCGGTACGCCTCGCTGAGGAATATCTCTTTCACCGACAGCTGGCACATTTCGTTGAGCTCCGGAAAGCTCTCTTTTACCTGGCGGCGGGTTTCGTCGGTGATGCGCTCGCTGAGCGCCATCCGGCGGATCGCTTTGTGCTCCATCGGGTTGCGCACGCCCCAGTCGATATAGCTGTTCCAGATATTGCGCGCGTTCTCTTTCGGGCGCTTCTCGTCCGGATCCAGCCCGGCGATCATTGTGCGCACCAGGCGCAGCTTAATCGCGAGGTACAGCTCGTTGAGCAACTCATCCTTGGTGGCGAAATAGCGAAACAGCGTTCCCTCGGCCACACCGGCGCTGCGGGCGATGGCCGACGTCGAGGCGGCTATGCCGGACTGGGCGAAAGCGCGGTGGCAGCTTCCAGTAACGCTTGCTTTTTATCTTCACTCTTTGGACGAGCCACTACTTTTTTCCTCACGCAGGTTAAACAAACCGCCGATCTTGGCACGTCTGACCAGGTCGCTGCAACGGCGGAACGCAAAAATTGAAAATCGTCTTGACGACTTTCATCGCTTTCCTAATAATGAGTGCGTACTCACTCATAATCAAGTTATATCATAAAGCCTCCGGGATGGGGGCTTTTGCTGTCGAAAAAATAAA

>ramR-20 TCGTTCTGGGCGCAGTGTTCGGTAACGGGTAGGTCAGGGCGATACGGTGAGCGCAGGGATGCAGTGTTTCCGGCGTCATTATGCGTCCGCCTCATGCAGGGCGTGCCACATGGCTTCAAAGCCGAGGGCGATAATCTCCCGGGCGCGCTGCGGATCGTGGCTGGCGAATTCGATGGTGGTTTCCGCCAGCGACAGAAACAGGGCGTCGCCAAAGGCGCGGTACGCCTCGCTGAGGAATATCTCTTTCACCGACAGCTGGCACATTTCGTTGAGCTCCGGAAAGCTCTCTTTTACCTGGCGGCGGGTTTCGTCGGTGATGCGCTCGCTGAGCGCCATCCGGCGGATCGCTTTGTGCTCCATCGGGTTGCGCACGCCCCAGTCGATATAGCTGTTCCAGATATTGCGCGCGTTCTCTTTCGGGCGCTTCTCGTCCGGATCCAGCCCGGCGATCATTGTGCGCACCAGGCGCAGCTTAATCGCGAGGTACAGCTCGTTGAGCAACTCATCCTTGGTGGCGAAATAGCGAAACAGCGTTCCCTCGGCCACACCGGCGCTGCGGGCGATGGCCGACGTCGAGGCGGCTATGCCGGACTGGGCGAAAGCCGCGGTGGCAGCTTCCAGTAACGCTTGCTTTTTATCTTCACTCTTTGGACGAGCCACTACTTTTTTCCTCACGCAGGTTTAAACAAACCGCCGATCTTGGCACGTCTGACCAGGTCGCTGCAACGGCGGAACGCAAAAATTGAAAATCGTCTTGACGACTTTCATCGCTTTCCTAATAATGAGTGCGTACTCACTCATAATCAAGTTATATCATAAAGCCTCCGGGATGGGGCTTTGCTGCAGAAAAAGAAAA

>ramR-23 CCTTCAGGTCGCAGTGTTCGGTAACGGGTAGGTCAGGGCGATACGGTGAGCGCAGGGATGCAGCATCTCAGGGGTCATTTGGCGTCCGCCTCATGCAGGGCGTGCCACATGGCTTCAAAGCCGAGGGCGATAATCTCCCGGGCGCGCTGCGGATCGTGGCTGGCGAATTCGATGGTGGTTTCCGCCAGCGACAGAAACAGGGCGTCGCCAAAGGCGCGGTACGCCTCGCTGAGGAATATCTCTTTCACCGACAGCTGGCACATTTCGTTGAGCTCCGGAAAGCTCTCTTTTACCTGGCGGCGGGTTTCGTCGGTGATGCGCTCGCTGAGCGCCATCCGGCGGATCGCTTTGTGCTCCATCGGGTTGCGCACGCCCCAGTCGATATAGCTGTTCCAGATATTGCGCGCGTTCTCTTTCGGGCGCTTCTCGTCCGGATCCAGCCCGGCGATCATTGTGCGCACCAGGCGCAGCTTAATCGCGAGGTACAGCTCGTTGAGCAACTCATCCTTGGTGGCGAAATAGCGTTCCCTCGGCCACACCGGCGCTGCGGGCGATGGCCGACGTCGAGGCGGCTATGCCGGACTGGGCGAAAGCCGCGGTGGCAGCTTCCAGTAACGCTTGCTTTTTATCTTCACTCTTTGGACGAGCCACTACTTTTTTCCTCACGCAGGTTTAAACAAACCGCCGATCTTGGCACGTCTGACCAGGTCGCTGCAACGGCGGAACGCAAAAATTGAAAATCGTCTTGACGACTTTCATCGCTTTCCTAATAATGAGTGCGTACTCACTCATAATCAAGTTATATCATAAAGCCTCCGGGATGGGGGTTTTGTTGGCGAAAAAAAA

>ramR-24 TCGTTATCTAGCAGTGTTCGGTAACGGGTAGGTCAGGGCGATACGGTGAGCGCAGGGATGCAGTGTTTCCGGCGTCATTATGCGTCCGCCTCATGCAGGGCGTGCCACATGGCTTCAAAGCCGAGGGCGATAATCTCCCGGGCGCGCTGCGGATCGTGGCTGGCGAATTCGATGGTGGTTTCCGCCAGCGACAGAAACAGGGCGTCGCCAAAGGCGCGGTACGCCTCGCTGAGGAATATCTCTTTCACCGACAGCTGGCACATTTCGTTGAGCTCCGGAAAGCTCTCTTTTACCTGGCGGCGGGTTTCGTCGGTGATGCGCTCGCTGAGCGCCATCCGGCGGATCGCTTTGTGCTCCATCGGGTTGCGCACGCCCCAGTCGATATAGCTGTTCCAGATATTGCGCGCGTTCTCTTTCGGGCGCTTCTCGTCCGGATCCAGCCCGGCGATCATTGTGCGCACCAGGCGCAGCTTAATCGCGAGGTACAGCTCGTTGAGCAACTCATCCTTGGTGGCGAAATAGCGAAACAGCGTTCCCTAGGCCACACCGGCGCTGCGGGCGATGGCCGACGTCGAGGCGGCTATGCCGGACTGGGCGAAAGCCGCGGTGGCAGCTTCCAGTAACGCTTGCTTTTTATCTTCACTCTTTGGACGAGCCACTACTTTTTTCCTCACGCAGGTTTAAACAAACCGCCGATCTTGGCACGTCTGACCAGGTCGCTGCAACGGCGGAACGCAAAAATTGAAAATCGTCTTGACGACTTTCATCGCTTTCCTAATAATGAGTGCGTACTCACTCATAATCAAGTTATATCATAAAGCCTCCGGGATGGGGCTTTGCTGGGAGAAAAAGAAAACAAGCGCGCGGGGGGAGCC

>ramR-25 GGGATAGGCTAGCAGTGTTCGGTAACGGGTAGGTCAGGGCGATACGGTGAGCGCAGGGATGCAGTGTTTCCGGCGTCATTAGGCGTCCGCCTCATGCAGGGCGTGCCACATGGCTTCAAAGCCGAGGGCGATAATCTCCCGGGCGCGCTGCGGATCGTGGCTGGCGAATTCGATGGTGGTTTCCGCCAGCGACAGAAACAGGGCGTCGCCAAAGGCGCGGTACGCCTCGCTGAGGAATATCTCTTTCACCGACAGCTGGCACATTTCGTTGAGCTCCGGAAAGCTCTCTTTTACCTGGCGGCGGGTTTCGTCGGTGATGCGCTCGCTGAGCGCCATCCGGCGGATCGCTTTGTGCTCCATCGGGTTGCGCACGCCCCAGTCGATATAGCTGTTCCAGATATTGCGCGCGTTCTCTTTCGGGCGCTTCTCGTCCGGATCCAGCCCGGCGATCATTGTGCGCACCAGGCGCAGCTTAATCGCGAGGTACAGCTCGTTGAGCAACTCATCCTTAGTGGCGAAATAGCGAAACAGCGTTCCCTCGGCCACACCGGCGCTGCGGGCGATGGCCGACGTCGAGGCGGCTATGCCGGACTGGGCGAAAGCCGCGGTGGCAGCTTCCAGTAACGCTTGCTTTTTATCTTCACTCTTTGGACGAGCCACTACTTTTTTCCTCACGCAGGTTTAAACAAACCGCCGATCTTGGCACGTCTGACCAGGTCGCTGCAACGGCGGAACGCAAAAATTGAAAATCGTCTTGACGACTTTCATCGCTTTCCTAATAATGAGTGCGTACTCACTCATAATCAAGTTATATCATAAAGCCTCCGGGATGGGGCTTTGCTGCGCGAAGTT

>ramR-26 873 GCGTTCTGGTAGCAGTGTTCGGTAACGGGTAGGTCAGGGCGATACGGTGAGCGCAGGGATGCAGTGTTTCCGGCGTCATTATGCGTCCGCCTCATGCAGGGCGTGCCACATGGCTTCAAAGCCGAGGGCGATAATCTCCCGGGCGCGCTGCGGATCGTGGCTGGCGAATTCGATGGTGGTTTCCGCCAGCGACAGAAACAGGGCGTCGCCAAAGGCGCGGTACGCCTCGCTGAGGAATGTCTCTTTCACCGACAGCTGGCACATTTCGTTGAGCTCCGGAAAGCTCTCTTTTACCTGGCGGCGGGTTTCGTCGGTGATGCGCTCGCTGAGCGCCATCCGGCGGATCGCTTTGTGCTCCATCGGGTTGCGCACGCCCCAGTCGATATAGCTGTTCCAGATATTGCGCGCGTTCTCTTTCGGGCGCTTCTCGTCCGGATCCAGCCCGGCGATCATTGTGCGCACCAGGCGCAGCTTAATCGCGAGGTGCAGCTCGTTGAGCAACTCATCCTTGGTGGCGAAATAGCGAAACAGCGTTCCCTCGGCCACACCGGCGCTGCGGGCGATGGCCGACGTCGAGGCGGCTATGCCGGACTGGGCGAAAGCCGCGGTGGCAGCTTCCAGTAACGCTTGCTTTTTATCTTCACTCTTTGGACGAGCCACTACTTTTTTCCTCACGCAGGTTTAAACAAACCGCCGATCTTGGCACGTCTGACCAGGTCGCTGCAACGGCGGAACGCAAAAATTGAAAATCGTCTTGACGACTTTCATCGCTTTCCTAATAATGAGTGCGTACTCACTCATAATCAAGTTATATCATAAAGCCTCCGGGATGGGGGCTTTTGCTGGCAGGAAAAAAAAAAACCCGGGGAAGCG

>ramR-27 TCGGTATCTAGCAGTGTTCGGTAACGGGTAGGTCAGGGCGATACGGTGAGCGCAGGGATGCAGTGTTTCCGGCGTCATTAGGCGTCCGCCTCATGCAGGGCGTGCCACATGGCTTCAAAGCCGAGGGCGATAATCTCCCGGGCGCGCTGCGGATCGTGGCTGGCGAATTCGATGGTGGTTTCCGCCAGCGACAGAAACAGGGCGTCGCCAAAGGCGCGGTACGCCTCGCTGAGGAATGTCTCTTTCACCGACAGCTGGCACATTTCGTTGAGCTCCGGAAAGCTCTCTTTTACCTGGCGGCGGGTTTCGTCGGTGATGCGCTCGCTGAGCGCCATCCGGCGGATCGCTTTGTGCTCCATCGGGTTGCGCACGCCCCAGTCGATATAGCTGTTCCAGATATTGCGCGCGTTCTCTTTCGGGCGCTTCTCGTCTGGATCCAGCCCGGCGATCATTGTGCGCACCAGGCGCAGCTTAATCGCGAGGTACAGCTCGTTGAGCAACTCATCCTTGGTGGCGAAATAGCGAAACAGCGTTCCCTCGGCCACACCGGCGCTGCGGGCGATGGCCGACGTCGAGGCGGCTATGCCGGACTGGGCGAAAGCCGCGGTGGCAGCTTCCAGTAACGCTTGCTTTTTATCTTCACTCTTTGGACGAGCCACTACTTTTTTCCTCACGCAGGTTTAAACAAACCGCCGATCTTGGCACGTCTGACCAGGTCGCTGCAACGGCGGAACGCAAAAATTGAAAATCGTCTTGACGACTTTCATCGCTTTCCTAATAATGAGTGCGTACTCACTCATAATCAAGTTATATCATAAAGCCTCCGGGATGGGGGCTTTGCTGCGAAAAAAAAAAAACACACGCGGGGGAAAGG

>ramR-28 GCGATATGCTAGCAGTGTTCGGTAACGGGTAGGTCAGGGCGATACGGTGAGCGCAGGGATGCAGTGTTTCCGGCGTCATTAGGCGTCCGCCTCATGCAGGGCGTGCCACATGGCTTCAAAGCCGAGGGCGATAATCTCCCGGGCGCGCTGCGGATCGTGGCTGGCGAATTCGATGGTGGTTTCCGCCAGCGACAGAAACAGGGCGTCGCCAAAGGCGCGGTACGCCTCGCTGAGGAATGTCTCTTTCACCGACAGCTGGCACATTTCGTTGAGCTCCGGAAAGCTCTCTTTTACCTGGCGGCGGGTTTCGTCGGTGATGCGCTCGCTGAGCGCCATCCGGCGGATCGCTTTGTGCTCCATCGGGTTGCCGCACGCCCCAGTCGATATAGCTGTTCCAGATATTGCGCGCGTTCTCTTTCGGGCGCTTCTCGTCCGGATCCAGCCCGGCGATCATTGTGCGCACCAGGCGCAGCTTAATCGCGAGGTACAGCTCGTTGAGCAACTCATCCTTGGTGGCGAAATAGCGAAACAGCGTTCCCTCGGCCACACCGGCGCTGCGGGCGATGGCCGACGTCGAGGCGGCTATGCCGGACTGGGCGAAAGCCGCGGTGGCAGCTTCCAGTAACGCTTGCTTTTTATCTTCACTCTTTGGACGAGCCACTACTTTTTTCCTCACGCAGGTTTAAACAAACCGCCGATCTTGGCACGTCTGACCAGGTCGCTGCAACGGCGGAACGCAAAAATTGAAAATCGTCTTGACGACTTTCATCGCTTTCCTAATAATGAGTGCGTACTCACTCATAATCAAGTTATATCATAAAGCCTCCGGGATGGGGGCTTTGCTGCGGAGGGAA

>ramR-30 GGCGCAGCAGTGTTCGGTAACGGGTAGCTCAGGGCGATACGGTGAGCGCAGGGATGCAGTGTTTCCGGCGTCATTAGGCGTCCGCCTCATGCAGGGCGTGCCACATGGTTTCAAAGCCGAGGGCGATAATCTCCCGGGCGCGCTGCGGATCGTGGCTGGCGAATTCGATGGTGGTTTCCGCCAGCGACAGAAACAGGGCGTCGTCAAAGGCGCGGCACGCCTCGCTGAGGAATATCTCTTTCACCGACAGCTGGCACATTTCGTTGAGCTCCGGAAAGCTCTCTTTTACCTGGCGGCGGGTTTCGTCGGTGATGCGCTCGCTGAGCGCCATCCGGCGGATCGCTTTGTGCTCCATCGGGTTGCGCACGCCCCAGTCGATATAGCTGTTCCAGATATTGCGCGCGTTCTCTTTCGGGCGCTTCTCGTCCGGATCCAGCCCGGCGATCATTGTGCGCACCAGGCGCAGCTTAATCGCGAGGTACAGCTCGTTGAGCAACTCATCCTTGGTGGCGAAATAGCGAAACAGCGTTCCCTCGGCCACACCGGCGCTGCGGGCGATGGCCGACGTCGAGGCGGCTATGCCGGACTGGGCGAAAGCCGCGGTGGCAGCTTCCAGTAACGCTTGCTTTTTATCTTCACTCTTTGGACGAGCCACTACTTTTTTCCTCACGCAGGTTTAAACAAACCGCCGATCTTGGCACGTCTGACCAGGTCGCTGCAACGGCGGAACGCAAAAATTGAAAATCGTCTTGACGACTTTCATCGCTTTCCTAATAATGAGTGCGTACTCACTCATAATCAAGTTATATCATAAAGCCTCCGGGATGTGGTCTTTTGTCTGGTCAGGAAAATGAACCGGGGAACCGGAAAG

>tetA-2

GTGAAACCCAACAGACCCCTGATCGTAATTCTGAGCACTGTCGCGCTCGACGCTGTCGGCATCGGCCTGATTATGCCGGTGCTGCCGGGCCTCCTGCGCGATCTGGTTCACTCGAACGACGTCACCGCCCACTATGGCATTCTGCTGGCGCTGTATGCGTTGATGCAATTTGCCTGCGCACCTGTGCTGGGCGCGCTGTCGGATCGTTTCGGGCGGCGGCCGGTCTTGCTCGTCTCGCTGGCCGGCGCTGCTGTCGACTACGCCATCATGGCGACGGCGCCTTTCCTTTGGGTTCTCTATATCGGGCGGATCGTGGCCGGCATCACCGGGGCGACTGGGGCGGTAGCCGGCGCTTATATTGCCGATATCACTGATGGCGATGAGCGCGCGCGGCACTTCGGCTTCATGAGCGCCTGTTTCGGGTTCGGGATGGTCGCGGGACCTGTGCTCGGTGGGCTGATGGGCGGTTTCTCCCCCCACGCTCCGTTCTTCGCCGCGGCAGCCTTGAACGGCCTCAATTTCCTGACGGGCTGTTTCCTTTTGCCGGAGTCGCACAAAGGCGAACGCCGGCCGTTACGCCGGGAGGCTCTCAACCCGCTCGCTTCGTTCCGGTGGGCCCGGGGCATGACCGTCGTCGCCGCCCTGATGGCGGTCTTCTTCATCATGCAACTTGTCGGACAGGTGCCGGCCGCGCTTTGGGTCATTTTCGGCGAGGATCGCTTTCACTGGGACGCGACCACGATCGGCATTTCGCTTGCCGCATTTGGCATTCTGCATTCACTCGCCCAGGCAATGATCACCGGCCCTGTAGCCGCCCGGCTCGGCGAAAGGCGGGCACTCATGCTCGGAATGATTGCCGACGGCACAGGCTACATCCTGCTTGCCTTCGCGACACGGGGATGGATGGCGTTCCCGATCATGGTCCTGCTTGCTTCGGGTGGCATCGGAATGCCGGCGCTGCAAGCAATGTTGTCCAGGCAGGTGGATGAGGAACGTCAGGGGCAGCTGCAAGGCTCACTGGCGGCGCTCACCAGCCTGACCTCGATCGTCGGACCCCTCCTCTTCACGGCGATCTATGCGGCTTCTATAACAACGTGGAACGGGTGGGCATGGATTGCAGGCGCTGCCCTCTACTTGCTCTGCCTGCCGGCGCTGCGTCGCGGGCTTTGGAGCGGCGCAGGGCAACGAGCCGATCGCTGA

>tetA-3

GTGAAACCCAACAGACCCCTGATCGTAATTCTGAGCACTGTCGCGCTCGACGCTGTCGGCATCGGCCTGATTATGCCGGTGCTGCCGGGCCTCCTGCGCGATCTGGTTCACTCGAACGACGTCACCGCCCACTATGGCATTCTGCTGGCGCTGTATGCGTTGATGCAATTTGCCTGCGCACCTGTGCTGGGCGCGCTGTCGGATCGTTTCGGGCGGCGGCCGGTCTTGCTCGTCTCGCTGGCCGGCGCTGCTGTCGACTACGCCATCATGGCGACGGCGCCTTTCCTTTGGGTTCTCTATATCGGGCGGATCGTGGCCGGCATCACCGGGGCGACTGGGGCGGTAGCCGGCGCTTATATTGCCGATATCACTGATGGCGATGAGCGCGCGCGGCACTTCGGCTTCATGAGCGCCTGTTTCGGGTTCGGGATGGTCGCGGGACCTGTGCTCGGTGGGCTGATGGGCGGTTTCTCCCCCCACGCTCCGTTCTTCGCCGCGGCAGCCTTGAACGGCCTCAATTTCCTGACGGGCTGTTTCCTTTTGCCGGAGTCGCACAAAGGCGAACGCCGGCCGTTACGCCGGGAGGCTCTCAACCCGCTCGCTTCGTTCCGGTGGGCCCGGGGCATGACCGTCGTCGCCGCCCTGATGGCGGTCTTCTTCATCATGCAACTTGTCGGACAGGTGCCGGCCGCGCTTTGGGTCATTTTCGGCGAGGATCGCTTTCACTGGGACGCGACCACGATCGGCATTTCGCTTGCCGCATTTGGCATTCTGCATTCACTCGCCCAGGCAATGATCACCGGCCCTGTAGCCGCCCGGCTCGGCGAAAGGCGGGCACTCATGCTCGGAATGATTGCCGACGGCACAGGCTACATCCTGCTTGCCTTCGCGACACGGGGATGGATGGCGTTCCCGATCATGGTCCTGCTTGCTTCGGGTGGCATCGGAATGCCGGCGCTGCAAGCAATGTTGTCCAGGCAGGTGGATGAGGAACGTCAGGGGCAGCTGCAAGGCTCACTGGCGGCGCTCACCAGCCTGACCTCGATCGTCGGACCCCTCCTCTTCACGGCGATCTATGCGGCTTCTATAACAACGTGGAACGGGTGGGCATGGATTGCAGGCGCTGCCCTCTACTTGCTCTGCCTGCCGGCGCTGCGTCGCGGGCTTTGGAGCGGCGCAGGGCAACGAGCCGATCGCTGA

>tetA-7

GTGAAACCCAACAGACCCCTGATCGTAATTCTGAGCACTGTCGCGCTCGACGCTGTCGGCATCGGCCTGATTATGCCGGTGCTGCCGGGCCTCCTGCGCGATCTGGTTCACTCGAACGACGTCACCGCCCACTATGGCATTCTGCTGGCGCTGTATGCGTTGATGCAATTTGCCTGCGCACCTGTGCTGGGCGCGCTGTCGGATCGTTTCGGGCGGCGGCCGGTCTTGCTCGTCTCGCTGGCCGGCGCTGCTGTCGACTACGCCATCATGGCGACGGCGCCTTTCCTTTGGGTTCTCTATATCGGGCGGATCGTGGCCGGCATCACCGGGGCGACTGGGGCGGTAGCCGGCGCTTATATTGCCGATATCACTGATGGCGATGAGCGCGCGCGGCACTTCGGCTTCATGAGCGCCTGTTTCGGGTTCGGGATGGTCGCGGGACCTGTGCTCGGTGGGCTGATGGGCGGTTTCTCCCCCCACGCTCCGTTCTTCGCCGCGGCAGCCTTGAACGGCCTCAATTTCCTGACGGGCTGTTTCCTTTTGCCGGAGTCGCACAAAGGCGAACGCCGGCCGTTACGCCGGGAGGCTCTCAACCCGCTCGCTTCGTTCCGGTGGGCCCGGGGCATGACCGTCGTCGCCGCCCTGATGGCGGTCTTCTTCATCATGCAACTTGTCGGACAGGTGCCGGCCGCGCTTTGGGTCATTTTCGGCGAGGATCGCTTTCACTGGGACGCGACCACGATCGGCATTTCGCTTGCCGCATTTGGCATTCTGCATTCACTCGCCCAGGCAATGATCACCGGCCCTGTAGCCGCCCGGCTCGGCGAAAGGCGGGCACTCATGCTCGGAATGATTGCCGACGGCACAGGCTACATCCTGCTTGCCTTCGCGACACGGGGATGGATGGCGTTCCCGATCATGGTCCTGCTTGCTTCGGGTGGCATCGGAATGCCGGCGCTGCAAGCAATGTTGTCCAGGCAGGTGGATGAGGAACGTCAGGGGCAGCTGCAAGGCTCACTGGCGGCGCTCACCAGCCTGACCTCGATCGTCGGACCCCTCCTCTTCACGGCGATCTATGCGGCTTCTATAACAACGTGGAACGGGTGGGCATGGATTGCAGGCGCTGCCCTCTACTTGCTCTGCCTGCCGGCGCTGCGTCGCGGGCTTTGGAGCGGCGCAGGGCAACGAGCCGATCGCTGA

>tetA-8

GTGAAACCCAACAGACCCCTGATCGTAATTCTGAGCACTGTCGCGCTCGACGCTGTCGGCATCGGCCTGATTATGCCGGTGCTGCCGGGCCTCCTGCGCGATCTGGTTCACTCGAACGACGTCACCGCCCACTATGGCATTCTGCTGGCGCTGTATGCGTTGATGCAATTTGCCTGCGCACCTGTGCTGGGCGCGCTGTCGGATCGTTTCGGGCGGCGGCCGGTCTTGCTCGTCTCGCTGGCCGGCGCTGCTGTCGACTACGCCATCATGGCGACGGCGCCTTTCCTTTGGGTTCTCTATATCGGGCGGATCGTGGCCGGCATCACCGGGGCGACTGGGGCGGTAGCCGGCGCTTATATTGCCGATATCACTGATGGCGATGAGCGCGCGCGGCACTTCGGCTTCATGAGCGCCTGTTTCGGGTTCGGGATGGTCGCGGGACCTGTGCTCGGTGGGCTGATGGGCGGTTTCTCCCCCCACGCTCCGTTCTTCGCCGCGGCAGCCTTGAACGGCCTCAATTTCCTGACGGGCTGTTTCCTTTTGCCGGAGTCGCACAAAGGCGAACGCCGGCCGTTACGCCGGGAGGCTCTCAACCCGCTCGCTTCGTTCCGGTGGGCCCGGGGCATGACCGTCGTCGCCGCCCTGATGGCGGTCTTCTTCATCATGCAACTTGTCGGACAGGTGCCGGCCGCGCTTTGGGTCATTTTCGGCGAGGATCGCTTTCACTGGGACGCGACCACGATCGGCATTTCGCTTGCCGCATTTGGCATTCTGCATTCACTCGCCCAGGCAATGATCACCGGCCCTGTAGCCGCCCGGCTCGGCGAAAGGCGGGCACTCATGCTCGGAATGATTGCCGACGGCACAGGCTACATCCTGCTTGCCTTCGCGACACGGGGATGGATGGCGTTCCCGATCATGGTCCTGCTTGCTTCGGGTGGCATCGGAATGCCGGCGCTGCAAGCAATGTTGTCCAGGCAGGTGGATGAGGAACGTCAGGGGCAGCTGCAAGGCTCACTGGCGGCGCTCACCAGCCTGACCTCGATCGTCGGACCCCTCCTCTTCACGGCGATCTATGCGGCTTCTATAACAACGTGGAACGGGTGGGCATGGATTGCAGGCGCTGCCCTCTACTTGCTCTGCCTGCCGGCGCTGCGTCGCGGGCTTTGGAGCGGCGCAGGGCAACGAGCCGATCGCTGA

>tetA-10

GTGAAACCCAACAGACCCCTGATCGTAATTCTGAGCACTGTCGCGCTCGACGCTGTCGGCATCGGCCTGATTATGCCGGTGCTGCCGGGCCTCCTGCGCGATCTGGTTCACTCGAACGACGTCACCGCCCACTATGGCATTCTGCTGGCGCTGTATGCGTTGATGCAATTTGCCTGCGCACCTGTGCTGGGCGCGCTGTCGGATCGTTTCGGGCGGCGGCCGGTCTTGCTCGTCTCGCTGGCCGGCGCTGCTGTCGACTACGCCATCATGGCGACGGCGCCTTTCCTTTGGGTTCTCTATATCGGGCGGATCGTGGCCGGCATCACCGGGGCGACTGGGGCGGTAGCCGGCGCTTATATTGCCGATATCACTGATGGCGATGAGCGCGCGCGGCACTTCGGCTTCATGAGCGCCTGTTTCGGGTTCGGGATGGTCGCGGGACCTGTGCTCGGTGGGCTGATGGGCGGTTTCTCCCCCCACGCTCCGTTCTTCGCCGCGGCAGCCTTGAACGGCCTCAATTTCCTGACGGGCTGTTTCCTTTTGCCGGAGTCGCACAAAGGCGAACGCCGGCCGTTACGCCGGGAGGCTCTCAACCCGCTCGCTTCGTTCCGGTGGGCCCGGGGCATGACCGTCGTCGCCGCCCTGATGGCGGTCTTCTTCATCATGCAACTTGTCGGACAGGTGCCGGCCGCGCTTTGGGTCATTTTCGGCGAGGATCGCTTTCACTGGGACGCGACCACGATCGGCATTTCGCTTGCCGCATTTGGCATTCTGCATTCACTCGCCCAGGCAATGATCACCGGCCCTGTAGCCGCCCGGCTCGGCGAAAGGCGGGCACTCATGCTCGGAATGATTGCCGACGGCACAGGCTACATCCTGCTTGCCTTCGCGACACGGGGATGGATGGCGTTCCCGATCATGGTCCTGCTTGCTTCGGGTGGCATCGGAATGCCGGCGCTGCAAGCAATGTTGTCCAGGCAGGTGGATGAGGAACGTCAGGGGCAGCTGCAAGGCTCACTGGCGGCGCTCACCAGCCTGACCTCGATCGTCGGACCCCTCCTCTTCACGGCGATCTATGCGGCTTCTATAACAACGTGGAACGGGTGGGCATGGATTGCAGGCGCTGCCCTCTACTTGCTCTGCCTGCCGGCGCTGCGTCGCGGGCTTTGGAGCGGCGCAGGGCAACGAGCCGATCGCTGA

>tetA-11

GTGAAACCCAACAGACCCCTGATCGTAATTCTGAGCACTGTCGCGCTCGACGCTGTCGGCATCGGCCTGATTATGCCGGTGCTGCCGGGCCTCCTGCGCGATCTGGTTCACTCGAACGACGTCACCGCCCACTATGGCATTCTGCTGGCGCTGTATGCGTTGATGCAATTTGCCTGCGCACCTGTGCTGGGCGCGCTGTCGGATCGTTTCGGGCGGCGGCCGGTCTTGCTCGTCTCGCTGGCCGGCGCTGCTGTCGACTACGCCATCATGGCGACGGCGCCTTTCCTTTGGGTTCTCTATATCGGGCGGATCGTGGCCGGCATCACCGGGGCGACTGGGGCGGTAGCCGGCGCTTATATTGCCGATATCACTGATGGCGATGAGCGCGCGCGGCACTTCGGCTTCATGAGCGCCTGTTTCGGGTTCGGGATGGTCGCGGGACCTGTGCTCGGTGGGCTGATGGGCGGTTTCTCCCCCCACGCTCCGTTCTTCGCCGCGGCAGCCTTGAACGGCCTCAATTTCCTGACGGGCTGTTTCCTTTTGCCGGAGTCGCACAAAGGCGAACGCCGGCCGTTACGCCGGGAGGCTCTCAACCCGCTCGCTTCGTTCCGGTGGGCCCGGGGCATGACCGTCGTCGCCGCCCTGATGGCGGTCTTCTTCATCATGCAACTTGTCGGACAGGTGCCGGCCGCGCTTTGGGTCATTTTCGGCGAGGATCGCTTTCACTGGGACGCGACCACGATCGGCATTTCGCTTGCCGCATTTGGCATTCTGCATTCACTCGCCCAGGCAATGATCACCGGCCCTGTAGCCGCCCGGCTCGGCGAAAGGCGGGCACTCATGCTCGGAATGATTGCCGACGGCACAGGCTACATCCTGCTTGCCTTCGCGACACGGGGATGGATGGCGTTCCCGATCATGGTCCTGCTTGCTTCGGGTGGCATCGGAATGCCGGCGCTGCAAGCAATGTTGTCCAGGCAGGTGGATGAGGAACGTCAGGGGCAGCTGCAAGGCTCACTGGCGGCGCTCACCAGCCTGACCTCGATCGTCGGACCCCTCCTCTTCACGGCGATCTATGCGGCTTCTATAACAACGTGGAACGGGTGGGCATGGATTGCAGGCGCTGCCCTCTACTTGCTCTGCCTGCCGGCGCTGCGTCGCGGGCTTTGGAGCGGCGCAGGGCAACGAGCCGATCGCTGA

>tetA-13

GTGAAACCCAACAGACCCCTGATCGTAATTCTGAGCACTGTCGCGCTCGACGCTGTCGGCATCGGCCTGATTATGCCGGTGCTGCCGGGCCTCCTGCGCGATCTGGTTCACTCGAACGACGTCACCGCCCACTATGGCATTCTGCTGGCGCTGTATGCGTTGATGCAATTTGCCTGCGCACCTGTGCTGGGCGCGCTGTCGGATCGTTTCGGGCGGCGGCCGGTCTTGCTCGTCTCGCTGGCCGGCGCTGCTGTCGACTACGCCATCATGGCGACGGCGCCTTTCCTTTGGGTTCTCTATATCGGGCGGATCGTGGCCGGCATCACCGGGGCGACTGGGGCGGTAGCCGGCGCTTATATTGCCGATATCACTGATGGCGATGAGCGCGCGCGGCACTTCGGCTTCATGAGCGCCTGTTTCGGGTTCGGGATGGTCGCGGGACCTGTGCTCGGTGGGCTGATGGGCGGTTTCTCCCCCCACGCTCCGTTCTTCGCCGCGGCAGCCTTGAACGGCCTCAATTTCCTGACGGGCTGTTTCCTTTTGCCGGAGTCGCACAAAGGCGAACGCCGGCCGTTACGCCGGGAGGCTCTCAACCCGCTCGCTTCGTTCCGGTGGGCCCGGGGCATGACCGTCGTCGCCGCCCTGATGGCGGTCTTCTTCATCATGCAACTTGTCGGACAGGTGCCGGCCGCGCTTTGGGTCATTTTCGGCGAGGATCGCTTTCACTGGGACGCGACCACGATCGGCATTTCGCTTGCCGCATTTGGCATTCTGCATTCACTCGCCCAGGCAATGATCACCGGCCCTGTAGCCGCCCGGCTCGGCGAAAGGCGGGCACTCATGCTCGGAATGATTGCCGACGGCACAGGCTACATCCTGCTTGCCTTCGCGACACGGGGATGGATGGCGTTCCCGATCATGGTCCTGCTTGCTTCGGGTGGCATCGGAATGCCGGCGCTGCAAGCAATGTTGTCCAGGCAGGTGGATGAGGAACGTCAGGGGCAGCTGCAAGGCTCACTGGCGGCGCTCACCAGCCTGACCTCGATCGTCGGACCCCTCCTCTTCACGGCGATCTATGCGGCTTCTATAACAACGTGGAACGGGTGGGCATGGATTGCAGGCGCTGCCCTCTACTTGCTCTGCCTGCCGGCGCTGCGTCGCGGGCTTTGGAGCGGCGCAGGGCAACGAGCCGATCGCTGA

>tetA-14

GTGAAACCCAACAGACCCCTGATCGTAATTCTGAGCACTGTCGCGCTCGACGCTGTCGGCATCGGCCTGATTATGCCGGTGCTGCCGGGCCTCCTGCGCGATCTGGTTCACTCGAACGACGTCACCGCCCACTATGGCATTCTGCTGGCGCTGTATGCGTTGATGCAATTTGCCTGCGCACCTGTGCTGGGCGCGCTGTCGGATCGTTTCGGGCGGCGGCCGGTCTTGCTCGTCTCGCTGGCCGGCGCTGCTGTCGACTACGCCATCATGGCGACGGCGCCTTTCCTTTGGGTTCTCTATATCGGGCGGATCGTGGCCGGCATCACCGGGGCGACTGGGGCGGTAGCCGGCGCTTATATTGCCGATATCACTGATGGCGATGAGCGCGCGCGGCACTTCGGCTTCATGAGCGCCTGTTTCGGGTTCGGGATGGTCGCGGGACCTGTGCTCGGTGGGCTGATGGGCGGTTTCTCCCCCCACGCTCCGTTCTTCGCCGCGGCAGCCTTGAACGGCCTCAATTTCCTGACGGGCTGTTTCCTTTTGCCGGAGTCGCACAAAGGCGAACGCCGGCCGTTACGCCGGGAGGCTCTCAACCCGCTCGCTTCGTTCCGGTGGGCCCGGGGCATGACCGTCGTCGCCGCCCTGATGGCGGTCTTCTTCATCATGCAACTTGTCGGACAGGTGCCGGCCGCGCTTTGGGTCATTTTCGGCGAGGATCGCTTTCACTGGGACGCGACCACGATCGGCATTTCGCTTGCCGCATTTGGCATTCTGCATTCACTCGCCCAGGCAATGATCACCGGCCCTGTAGCCGCCCGGCTCGGCGAAAGGCGGGCACTCATGCTCGGAATGATTGCCGACGGCACAGGCTACATCCTGCTTGCCTTCGCGACACGGGGATGGATGGCGTTCCCGATCATGGTCCTGCTTGCTTCGGGTGGCATCGGAATGCCGGCGCTGCAAGCAATGTTGTCCAGGCAGGTGGATGAGGAACGTCAGGGGCAGCTGCAAGGCTCACTGGCGGCGCTCACCAGCCTGACCTCGATCGTCGGACCCCTCCTCTTCACGGCGATCTATGCGGCTTCTATAACAACGTGGAACGGGTGGGCATGGATTGCAGGCGCTGCCCTCTACTTGCTCTGCCTGCCGGCGCTGCGTCGCGGGCTTTGGAGCGGCGCAGGGCAACGAGCCGATCGCTGA

>tetA-17

GTGAAACCCAACAGACCCCTGATCGTAATTCTGAGCACTGTCGCGCTCGACGCTGTCGGCATCGGCCTGATTATGCCGGTGCTGCCGGGCCTCCTGCGCGATCTGGTTCACTCGAACGACGTCACCGCCCACTATGGCATTCTGCTGGCGCTGTATGCGTTGATGCAATTTGCCTGCGCACCTGTGCTGGGCGCGCTGTCGGATCGTTTCGGGCGGCGGCCGGTCTTGCTCGTCTCGCTGGCCGGCGCTGCTGTCGACTACGCCATCATGGCGACGGCGCCTTTCCTTTGGGTTCTCTATATCGGGCGGATCGTGGCCGGCATCACCGGGGCGACTGGGGCGGTAGCCGGCGCTTATATTGCCGATATCACTGATGGCGATGAGCGCGCGCGGCACTTCGGCTTCATGAGCGCCTGTTTCGGGTTCGGGATGGTCGCGGGACCTGTGCTCGGTGGGCTGATGGGCGGTTTCTCCCCCCACGCTCCGTTCTTCGCCGCGGCAGCCTTGAACGGCCTCAATTTCCTGACGGGCTGTTTCCTTTTGCCGGAGTCGCACAAAGGCGAACGCCGGCCGTTACGCCGGGAGGCTCTCAACCCGCTCGCTTCGTTCCGGTGGGCCCGGGGCATGACCGTCGTCGCCGCCCTGATGGCGGTCTTCTTCATCATGCAACTTGTCGGACAGGTGCCGGCCGCGCTTTGGGTCATTTTCGGCGAGGATCGCTTTCACTGGGACGCGACCACGATCGGCATTTCGCTTGCCGCATTTGGCATTCTGCATTCACTCGCCCAGGCAATGATCACCGGCCCTGTAGCCGCCCGGCTCGGCGAAAGGCGGGCACTCATGCTCGGAATGATTGCCGACGGCACAGGCTACATCCTGCTTGCCTTCGCGACACGGGGATGGATGGCGTTCCCGATCATGGTCCTGCTTGCTTCGGGTGGCATCGGAATGCCGGCGCTGCAAGCAATGTTGTCCAGGCAGGTGGATGAGGAACGTCAGGGGCAGCTGCAAGGCTCACTGGCGGCGCTCACCAGCCTGACCTCGATCGTCGGACCCCTCCTCTTCACGGCGATCTATGCGGCTTCTATAACAACGTGGAACGGGTGGGCATGGATTGCAGGCGCTGCCCTCTACTTGCTCTGCCTGCCGGCGCTGCGTCGCGGGCTTTGGAGCGGCGCAGGGCAACGAGCCGATCGCTGA

>tetA-18

GTGAAACCCAACAGACCCCTGATCGTAATTCTGAGCACTGTCGCGCTCGACGCTGTCGGCATCGGCCTGATTATGCCGGTGCTGCCGGGCCTCCTGCGCGATCTGGTTCACTCGAACGACGTCACCGCCCACTATGGCATTCTGCTGGCGCTGTATGCGTTGATGCAATTTGCCTGCGCACCTGTGCTGGGCGCGCTGTCGGATCGTTTCGGGCGGCGGCCGGTCTTGCTCGTCTCGCTGGCCGGCGCTGCTGTCGACTACGCCATCATGGCGACGGCGCCTTTCCTTTGGGTTCTCTATATCGGGCGGATCGTGGCCGGCATCACCGGGGCGACTGGGGCGGTAGCCGGCGCTTATATTGCCGATATCACTGATGGCGATGAGCGCGCGCGGCACTTCGGCTTCATGAGCGCCTGTTTCGGGTTCGGGATGGTCGCGGGACCTGTGCTCGGTGGGCTGATGGGCGGTTTCTCCCCCCACGCTCCGTTCTTCGCCGCGGCAGCCTTGAACGGCCTCAATTTCCTGACGGGCTGTTTCCTTTTGCCGGAGTCGCACAAAGGCGAACGCCGGCCGTTACGCCGGGAGGCTCTCAACCCGCTCGCTTCGTTCCGGTGGGCCCGGGGCATGACCGTCGTCGCCGCCCTGATGGCGGTCTTCTTCATCATGCAACTTGTCGGACAGGTGCCGGCCGCGCTTTGGGTCATTTTCGGCGAGGATCGCTTTCACTGGGACGCGACCACGATCGGCATTTCGCTTGCCGCATTTGGCATTCTGCATTCACTCGCCCAGGCAATGATCACCGGCCCTGTAGCCGCCCGGCTCGGCGAAAGGCGGGCACTCATGCTCGGAATGATTGCCGACGGCACAGGCTACATCCTGCTTGCCTTCGCGACACGGGGATGGATGGCGTTCCCGATCATGGTCCTGCTTGCTTCGGGTGGCATCGGAATGCCGGCGCTGCAAGCAATGTTGTCCAGGCAGGTGGATGAGGAACGTCAGGGGCAGCTGCAAGGCTCACTGGCGGCGCTCACCAGCCTGACCTCGATCGTCGGACCCCTCCTCTTCACGGCGATCTATGCGGCTTCTATAACAACGTGGAACGGGTGGGCATGGATTGCAGGCGCTGCCCTCTACTTGCTCTGCCTGCCGGCGCTGCGTCGCGGGCTTTGGAGCGGCGCAGGGCAACGAGCCGATCGCTGA

>tetA-21

GTGAAACCCAACAGACCCCTGATCGTAATTCTGAGCACTGTCGCGCTCGACGCTGTCGGCATCGGCCTGATTATGCCGGTGCTGCCGGGCCTCCTGCGCGATCTGGTTCACTCGAACGACGTCACCGCCCACTATGGCATTCTGCTGGCGCTGTATGCGTTGATGCAATTTGCCTGCGCACCTGTGCTGGGCGCGCTGTCGGATCGTTTCGGGCGGCGGCCGGTCTTGCTCGTCTCGCTGGCCGGCGCTGCTGTCGACTACGCCATCATGGCGACGGCGCCTTTCCTTTGGGTTCTCTATATCGGGCGGATCGTGGCCGGCATCACCGGGGCGACTGGGGCGGTAGCCGGCGCTTATATTGCCGATATCACTGATGGCGATGAGCGCGCGCGGCACTTCGGCTTCATGAGCGCCTGTTTCGGGTTCGGGATGGTCGCGGGACCTGTGCTCGGTGGGCTGATGGGCGGTTTCTCCCCCCACGCTCCGTTCTTCGCCGCGGCAGCCTTGAACGGCCTCAATTTCCTGACGGGCTGTTTCCTTTTGCCGGAGTCGCACAAAGGCGAACGCCGGCCGTTACGCCGGGAGGCTCTCAACCCGCTCGCTTCGTTCCGGTGGGCCCGGGGCATGACCGTCGTCGCCGCCCTGATGGCGGTCTTCTTCATCATGCAACTTGTCGGACAGGTGCCGGCCGCGCTTTGGGTCATTTTCGGCGAGGATCGCTTTCACTGGGACGCGACCACGATCGGCATTTCGCTTGCCGCATTTGGCATTCTGCATTCACTCGCCCAGGCAATGATCACCGGCCCTGTAGCCGCCCGGCTCGGCGAAAGGCGGGCACTCATGCTCGGAATGATTGCCGACGGCACAGGCTACATCCTGCTTGCCTTCGCGACACGGGGATGGATGGCGTTCCCGATCATGGTCCTGCTTGCTTCGGGTGGCATCGGAATGCCGGCGCTGCAAGCAATGTTGTCCAGGCAGGTGGATGAGGAACGTCAGGGGCAGCTGCAAGGCTCACTGGCGGCGCTCACCAGCCTGACCTCGATCGTCGGACCCCTCCTCTTCACGGCGATCTATGCGGCTTCTATAACAACGTGGAACGGGTGGGCATGGATTGCAGGCGCTGCCCTCTACTTGCTCTGCCTGCCGGCGCTGCGTCGCGGGCTTTGGAGCGGCGCAGGGCAACGAGCCGATCGCTGA

>tetA-22

GTGAAACCCAACAGACCCCTGATCGTAATTCTGAGCACTGTCGCGCTCGACGCTGTCGGCATCGGCCTGATTATGCCGGTGCTGCCGGGCCTCCTGCGCGATCTGGTTCACTCGAACGACGTCACCGCCCACTATGGCATTCTGCTGGCGCTGTATGCGTTGATGCAATTTGCCTGCGCACCTGTGCTGGGCGCGCTGTCGGATCGTTTCGGGCGGCGGCCGGTCTTGCTCGTCTCGCTGGCCGGCGCTGCTGTCGACTACGCCATCATGGCGACGGCGCCTTTCCTTTGGGTTCTCTATATCGGGCGGATCGTGGCCGGCATCACCGGGGCGACTGGGGCGGTAGCCGGCGCTTATATTGCCGATATCACTGATGGCGATGAGCGCGCGCGGCACTTCGGCTTCATGAGCGCCTGTTTCGGGTTCGGGATGGTCGCGGGACCTGTGCTCGGTGGGCTGATGGGCGGTTTCTCCCCCCACGCTCCGTTCTTCGCCGCGGCAGCCTTGAACGGCCTCAATTTCCTGACGGGCTGTTTCCTTTTGCCGGAGTCGCACAAAGGCGAACGCCGGCCGTTACGCCGGGAGGCTCTCAACCCGCTCGCTTCGTTCCGGTGGGCCCGGGGCATGACCGTCGTCGCCGCCCTGATGGCGGTCTTCTTCATCATGCAACTTGTCGGACAGGTGCCGGCCGCGCTTTGGGTCATTTTCGGCGAGGATCGCTTTCACTGGGACGCGACCACGATCGGCATTTCGCTTGCCGCATTTGGCATTCTGCATTCACTCGCCCAGGCAATGATCACCGGCCCTGTAGCCGCCCGGCTCGGCGAAAGGCGGGCACTCATGCTCGGAATGATTGCCGACGGCACAGGCTACATCCTGCTTGCCTTCGCGACACGGGGATGGATGGCGTTCCCGATCATGGTCCTGCTTGCTTCGGGTGGCATCGGAATGCCGGCGCTGCAAGCAATGTTGTCCAGGCAGGTGGATGAGGAACGTCAGGGGCAGCTGCAAGGCTCACTGGCGGCGCTCACCAGCCTGACCTCGATCGTCGGACCCCTCCTCTTCACGGCGATCTATGCGGCTTCTATAACAACGTGGAACGGGTGGGCATGGATTGCAGGCGCTGCCCTCTACTTGCTCTGCCTGCCGGCGCTGCGTCGCGGGCTTTGGAGCGGCGCAGGGCAACGAGCCGATCGCTGA

>tetA-23

GTGAAACCCAACAGACCCCTGATCGTAATTCTGAGCACTGTCGCGCTCGACGCTGTCGGCATCGGCCTGATTATGCCGGTGCTGCCGGGCCTCCTGCGCGATCTGGTTCACTCGAACGACGTCACCGCCCACTATGGCATTCTGCTGGCGCTGTATGCGTTGATGCAATTTGCCTGCGCACCTGTGCTGGGCGCGCTGTCGGATCGTTTCGGGCGGCGGCCGGTCTTGCTCGTCTCGCTGGCCGGCGCTGCTGTCGACTACGCCATCATGGCGACGGCGCCTTTCCTTTGGGTTCTCTATATCGGGCGGATCGTGGCCGGCATCACCGGGGCGACTGGGGCGGTAGCCGGCGCTTATATTGCCGATATCACTGATGGCGATGAGCGCGCGCGGCACTTCGGCTTCATGAGCGCCTGTTTCGGGTTCGGGATGGTCGCGGGACCTGTGCTCGGTGGGCTGATGGGCGGTTTCTCCCCCCACGCTCCGTTCTTCGCCGCGGCAGCCTTGAACGGCCTCAATTTCCTGACGGGCTGTTTCCTTTTGCCGGAGTCGCACAAAGGCGAACGCCGGCCGTTACGCCGGGAGGCTCTCAACCCGCTCGCTTCGTTCCGGTGGGCCCGGGGCATGACCGTCGTCGCCGCCCTGATGGCGGTCTTCTTCATCATGCAACTTGTCGGACAGGTGCCGGCCGCGCTTTGGGTCATTTTCGGCGAGGATCGCTTTCACTGGGACGCGACCACGATCGGCATTTCGCTTGCCGCATTTGGCATTCTGCATTCACTCGCCCAGGCAATGATCACCGGCCCTGTAGCCGCCCGGCTCGGCGAAAGGCGGGCACTCATGCTCGGAATGATTGCCGACGGCACAGGCTACATCCTGCTTGCCTTCGCGACACGGGGATGGATGGCGTTCCCGATCATGGTCCTGCTTGCTTCGGGTGGCATCGGAATGCCGGCGCTGCAAGCAATGTTGTCCAGGCAGGTGGATGAGGAACGTCAGGGGCAGCTGCAAGGCTCACTGGCGGCGCTCACCAGCCTGACCTCGATCGTCGGACCCCTCCTCTTCACGGCGATCTATGCGGCTTCTATAACAACGTGGAACGGGTGGGCATGGATTGCAGGCGCTGCCCTCTACTTGCTCTGCCTGCCGGCGCTGCGTCGCGGGCTTTGGAGCGGCGCAGGGCAACGAGCCGATCGCTGA

>tetA-24

GTGAAACCCAACAGACCCCTGATCGTAATTCTGAGCACTGTCGCGCTCGACGCTGTCGGCATCGGCCTGATTATGCCGGTGCTGCCGGGCCTCCTGCGCGATCTGGTTCACTCGAACGACGTCACCGCCCACTATGGCATTCTGCTGGCGCTGTATGCGTTGATGCAATTTGCCTGCGCACCTGTGCTGGGCGCGCTGTCGGATCGTTTCGGGCGGCGGCCGGTCTTGCTCGTCTCGCTGGCCGGCGCTGCTGTCGACTACGCCATCATGGCGACGGCGCCTTTCCTTTGGGTTCTCTATATCGGGCGGATCGTGGCCGGCATCACCGGGGCGACTGGGGCGGTAGCCGGCGCTTATATTGCCGATATCACTGATGGCGATGAGCGCGCGCGGCACTTCGGCTTCATGAGCGCCTGTTTCGGGTTCGGGATGGTCGCGGGACCTGTGCTCGGTGGGCTGATGGGCGGTTTCTCCCCCCACGCTCCGTTCTTCGCCGCGGCAGCCTTGAACGGCCTCAATTTCCTGACGGGCTGTTTCCTTTTGCCGGAGTCGCACAAAGGCGAACGCCGGCCGTTACGCCGGGAGGCTCTCAACCCGCTCGCTTCGTTCCGGTGGGCCCGGGGCATGACCGTCGTCGCCGCCCTGATGGCGGTCTTCTTCATCATGCAACTTGTCGGACAGGTGCCGGCCGCGCTTTGGGTCATTTTCGGCGAGGATCGCTTTCACTGGGACGCGACCACGATCGGCATTTCGCTTGCCGCATTTGGCATTCTGCATTCACTCGCCCAGGCAATGATCACCGGCCCTGTAGCCGCCCGGCTCGGCGAAAGGCGGGCACTCATGCTCGGAATGATTGCCGACGGCACAGGCTACATCCTGCTTGCCTTCGCGACACGGGGATGGATGGCGTTCCCGATCATGGTCCTGCTTGCTTCGGGTGGCATCGGAATGCCGGCGCTGCAAGCAATGTTGTCCAGGCAGGTGGATGAGGAACGTCAGGGGCAGCTGCAAGGCTCACTGGCGGCGCTCACCAGCCTGACCTCGATCGTCGGACCCCTCCTCTTCACGGCGATCTATGCGGCTTCTATAACAACGTGGAACGGGTGGGCATGGATTGCAGGCGCTGCCCTCTACTTGCTCTGCCTGCCGGCGCTGCGTCGCGGGCTTTGGAGCGGCGCAGGGCAACGAGCCGATCGCTGA

>tetA-25

GTGAAACCCAACAGACCCCTGATCGTAATTCTGAGCACTGTCGCGCTCGACGCTGTCGGCATCGGCCTGATTATGCCGGTGCTGCCGGGCCTCCTGCGCGATCTGGTTCACTCGAACGACGTCACCGCCCACTATGGCATTCTGCTGGCGCTGTATGCGTTGATGCAATTTGCCTGCGCACCTGTGCTGGGCGCGCTGTCGGATCGTTTCGGGCGGCGGCCGGTCTTGCTCGTCTCGCTGGCCGGCGCTGCTGTCGACTACGCCATCATGGCGACGGCGCCTTTCCTTTGGGTTCTCTATATCGGGCGGATCGTGGCCGGCATCACCGGGGCGACTGGGGCGGTAGCCGGCGCTTATATTGCCGATATCACTGATGGCGATGAGCGCGCGCGGCACTTCGGCTTCATGAGCGCCTGTTTCGGGTTCGGGATGGTCGCGGGACCTGTGCTCGGTGGGCTGATGGGCGGTTTCTCCCCCCACGCTCCGTTCTTCGCCGCGGCAGCCTTGAACGGCCTCAATTTCCTGACGGGCTGTTTCCTTTTGCCGGAGTCGCACAAAGGCGAACGCCGGCCGTTACGCCGGGAGGCTCTCAACCCGCTCGCTTCGTTCCGGTGGGCCCGGGGCATGACCGTCGTCGCCGCCCTGATGGCGGTCTTCTTCATCATGCAACTTGTCGGACAGGTGCCGGCCGCGCTTTGGGTCATTTTCGGCGAGGATCGCTTTCACTGGGACGCGACCACGATCGGCATTTCGCTTGCCGCATTTGGCATTCTGCATTCACTCGCCCAGGCAATGATCACCGGCCCTGTAGCCGCCCGGCTCGGCGAAAGGCGGGCACTCATGCTCGGAATGATTGCCGACGGCACAGGCTACATCCTGCTTGCCTTCGCGACACGGGGATGGATGGCGTTCCCGATCATGGTCCTGCTTGCTTCGGGTGGCATCGGAATGCCGGCGCTGCAAGCAATGTTGTCCAGGCAGGTGGATGAGGAACGTCAGGGGCAGCTGCAAGGCTCACTGGCGGCGCTCACCAGCCTGACCTCGATCGTCGGACCCCTCCTCTTCACGGCGATCTATGCGGCTTCTATAACAACGTGGAACGGGTGGGCATGGATTGCAGGCGCTGCCCTCTACTTGCTCTGCCTGCCGGCGCTGCGTCGCGGGCTTTGGAGCGGCGCAGGGCAACGAGCCGATCGCTGA

>tetA-26

GTGAAACCCAACAGACCCCTGATCGTAATTCTGAGCACTGTCGCGCTCGACGCTGTCGGCATCGGCCTGATTATGCCGGTGCTGCCGGGCCTCCTGCGCGATCTGGTTCACTCGAACGACGTCACCGCCCACTATGGCATTCTGCTGGCGCTGTATGCGTTGATGCAATTTGCCTGCGCACCTGTGCTGGGCGCGCTGTCGGATCGTTTCGGGCGGCGGCCGGTCTTGCTCGTCTCGCTGGCCGGCGCTGCTGTCGACTACGCCATCATGGCGACGGCGCCTTTCCTTTGGGTTCTCTATATCGGGCGGATCGTGGCCGGCATCACCGGGGCGACTGGGGCGGTAGCCGGCGCTTATATTGCCGATATCACTGATGGCGATGAGCGCGCGCGGCACTTCGGCTTCATGAGCGCCTGTTTCGGGTTCGGGATGGTCGCGGGACCTGTGCTCGGTGGGCTGATGGGCGGTTTCTCCCCCCACGCTCCGTTCTTCGCCGCGGCAGCCTTGAACGGCCTCAATTTCCTGACGGGCTGTTTCCTTTTGCCGGAGTCGCACAAAGGCGAACGCCGGCCGTTACGCCGGGAGGCTCTCAACCCGCTCGCTTCGTTCCGGTGGGCCCGGGGCATGACCGTCGTCGCCGCCCTGATGGCGGTCTTCTTCATCATGCAACTTGTCGGACAGGTGCCGGCCGCGCTTTGGGTCATTTTCGGCGAGGATCGCTTTCACTGGGACGCGACCACGATCGGCATTTCGCTTGCCGCATTTGGCATTCTGCATTCACTCGCCCAGGCAATGATCACCGGCCCTGTAGCCGCCCGGCTCGGCGAAAGGCGGGCACTCATGCTCGGAATGATTGCCGACGGCACAGGCTACATCCTGCTTGCCTTCGCGACACGGGGATGGATGGCGTTCCCGATCATGGTCCTGCTTGCTTCGGGTGGCATCGGAATGCCGGCGCTGCAAGCAATGTTGTCCAGGCAGGTGGATGAGGAACGTCAGGGGCAGCTGCAAGGCTCACTGGCGGCGCTCACCAGCCTGACCTCGATCGTCGGACCCCTCCTCTTCACGGCGATCTATGCGGCTTCTATAACAACGTGGAACGGGTGGGCATGGATTGCAGGCGCTGCCCTCTACTTGCTCTGCCTGCCGGCGCTGCGTCGCGGGCTTTGGAGCGGCGCAGGGCAACGAGCCGATCGCTGA

>tetA-21

GTGAAACCCAACAGACCCCTGATCGTAATTCTGAGCACTGTCGCGCTCGACGCTGTCGGCATCGGCCTGATTATGCCGGTGCTGCCGGGCCTCCTGCGCGATCTGGTTCACTCGAACGACGTCACCGCCCACTATGGCATTCTGCTGGCGCTGTATGCGTTGATGCAATTTGCCTGCGCACCTGTGCTGGGCGCGCTGTCGGATCGTTTCGGGCGGCGGCCGGTCTTGCTCGTCTCGCTGGCCGGCGCTGCTGTCGACTACGCCATCATGGCGACGGCGCCTTTCCTTTGGGTTCTCTATATCGGGCGGATCGTGGCCGGCATCACCGGGGCGACTGGGGCGGTAGCCGGCGCTTATATTGCCGATATCACTGATGGCGATGAGCGCGCGCGGCACTTCGGCTTCATGAGCGCCTGTTTCGGGTTCGGGATGGTCGCGGGACCTGTGCTCGGTGGGCTGATGGGCGGTTTCTCCCCCCACGCTCCGTTCTTCGCCGCGGCAGCCTTGAACGGCCTCAATTTCCTGACGGGCTGTTTCCTTTTGCCGGAGTCGCACAAAGGCGAACGCCGGCCGTTACGCCGGGAGGCTCTCAACCCGCTCGCTTCGTTCCGGTGGGCCCGGGGCATGACCGTCGTCGCCGCCCTGATGGCGGTCTTCTTCATCATGCAACTTGTCGGACAGGTGCCGGCCGCGCTTTGGGTCATTTTCGGCGAGGATCGCTTTCACTGGGACGCGACCACGATCGGCATTTCGCTTGCCGCATTTGGCATTCTGCATTCACTCGCCCAGGCAATGATCACCGGCCCTGTAGCCGCCCGGCTCGGCGAAAGGCGGGCACTCATGCTCGGAATGATTGCCGACGGCACAGGCTACATCCTGCTTGCCTTCGCGACACGGGGATGGATGGCGTTCCCGATCATGGTCCTGCTTGCTTCGGGTGGCATCGGAATGCCGGCGCTGCAAGCAATGTTGTCCAGGCAGGTGGATGAGGAACGTCAGGGGCAGCTGCAAGGCTCACTGGCGGCGCTCACCAGCCTGACCTCGATCGTCGGACCCCTCCTCTTCACGGCGATCTATGCGGCTTCTATAACAACGTGGAACGGGTGGGCATGGATTGCAGGCGCTGCCCTCTACTTGCTCTGCCTGCCGGCGCTGCGTCGCGGGCTTTGGAGCGGCGCAGGGCAACGAGCCGATCGCTGA

>tetA-22

GTGAAACCCAACAGACCCCTGATCGTAATTCTGAGCACTGTCGCGCTCGACGCTGTCGGCATCGGCCTGATTATGCCGGTGCTGCCGGGCCTCCTGCGCGATCTGGTTCACTCGAACGACGTCACCGCCCACTATGGCATTCTGCTGGCGCTGTATGCGTTGATGCAATTTGCCTGCGCACCTGTGCTGGGCGCGCTGTCGGATCGTTTCGGGCGGCGGCCGGTCTTGCTCGTCTCGCTGGCCGGCGCTGCTGTCGACTACGCCATCATGGCGACGGCGCCTTTCCTTTGGGTTCTCTATATCGGGCGGATCGTGGCCGGCATCACCGGGGCGACTGGGGCGGTAGCCGGCGCTTATATTGCCGATATCACTGATGGCGATGAGCGCGCGCGGCACTTCGGCTTCATGAGCGCCTGTTTCGGGTTCGGGATGGTCGCGGGACCTGTGCTCGGTGGGCTGATGGGCGGTTTCTCCCCCCACGCTCCGTTCTTCGCCGCGGCAGCCTTGAACGGCCTCAATTTCCTGACGGGCTGTTTCCTTTTGCCGGAGTCGCACAAAGGCGAACGCCGGCCGTTACGCCGGGAGGCTCTCAACCCGCTCGCTTCGTTCCGGTGGGCCCGGGGCATGACCGTCGTCGCCGCCCTGATGGCGGTCTTCTTCATCATGCAACTTGTCGGACAGGTGCCGGCCGCGCTTTGGGTCATTTTCGGCGAGGATCGCTTTCACTGGGACGCGACCACGATCGGCATTTCGCTTGCCGCATTTGGCATTCTGCATTCACTCGCCCAGGCAATGATCACCGGCCCTGTAGCCGCCCGGCTCGGCGAAAGGCGGGCACTCATGCTCGGAATGATTGCCGACGGCACAGGCTACATCCTGCTTGCCTTCGCGACACGGGGATGGATGGCGTTCCCGATCATGGTCCTGCTTGCTTCGGGTGGCATCGGAATGCCGGCGCTGCAAGCAATGTTGTCCAGGCAGGTGGATGAGGAACGTCAGGGGCAGCTGCAAGGCTCACTGGCGGCGCTCACCAGCCTGACCTCGATCGTCGGACCCCTCCTCTTCACGGCGATCTATGCGGCTTCTATAACAACGTGGAACGGGTGGGCATGGATTGCAGGCGCTGCCCTCTACTTGCTCTGCCTGCCGGCGCTGCGTCGCGGGCTTTGGAGCGGCGCAGGGCAACGAGCCGATCGCTGA

>tetA-27

GTGAAACCCAACAGACCCCTGATCGTAATTCTGAGCACTGTCGCGCTCGACGCTGTCGGCATCGGCCTGATTATGCCGGTGCTGCCGGGCCTCCTGCGCGATCTGGTTCACTCGAACGACGTCACCGCCCACTATGGCATTCTGCTGGCGCTGTATGCGTTGATGCAATTTGCCTGCGCACCTGTGCTGGGCGCGCTGTCGGATCGTTTCGGGCGGCGGCCGGTCTTGCTCGTCTCGCTGGCCGGCGCTGCTGTCGACTACGCCATCATGGCGACGGCGCCTTTCCTTTGGGTTCTCTATATCGGGCGGATCGTGGCCGGCATCACCGGGGCGACTGGGGCGGTAGCCGGCGCTTATATTGCCGATATCACTGATGGCGATGAGCGCGCGCGGCACTTCGGCTTCATGAGCGCCTGTTTCGGGTTCGGGATGGTCGCGGGACCTGTGCTCGGTGGGCTGATGGGCGGTTTCTCCCCCCACGCTCCGTTCTTCGCCGCGGCAGCCTTGAACGGCCTCAATTTCCTGACGGGCTGTTTCCTTTTGCCGGAGTCGCACAAAGGCGAACGCCGGCCGTTACGCCGGGAGGCTCTCAACCCGCTCGCTTCGTTCCGGTGGGCCCGGGGCATGACCGTCGTCGCCGCCCTGATGGCGGTCTTCTTCATCATGCAACTTGTCGGACAGGTGCCGGCCGCGCTTTGGGTCATTTTCGGCGAGGATCGCTTTCACTGGGACGCGACCACGATCGGCATTTCGCTTGCCGCATTTGGCATTCTGCATTCACTCGCCCAGGCAATGATCACCGGCCCTGTAGCCGCCCGGCTCGGCGAAAGGCGGGCACTCATGCTCGGAATGATTGCCGACGGCACAGGCTACATCCTGCTTGCCTTCGCGACACGGGGATGGATGGCGTTCCCGATCATGGTCCTGCTTGCTTCGGGTGGCATCGGAATGCCGGCGCTGCAAGCAATGTTGTCCAGGCAGGTGGATGAGGAACGTCAGGGGCAGCTGCAAGGCTCACTGGCGGCGCTCACCAGCCTGACCTCGATCGTCGGACCCCTCCTCTTCACGGCGATCTATGCGGCTTCTATAACAACGTGGAACGGGTGGGCATGGATTGCAGGCGCTGCCCTCTACTTGCTCTGCCTGCCGGCGCTGCGTCGCGGGCTTTGGAGCGGCGCAGGGCAACGAGCCGATCGCTGA

>tetA-30

GTGAAACCCAACAGACCCCTGATCGTAATTCTGAGCACTGTCGCGCTCGACGCTGTCGGCATCGGCCTGATTATGCCGGTGCTGCCGGGCCTCCTGCGCGATCTGGTTCACTCGAACGACGTCgCCGCCCACTATGGCATTCTGCTGGCGCTGTATGCGTTGATGCAATTTGCCTGCGCACCTGTGCTGGGCGCGCTGTCGGATCGTTTCGGGCGGCGGCCGGTCTTGCTCGTCTCGCTGGCCGGCGCTGCTGTCGACTACGCCATCATGGCGACGGCGCCTTTCCTTTGGGTTCTCTATATCGGGCGGATCGTGGCCGGCATCACCGGGGCGACTGGGGCGGTAGCCGGCGCTTATATTGCCGATATCACTGATGGCGATGAGCGCGCGCGGCACTTCGGCTTCATGAGCGCCTGTTTCGGGTTCGGGATGGTCGCGGGACCTGTGCTCGGTGGGCTGATGGGCGGTTTCTCCCCCCACGCTCCGTTCTTCGCCGCGGCAGCCTTGAACGGCCTCAATTTCCTGACGGGCTGTTTCCTTTTGCCGGAGTCGCACAAAGGCGAACGCCGGCCGTTACGCCGGGAGGCTCTCAACCCGCTCGCTTCGTTCCGGTGGGCCCGGGGCATGACCGTCGTCGCCGCCCTGATGGCGGTCTTCTTCATCATGCAACTTGTCGGACAGGTGCCGGCCGCGCTTTGGGTCATTTTCGGCGAGGATCGCTTTCACTGGGACGCGACCACGATCGGCATTTCGCTTGCCGCATTTGGCATTCTGCATTCACTCGCCCAGGCAATGATCACCGGCCCTGTAGCCGCCCGGCTCGGCGAAAGGCGGGCACTCATGCTCGGAATGATTGCCGACGGCACAGGCTACATCCTGCTTGCCTTCGCGACACGGGGATGGATGGCGTTCCCGATCATGGTCCTGCTTGCTTCGGGTGGCATCGGAATGCCGGCGCTGCAAGCAATGTTGTCCAGGCAGGTGGATGAGGAACGTCAGGGGCAGCTGCAAGGCTCACTGGCGGCGCTCACCAGCCTGACCTCGATCGTCGGACCCCTCCTCTTCACGGCGATCTATGCGGCTTCTATAACAACGTGGAACGGGTGGGCATGGATTGCAGGCGCTGCCCTCTACTTGCTCTGCCTGCCGGCGCTGCGTCGCGGGCTTTGGAGCGGCGCAGGGCAACGAGCCGATCGCTGA
